# Regime shifts of AMOC-sea surface temperature relationship

**DOI:** 10.1038/s41467-026-76149-4

**Published:** 2026-08-05

**Authors:** Yifei Fan, Duo Chan, Gokhan Danabasoglu, Who M. Kim, Pengfei Zhang, Laifang Li

**Affiliations:** 1 Department of Meteorology and Atmospheric Science, Pennsylvania State University, University Park, State College, PA USA; 2 School of Ocean and Earth Science, University of Southampton, Southampton, Hampshire, UK; 3 National Science Foundation National Center for Atmospheric Research, Boulder, CO USA; 4 State Key Laboratory of Physical Oceanography/College of Oceanic and Atmospheric Science, Ocean University of China, Qingdao, China; 5 Institute of Computational and Data Sciences, Pennsylvania State University, University Park, State College, PA USA; 6 Earth and Environmental Systems Institute, Pennsylvania State University, University Park, State College, PA USA

**Keywords:** Climate change, Physical oceanography

## Abstract

The Atlantic Meridional Overturning Circulation (AMOC) plays critical roles in regulating climate, and subpolar North Atlantic sea-surface temperature (SST) patterns are widely used to infer changes in its strength. Yet the stationarity of their relationship remains unclear. Here, using Community Earth System Model simulations spanning various climate states and a multi-model ensemble, we show that this relationship is state-dependent. We identify three distinct regimes: strong AMOC with the typical dipole fingerprint; intermediate AMOC with amplified subpolar SST anomalies; and weak AMOC with muted North Atlantic signals. These regimes arise primarily from changes in atmospheric radiative processes, while ocean processes contribute indirectly through air–sea interactions. The year of peak AMOC–SST sensitivity emerges as a predictor of the transition into the weak-AMOC regime and associated decay of the North Atlantic warming hole, offering a physically-based constraint on model uncertainties in climate projections. Our findings also imply that SST-based AMOC indicators must account for state dependence.

## Abstract (web summary)

Climate models reveal three distinct AMOC-dependent sea-surface temperature fingerprint regimes driven primarily by atmospheric radiative feedbacks, with peak AMOC–SST sensitivity emerging as a physically based predictor of the transition to a weak-AMOC state and a key constraint on 21st-century climate projection uncertainty.

## Introduction

The Atlantic Meridional Overturning Circulation (AMOC) is a crucial component of Earth’s climate system. This vast oceanic conveyor carries warm surface water northward and returns colder, denser water southward at depth^1^. Along the pathway, the ocean exchanges heat and carbon with the atmosphere, shaping regional and global climate^2–4^. As such, AMOC variability is linked to past major climate shifts^5,6^ and future global environmental changes^7^. An anomalously weak or strong AMOC is expected to result in a dipole sea surface temperature (SST) anomaly fingerprint by modulating poleward heat transport on decadal and longer time scales. For example, the subpolar North Atlantic cools as a weakening AMOC decreases poleward ocean heat transport in the upper ocean, whereas the western subtropics warms due to an associated northward migration of the Gulf Stream^8,9^.

**Fig. 1:**
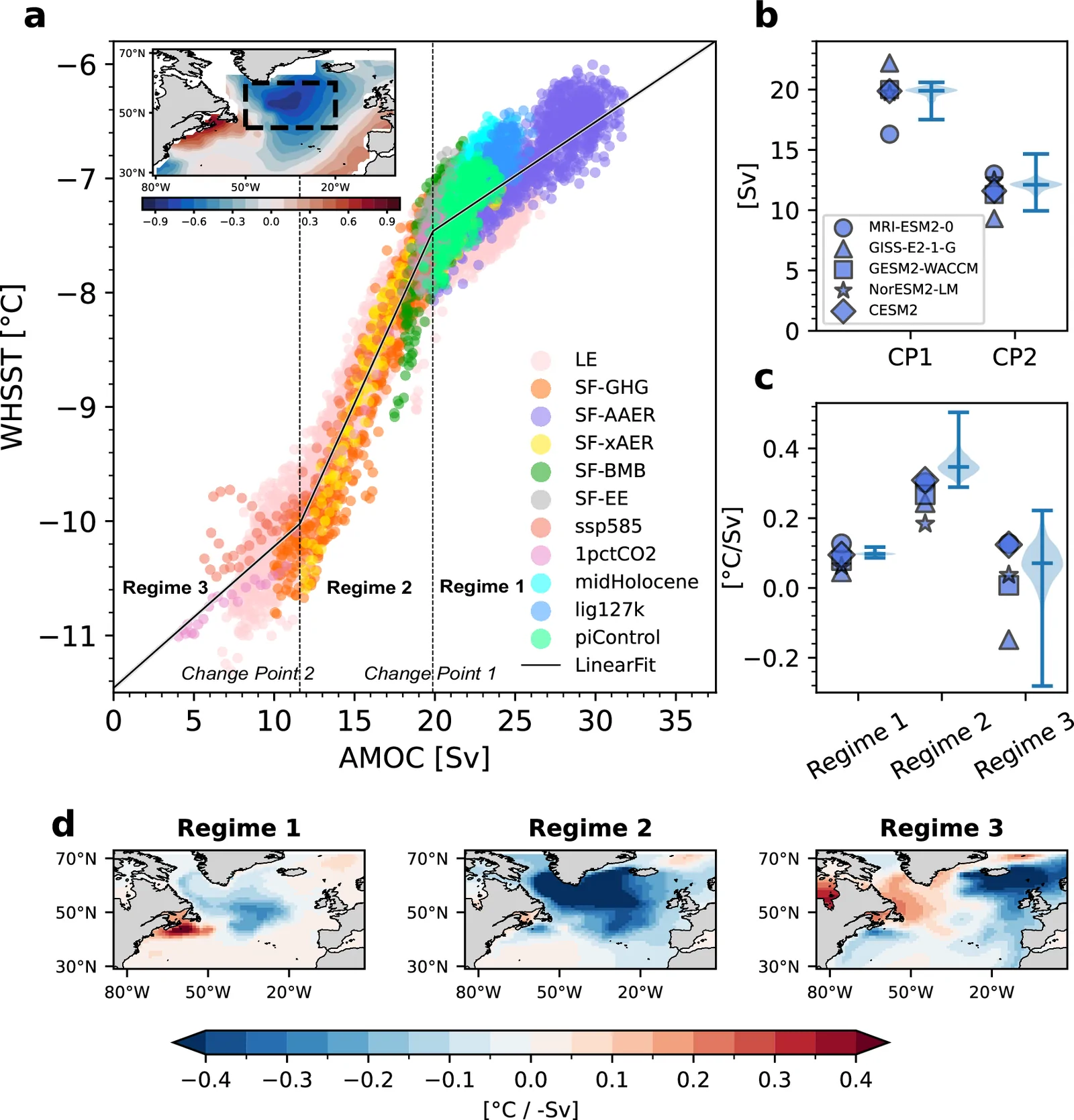
AMOC–North Atlantic SST relationship in model simulations. **a** Annual mean AMOC index (Sv) versus warming hole SST (WHSST, °C) in CESM2 simulations under various forcing conditions, indicated by different colors (see “Methods” for information about these simulations). The AMOC index is the maximum overturning transport below 500 m at 40° N; WHSST is the subpolar North Atlantic mean (45°–60°N, 20°–50°W; boxed in inset) minus the global mean. Data are smoothed with a 15-year window, with one point plotted every three years. Vertical dashed lines mark the two detected change points, dividing the relationship into three regimes. Black solid lines indicate the least squared regression with the change points. The inset shows observed SST trends minus global mean SST trend (1900–2014, °C century⁻¹) based on the average of three SST datasets as used in Fan et al. (2024). **b** Values of the two AMOC change points (CP1, CP2) in CESM2 and four other models (scatters). The violin plot (middle horizontal line, median; shade, density distribution; whiskers, range) represents the proxy distribution when change points are detected from a limited ensemble size rather than from large ensembles (“Methods”). **c** As in (b) but for WHSST sensitivity to AMOC (regression slope) across regimes. **d** Spatial patterns of SST sensitivity to AMOC weakening (–1 °C Sv⁻¹) in the three regimes, estimated by regressing SST anomalies relative to the global mean against AMOC (“Methods”).

Such an SST fingerprint of AMOC is widely used to infer past AMOC variability^8–11^ because direct AMOC measurements are scarce and challenging^12,13^. In particular, the observed North Atlantic warming hole–a lack of a long-term warming trend in the subpolar North Atlantic SST compared to global means (see the inserted map in Fig. 1a)–is hypothesized as evidence of a weakening AMOC over the 20^th^ century^9,11,14^. Such inferences, however, rely on the assumption that AMOC and North Atlantic SSTs have a stationary relationship across time.

Yet whether this assumption holds remains an open question. On decadal and longer timescales, AMOC-related oceanic heat transport (OHT) may shape extratropical North Atlantic SST variability^1,4,15,16^; however, other processes, such as gyre circulations, air–sea interactions, and atmospheric radiation, can also play important roles^17–21^. In fact, intensified gyre-driven OHT, strengthened surface winds, altered atmospheric circulation, and cloud radiative feedbacks have all been proposed as contributors to the warming hole^17–20^. If their influences do not vary proportionally with AMOC under different climate conditions, their combined effects on SST will vary and lead to a non-stationary AMOC–SST relationship^21–23^.

In this work, we address this question using large ensembles of state-of-the-art model simulations across a wide range of forcing scenarios. We identify three distinct regimes in the AMOC-SST relationship and show that as AMOC weakens the dominant driver of subpolar North Atlantic SST trends shifts from OHT to radiative processes. This regime dependence is a critical source of model spread in projecting North Atlantic SST changes by the end of the 21^st^ century.

## Results

### Three regimes in the AMOC-SST relationship

We first examine the relationship between AMOC and subpolar North Atlantic SSTs in a large compilation of simulations with the Community Earth System Model version 2 (CESM2; see “Methods”). These simulations span a broad range of climate states—from paleoclimate conditions, like the mid-Holocene and Last Interglacial warm periods, to 21st-century warming scenarios (Supplementary Table 1). We represent AMOC by its maximum transport below 500-m depth at 40°N (“Methods”). To isolate local SST anomalies more directly influenced by AMOC, we use a warming hole SST index (WHSST) defined as the SST difference between a regional mean in the subpolar North Atlantic where the observed warming hole is largely located (45°–60°N, 20°–50°W; inserted map in Fig. 1a) and the global mean (see “Methods”). A WHSST increase indicates locally amplified warming compared with elsewhere, whereas a decrease indicates development of a North Atlantic warming hole.

The AMOC strength varies substantially across different background climate states in the CESM2 simulations, and so does the sensitivity of WHSST to AMOC, quantified as the linear regression slope of WHSST against AMOC (Fig. 1a). Using an objective change-point detection algorithm (see “Methods”), we identify two change points (CPs) in the AMOC-WHSST sensitivity: one at ~20.0 Sv (CP1) and the other at ~11.5 Sv (CP2). The two CPs hence separate the AMOC-WHSST relationship into three regimes. In Regime 1, a strong AMOC ( > 20.0 Sv) corresponds to a low sensitivity of 0.09 °C Sv^−1^. In Regime 2, with an intermediate AMOC strength (11.5–20.0 Sv), the sensitivity increases more than threefold to 0.31 °C Sv^−1^. As AMOC weakens further (< 11.5 Sv), the sensitivity decreases again to 0.12 °C Sv^−1^ in Regime 3.

The regime-dependent AMOC-WHSST sensitivity is robust across different SST metrics, such as the one used in Caesar et al. (2018), and different AMOC metrics (see “Methods”; Supplementary Figs. 1, 2). It is also seen in at least four additional models from the Coupled Model Intercomparison Project phase 6 (CMIP6) archive (see “Methods”; Fig. 1b, c, Supplementary Fig. 3). Such consistency supports the robustness of the three distinct sensitivity regimes.

Associated with these regimes, North Atlantic SST changes display distinctly different spatial patterns (Fig. 1d). In Regime 1, the SST change exhibits a dipole structure: cooling in the central subpolar basin and warming along the Gulf Stream extension. This pattern is consistent with the typical SST fingerprint of a weaker-than-usual AMOC^8^ as observed (see the inserted map in Fig. 1a) and diagnosed in previous studies^9,17^. In Regime 2, SST cooling associated with AMOC weakening intensifies, covering the entire subpolar basin, with a local maximum sensitivity greater than 0.45 °C Sv^−1^ in the Irminger and Labrador Seas. In Regime 3, the cold SST anomalies, however, disappear in the western and central subpolar basin. Instead, they shift and emerge in the Norwegian Sea and the Iceland basin.

These distinct AMOC-WHSST sensitivity regimes and the associated non-stationary SST patterns together reveal three stages of the North Atlantic warming hole evolution: emerging in Regime 1, maturing in Regime 2, and decaying in Regime 3. Because historical and the 21^st^-century AMOC from CESM2 traverse these stages between 1985 and 2100, this regime framework maps directly onto near-term climate trajectories (Supplementary Figs. 4, 5a). It therefore provides an interpretable link between an AMOC decline, regional SST changes, and resulting impacts on the North Atlantic climate over the coming century.

### Underlying physical processes

**Fig. 2:**
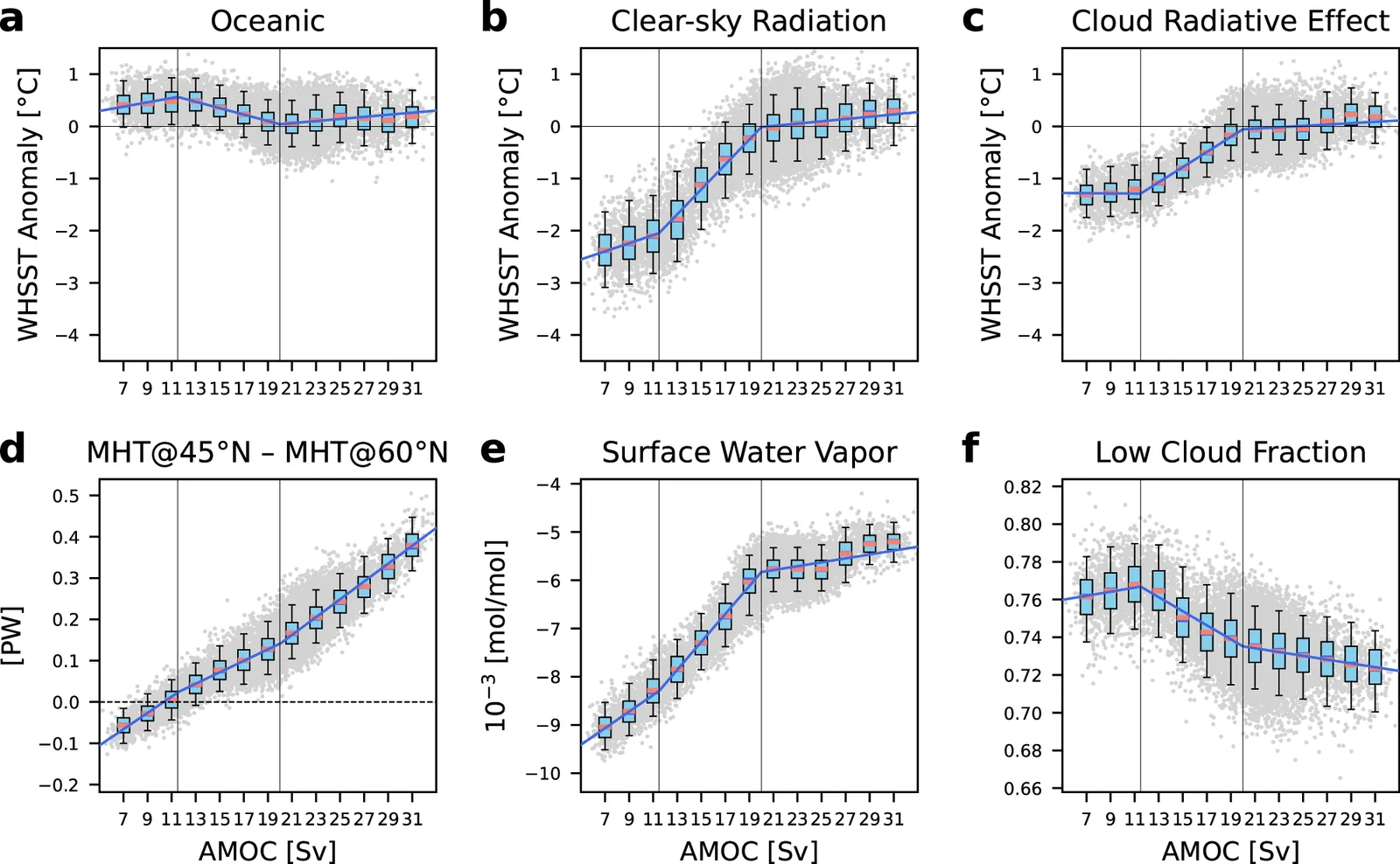
AMOC versus decomposed WHSST anomalies and associated variables. **a–c** AMOC index against WHSST anomalies arising from **a** oceanic processes (ocean heat transport convergence and surface turbulent fluxes), **b** clear-sky downward radiation, and **c** cloud radiative effect. Anomalies are relative to the 1850–1880 mean. **d**–**f** AMOC index against **d** the meridional heat transport (MHT) difference between 45°N and 60°N in the North Atlantic basin (unit: PW), approximating the subpolar North Atlantic OHT convergence, **e** the difference in surface water vapor between the subpolar North Atlantic and the global mean (mol mol⁻¹), and **f** subpolar North Atlantic low-cloud fraction. Data are from the 100-member full-forcing ensemble (LE) and four single-forcing ensembles (SF-AAER, SF-GHG, SF-BMB, and SF-EE). Gray dots denote annual values. Boxplots show statistics within 2-Sv AMOC bins (box, interquartile range; whiskers, 90% range; pink line, mean; blue line, median). Vertical lines mark the three AMOC regimes; blue regression lines show the AMOC–variable relationship within each regime.

**Fig. 3:**
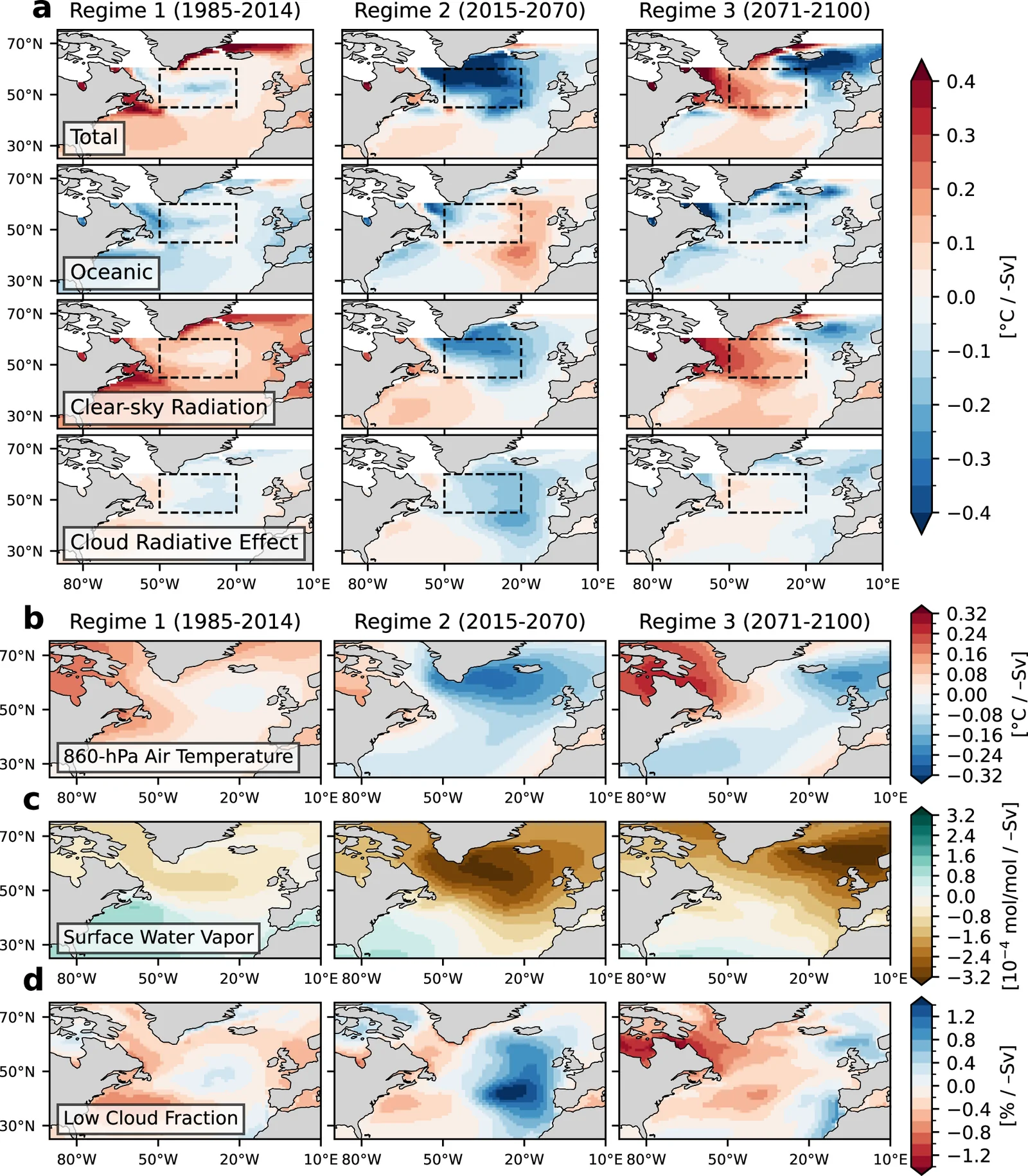
Spatial patterns of decomposed fingerprints and associated atmospheric changes. **a** AMOC fingerprints on SST anomalies due to different physical processes (rows) across the three regimes (columns). Fingerprints are estimated as CESM2-LE ensemble-mean ratios of SST trends to AMOC weakening rates over periods corresponding to each regime (“Methods”). Boxes denote the subpolar North Atlantic domain used for regional means in Fig. 2. **b**–**d** As in (**a**) but for AMOC fingerprints on (**b**) 860-hPa air temperature (global mean removed), **c** surface water vapor concentration (global mean removed), and **d** low-level cloud fraction.

To investigate the physical processes underlying the sensitivity changes, we apply a partial temperature change framework^24,25^ that decomposes WHSST anomalies in the radiatively forced 1850–2100 simulations into contributions from (1) oceanic processes, including the local heat storage and OHT, largely balanced by surface turbulent heat flux^26^, (2) surface downward clear-sky radiation dominated by the longwave component, and (3) cloud radiative effect (see “Methods”; Figs. 2, 3, Supplementary Figs. 6, 7).

Regime 1: The three process groups make similarly small contributions, each contributing roughly one-third to the modest AMOC–WHSST sensitivity of 0.09 °C Sv^−1^ (Fig. 2a–c). Net oceanic heat import minus turbulent heat loss, together with a slight cooling from cloud radiative effect, yields a net cooling over the subpolar domain with a weakening AMOC (Fig. 3a, left column). Surface downward clear-sky radiation—dominated by longwave flux from tropospheric air temperature (Fig. 3b) and water vapor (Fig. 3c)—together with the cloud radiative effect, provides a modest local warming that is weaker than in surrounding regions, allowing the dipole pattern to emerge.

Regime 2: The AMOC–WHSST sensitivity tripling to 0.31 °C Sv^−1^ is radiatively driven (Fig. 2b, c), and so is the warming hole intensification as AMOC weakens to an intermediate state (Fig. 3a, middle column). Clear-sky longwave flux supplies roughly two-thirds of the increased sensitivity (Fig. 2b; cf. clear-sky LW with clear-sky SW in Supplementary Fig. 6). Basin-wide tropospheric cooling (Fig. 3b) and moisture loss (Fig. 3c) reinforce the longwave anomaly, especially in the Irminger Sea. Enhanced shortwave cloud radiation effect accounts for most of the remaining sensitivity increase (Fig. 2c; cf. SW Cloud with LW Cloud in Supplementary Fig. 6). The increased reflection is due to low-level clouds (Figs. 2f, 3d), whereas mid- and high-level cloud effects are negligible (Supplementary Fig. 8). Oceanic terms remain nearly neutralized compared to radiative terms (Fig. 2a). The reduction in subpolar North Atlantic OHT convergence, which is proportional to the AMOC decline (Fig. 2d), is still offset by the extra decrease in the turbulent heat loss and therefore contributes little to the sensitivity increase compared to Regime 1.

Regime 3: The AMOC–WHSST sensitivity falls to 0.12 °C Sv^−1^ in the weak-AMOC regime because the effects of clear-sky radiation and clouds, both of which peaked in Regime 2, reduce significantly, while the net contributions from oceanic terms remain negligible (Fig. 2a–c). Despite the continuous weakening of AMOC, the subpolar North Atlantic region (45°–60°N, 20°–50°W) now warms with the globe. The set of mechanisms contributing to the mature warming hole in Regime 2 can still be seen, but their centers move northeastward into the Iceland–Norwegian sector (Fig. 3a, right column). Thus, the canonical North Atlantic warming hole decays because the radiative amplifiers vanish as their centers shift outside the canonical region.

Together, the three-regime structure comes from the atmosphere: clear-sky longwave and cloud shortwave fluxes together triple the sensitivity after CP1, then both collapse after CP2 (Fig. 2b, c) as the main cooling region shifts into the Iceland–Norwegian sector, causing the canonical North-Atlantic warming hole to fade (Fig. 3a). The non-stationary atmospheric changes with weakening AMOC could be a response to pre-existing SST anomalies or/and involve dynamical coupling between AMOC and atmospheric circulations.

Oceanic processes do not explicitly generate the three-regime structure but contribute implicitly. Sensitivity of subpolar North Atlantic OHT convergence to AMOC remains largely constant across regimes (Fig. 2d), despite potential non-stationarity in OHT sensitivity to AMOC across a single latitude^27^. Moreover, OHT convergence and turbulent heat loss largely cancel across all regimes, setting a near-neutral baseline that barely affects the AMOC–WHSST slope (Fig. 2a). Nevertheless, oceanic import makes an indirect contribution to the three-regime behavior through air-sea interaction^17–20^. By producing substantial SST anomalies, OHT convergence drives compensating adjustments in surface turbulent heat fluxes (Supplementary Fig. 6) that are associated with non-stationary atmospheric responses.

### Projected 21st-century regime shift

**Fig. 4:**
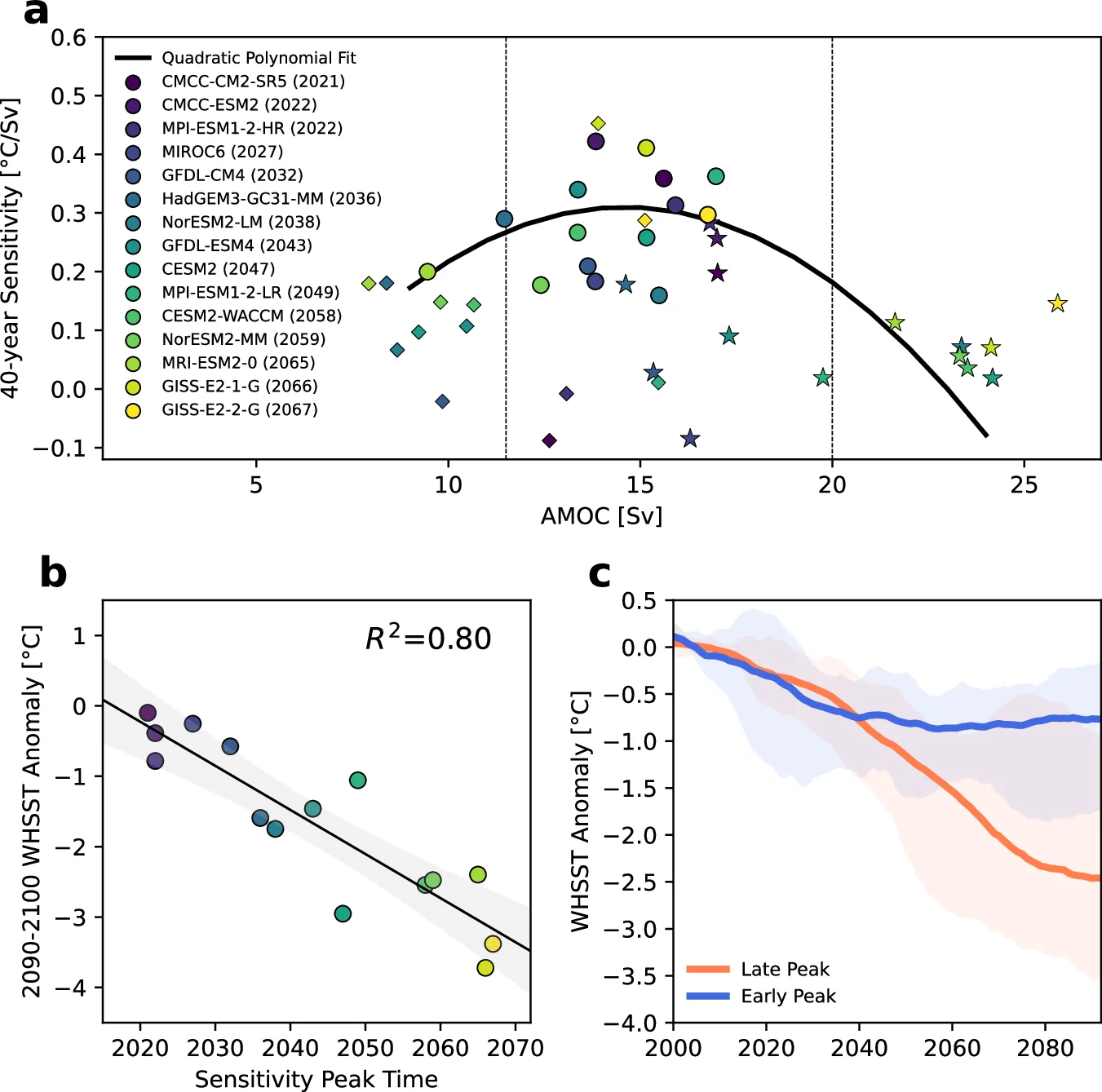
Projected 21^st^-century WHSST and its sensitivity to AMOC. **a** AMOC–WHSST sensitivity against mean-state AMOC in CESM2-LE (gray points) and in historical and SSP5-8.5 simulations by CMIP6 models (colored points; “Methods”). Sensitivity is estimated as the linear regression slope of the 15-year smoothed WHSST onto the 15-year smoothed AMOC index. The black line shows a quadratic fit between mean-state AMOC and sensitivity in CESM2-LE. Stars (diamonds) mark the 1980–2020 (2053–2093) values for each CMIP6 model. Dots indicate maximum sensitivity and the corresponding mean-state AMOC, with legend labels denoting the central year of the 40-year window (e.g., 2021 represents the 2001–2040 period). A 40-year window is chosen because it is long enough to capture decadal variability while still allowing a sufficient number of windows. The maximum sensitivity is identified after applying a 15-point smoothing to the sensitivity values sorted by mean-state AMOC strength. **b** Maximum sensitivity year against 2090–2100 WHSST anomalies (relative to 2000–2010) across the 15 CMIP6 models. The black line shows a linear fit, and gray shading denotes the 95% confidence interval. The maximum sensitivity year is defined as the first year in which the AMOC becomes weaker than the mean-state AMOC associated with the maximum sensitivity. **c** Fifteen-year smoothed WHSST anomalies relative to 2000–2010. The thick blue line shows the mean of seven models with maximum sensitivity before 2040 (early peak), and the red line shows the mean of the other eight (late peak). Shading denotes inter-model ranges.

Building on the physical processes identified above, we next examine how the three-regime framework manifests in future climate projections. As AMOC weakens under anthropogenic forcings, the system is expected to evolve along the non-stationary sensitivity trajectory, providing a dynamical basis for interpreting projected evolution of the North Atlantic warming hole. Specifically, in CESM2-LE, as the AMOC on average weakens continuously from about 24 Sv during 1980–2020 to less than 10 Sv in the end of this century, the 40-year moving sensitivity change follows an inverted-U-shaped trajectory (black line in Fig. 4a). Along this trajectory, increasing sensitivity with a weakening AMOC indicates the transition from Regime 1 to Regime 2, whereas decreasing sensitivity with a weakening AMOC marks the transition from Regime 2 to Regime 3.

CMIP6 models generally follow the CESM2-based inverted-U-shaped trajectory, despite differences in AMOC states and sensitivity values. Under the high-emission pathway (SSP5-8.5), 15 CMIP6 models project an AMOC decline together with a North Atlantic warming hole. They all show a tendency of transitioning towards Regime 3 in the end of the century, as denoted by a decrease in the sensitivity as AMOC becomes substantially weak (compare dots and diamonds in Fig. 4a). The projected timing of this transition, estimated by the year of the peak sensitivity, varies widely across the models (2021–2067; Fig. 4b). This spread explains 80% of the variance in the projected 2090–2100 WHSST anomalies (Fig. 4b), as an earlier transition to Regime 3 leads to an earlier decay of the warming hole (Fig. 4c). Although identifying precise change points in the real climate system is challenging, the timing of the maximum AMOC-SST sensitivity may provide a useful emergent indicator of the transition between regimes, particularly from the intermediate to weak-AMOC regime.

## Discussion

**Fig. 5:**
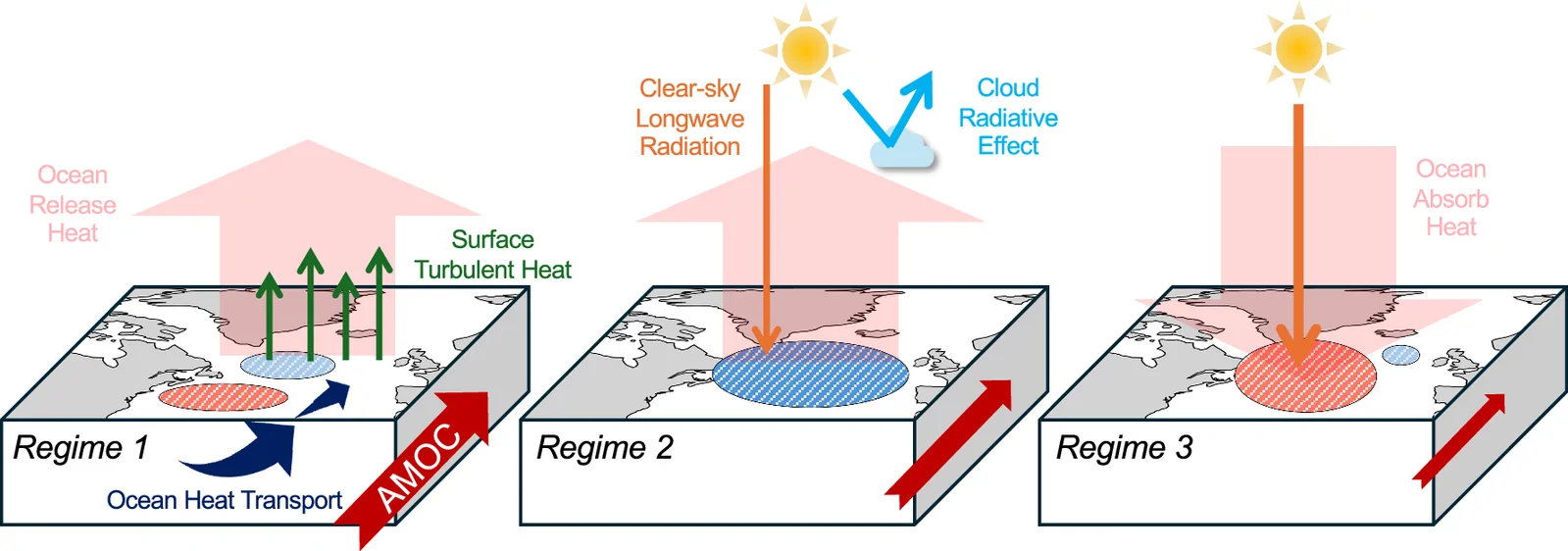
Conceptualized schematics of subpolar North Atlantic SST patterns and processes across regimes. Blue (red) patches indicate long-term SST cooling (warming) relative to the global mean. The red arrow denotes the AMOC, which weakens and shoals from Regime 1 to Regime 3. The pink arrow shows that by Regime 3, the net surface heat flux reverses sign, with the ocean absorbing heat from the atmosphere in the subpolar North Atlantic.

The relationship between the subpolar North Atlantic SST and AMOC is not stationary; instead, it falls into three distinct regimes that align with the emerging, maturing, and decaying stages of the North Atlantic warming hole. Each regime reflects a different balance among oceanic heat transport, air-sea heat exchange, and atmospheric radiative processes, with the relative importance of oceanic processes decreasing as AMOC weakens (summarized in Fig. 5). The three-regime structure originates from non-stationary atmospheric changes with weakening AMOC, while the ultimate causes driving such changes need further studies.

Beyond regime-by-regime contrasts in the energy balance, the locations of deep convection associated with an AMOC slowdown also shift across regimes (Supplementary Fig. 9). In the strong-AMOC regime, suppressed deep convection, as evidenced by reduced winter mixed layer depth, occurs mainly in the Labrador Sea. In the intermediate-AMOC regime, deep convection in the Irminger Sea also weakens. As the AMOC approaches CP2, deep convection effectively shuts down in both regions and subsequently begins to weaken in the Nordic Seas. Changes in North Atlantic deep convection are largely responses to surface buoyancy fluxes and winds associated with atmospheric circulations^28,29^. Although dedicated studies are required to determine the causes of these shifts in convection sites, we hypothesize that they are linked to changes in SST, salinity, and surface winds. As surface cooling and reduced evaporation shift northeastward from Regime 1 to Regime 3, the center of surface freshening follows (Supplementary Fig. 10). Meanwhile, as the AMOC weakens, surface winds decelerate over the Labrador and Irminger Seas in Regime 2, with the center of deceleration shifting to the Greenland–Iceland–Norwegian Sea in Regime 3 (Supplementary Fig. 11), possibly related to the warming hole’s influence on atmospheric circulation^30^. Overall, reduced surface buoyancy forcing from freshening, together with weakened wind forcing, suppresses local deep convection. These concurrent changes reflect coupled ocean–atmosphere–sea-ice interactions, highlighting the complexity of subpolar North Atlantic climate variability.

It would also be helpful to explore the ultimate drivers of the two change points. CP1 (strong to intermediate AMOC) coincides with the shift from aerosol-dominated to greenhouse-gas–dominated forcing (Supplementary Fig. 3a). As aerosol forcing weakens relative to rising greenhouse-gas concentrations, the reduction in cooling agents amplifies greenhouse-gas–induced tropospheric warming and moistening on the global scale. The resulting enhancement in clear-sky longwave radiation causes the AMOC–global mean SST slope (and, to a lesser extent, the AMOC–subpolar North Atlantic SST slope) to shift from near zero to negative values (Clear-sky LW in Supplementary Fig. 6a, b). Because the AMOC–subpolar North Atlantic SST slope becomes less negative than the AMOC–global mean SST slope, the AMOC–WHSST relationship steepens markedly after AMOC passes CP1. This tug-of-war between greenhouse gases and aerosols is present across all CESM2 simulations forced with historical (1850–2014) radiative forcings.

While CP1 is likely forcing dependent, CP2 (intermediate to weak AMOC) is forcing independent. In a freshwater-hosing run with radiative conditions fixed at the 1850 level, the North Atlantic SST still passes through Regime 2’s basin-wide cooling and into Regime 3’s pole-eastward retreat as AMOC weakens from 23 to 5 Sv (Supplementary Fig. 12). Additionally, CP2 may reflect a shift of the subpolar ocean from a net heat source to a net heat sink. Near CP2, the OHT convergence between 45°N and 60°N approaches zero (Fig. 2d), as more heat is transported out across the northern boundary than is transported in across the southern boundary. Such changes are associated with the vertical structures of temperature and meridional volume transport by ocean circulations. For example, the decaying phase of the North Atlantic warming hole has been linked with the northward shift of the mid-latitude jet and associated changes in gyre circulations^31^. Thus, the non-stationary AMOC–WHSST relationship can arise from both external-forcing shifts and intrinsic circulation thresholds.

Our findings have direct implications for the 21st-century North Atlantic climate projections. As the AMOC-SST sensitivity changes show an inverted-U-shaped trajectory with AMOC, the sensitivity peak time may provide a useful emergent indicator of the regime transition. Nevertheless, internal variability and model uncertainty make it challenging to predict the regime transition (Fig. 4). Internal variability might induce a short-term sensitivity change that temporarily masks the externally forced regime shift. As a result, the peak sensitivity can occur as early as 2021 and as late as 2067 across the 100-member CESM2-LE (Fig. 4a). To constrain models and reduce projection uncertainty, sustained AMOC observations are essential. Higher ocean model resolution is also needed, given its influence on the simulated AMOC mean state^32^. Moreover, scenario-based simulations extended beyond the 21^st^ century^33^ may provide additional insights into the AMOC-SST relationship.

Beyond future projections, our findings also have implications for reconstructing AMOC using SST-based proxy records, which often assume a stationary link between AMOC and its SST fingerprint. The pole-eastward shift of the cooling center identified in this study implies that paleo-reconstructions based on sparse, fixed-site records could underestimate late-stage AMOC variability. It also suggests that the optimal AMOC indicator might vary with AMOC states, further complicating the reconstruction during the era of instrumental observations^34^. The existence of three distinct regimes demonstrates the complexity of inferring dynamical changes from property variability^35^. In addition, uncertainty remains in detecting AMOC’s early warning signals in SST^36^. For example, an increased SST variance may reflect a diminishing role of oceanic processes and a strengthening role of atmospheric processes in driving SST variability, rather than an AMOC collapse. A more robust AMOC indicator would therefore require either wider proxy coverage that can follow the spatially migrating SST anomalies, or advanced statistical methods that explicitly account for the uncertainty introduced by the regime shifts.

## Methods

### CESM2 simulations

The Community Earth System Model version 2 **(**CESM2)^37^ is a fully coupled global Earth system model in which the Community Atmosphere Model version 6, the Parallel Ocean Program version 2, the Community Land Model version 5, and the Community Ice Code version 5 are coupled together at a nominal 1° horizontal resolution. The CESM2-Large Ensemble (LE) consists of 100 simulations covering the period from 1850–2100, which generally follows the historical and SSP3-7.0 forcing protocols provided by CMIP6^38^. Detailed experimental configuration of ensemble initialization and forcing fields is described in Rodgers et al.^39^.

The CESM2-Single Forcing (SF) ensemble^40^ consists of four sub-ensembles running from 1850 to 2050 under historical and SSP3-7.0 forcings, where only the forcings of interest are evolving in time, and the others are held fixed at their 1850s values. The four sub-ensembles are 1) a 15-member greenhouse gas (GHG) ensemble; 2) a 20-member anthropogenic aerosol (AAER) ensemble; 3) a 15-member biomass-burning aerosol (BMB) ensemble, and 4) a 15-member Everything Else (EE) ensemble. We also include another set of SF simulations that use an alternative method to address aerosol forcing: a 10-member fixed-AAER (xAER) ensemble running from 1920 to 2050 with all forcings evolving except for anthropogenic aerosols, which are held fixed at their 1920 values.

We have used five additional sets of simulations, all conducted following the CMIP6 protocols: 1) three SSP5-8.5 simulations; 2) a 1pctCO_2_ simulation; 3) a pre-industrial control simulation (piControl); 4) a paleoclimate simulation of the mid-Holocene, namely midHolocene; and 5) a paleoclimate simulation of the Last Interglacial, namely lig127k^41^. We have also examined one hosing simulation (u03-hos) where AMOC is forced to weaken by a uniform freshwater input of 0.3 Sv north of 50°N, with all external forcings fixed at 1850s conditions^42^. Information about these CESM2 simulations is summarized in Supplementary Table 1.

### AMOC strength and SST indices

The AMOC strength is represented by an AMOC index defined as the maximum volume transport below 500-m depth at 40°N in the North Atlantic. The volume transport is either a direct model output (e.g., MOC in the CESM2-LE) or converted from a standard CMIP6 model output variable named meridional mass overturning circulation (msftmz) with the assumption of a constant seawater density of 1027 kg/m^3^. While this index focuses on the strength of the maximum AMOC at 40°N in depth space, we acknowledge that AMOC can experience changes in other aspects, such as the shallowing and deepening of the upper overturning cell and water mass changes. In the CESM2 simulations analyzed here, the depth of the overturning cell and the maximum AMOC strength are positively correlated so that an AMOC weakening is accompanied by an AMOC shoaling (Supplementary Fig. 2f). Because AMOC variations are meridionally coherent on decadal and longer timescales^43^, the maximum overturning stream function at other latitudes (e.g., 50°N), or the maximum across all latitudes, all show two change points in AMOC-WHSST sensitivity, consistent with our AMOC index defined using the maximum overturning stream function at 40°N (Supplementary Fig. 2).

In the main text, we use a WHSST index to quantify subpolar North Atlantic SST changes that are dominantly influenced by AMOC. Similar indices–representing differences between a regional mean SST and a basin-, hemisphere-, or globe-averaged SST–are also commonly used to study such problems^9,11^. As such, we have additionally examined three alternative SST indices, including the one used by Caesar et al. (2018). They all yield consistent results for the two change points in the AMOC-WHSST relationship (Supplementary Fig. 1), demonstrating the robustness of the three regimes.

### Simulations by other models participating CMIP6

The non-stationary AMOC-WHSST relationship is also seen in compilations of simulations performed by four other models participating in CMIP6: MRI-ESM2-0^44^, GISS-E2-1-G^45^, CESM2-WACCM^46^, and NorESM2-LM^47^ (Supplementary Table 2). These simulations include pre-industrial control simulations (piControl), idealized 1pctCO_2_ simulations, paleoclimate simulations (lig127k and/or midHolocene), historical simulations, GHG-only and aerosol-only single-forcing simulations of the historical period (hist-GHG and/or hist-aer); and scenario-based 21^st^-century simulations (SSP3-7.0 and/or SSP5-8.5).

To examine the 21^st^-century evolution of the AMOC-WHSST sensitivity, we use the scenario-based simulations by an additional set of CMIP6 models (Supplementary Table 3). This set of models is chosen because their SSP5-8.5 simulations show a North Atlantic warming hole together with a weakening AMOC, thus satisfying our purpose of studying the warming hole evolution with an AMOC decline. Specifically, they meet two requirements: (1) AMOC weakens and reaches CP1 and/or CP2 by 2100; (2) WHSST shows a decreasing trend during the early periods of the 21^st^ century (Supplementary Fig. 13).

### Change-point detection and statistical fitting

To quantitatively describe the AMOC-WHSST relationship, we have tried using five fitting models and compared their performances. The five fitting models include a logarithmic fitting model with no CP and linear fitting models with zero to three CP(s). When identifying CPs in the AMOC-WHSST relationship, we use a genetic algorithm to efficiently find the CPs that minimize the mean squared error of the given fitting model. The algorithm begins by generating 1000 candidate CP combinations and evaluating their fitness using the mean-squared errors of the fitting. The first generation is given based on initial knowledge (e.g., the CPs are in the range from 5–33 Sv), to which the results are not sensitive. The next generation is produced by retaining the fittest 100 combinations, randomly mixing 450 combinations, and randomly creating 450 combinations. The process iterates until the best solution remains unchanged for 10 generations or a maximum of 100 iterations is reached. Detailed workflow of the algorithm is shown in Supplementary Fig. 14.

The performances of the five fitting models are indicated by the Bayesian information criterion (BIC). BIC consists of a log-likelihood term and a penalty term. The log-likelihood term, -$2{LL}\left(y,|,F\left(x\right),\,\epsilon \right)$, measures the probability of observing a given set of data, *y*, assuming a specific linear fitting model, $F\left(x\right)$, and a model error following normal distribution, ϵ. The penalty term, $p{{\mathrm{ln}}}N$, prevents an overfitted model with too many parameters, where *p* is the number of parameters in the statistical model, and *N* is the data length. The BIC score is calculated as

1 $${BIC}=-2{LL}\left(y | F\left(x\right),\,\epsilon \right)+p{{\mathrm{ln}}}N=N\left[{{\mathrm{ln}}}\left(2\pi \right)+{{\mathrm{ln}}}\left({\sigma }_{\epsilon }^{2}\right)+1\right]+p{{\mathrm{ln}}}N.$$

The lower the BIC score, the better the fitting. We have eventually chosen the linear model with two change points because it has a relatively low BIC score while remaining physically interpretable (Supplementary Fig. 15). For clarity and ease of interpretation, the detected CPs (11.59and 19.87) are rounded to 11.5 Sv and 20.0 Sv, respectively, as these simplified values lie within the uncertainty range and do not affect the conclusions.

The uncertainty in CP detection decreases with increasing sample size. However, large ensembles such as those from CESM2 are not available for all models, which limits direct inter-model comparison. To quantify how restricted sample size affects CP detection, we perform a subsampling analysis using the CESM2 LE and SF simulations. Specifically, we randomly select three members, which is comparable to the ensemble sizes of CMIP6 models, and repeat this procedure 1000 times. The resulting distributions of the two CPs and the corresponding sensitivities across the three regimes are summarized by the violin plots in Fig. 1b, c. Additional details are provided in the Supplementary Information.

### SST sensitivity to AMOC and its spatial patterns

The SST sensitivity to AMOC is estimated via two approaches. One is by directly regressing SST (e.g., WHSST) onto AMOC with the ordinary least squares method. Via this approach, we use all the examined CESM2 simulations to estimate the sensitivity of WHSST to AMOC and the corresponding spatial patterns in the three regimes under various forcing scenarios (e.g., in Fig. 1). We also use this approach to calculate the sensitivity over any 40-year period from 1850 to 2100 to derive its time-evolving trajectory (e.g., in Fig. 4).

We also indirectly estimate the sensitivity in individual simulations by calculating the ratio of the SST trend to the AMOC trend over a period. This approach assumes a linear AMOC-SST relationship, which is the case if the whole time period falls into a single sensitivity regime. We take this approach to examine the evolution of the North Atlantic warming hole and the role the AMOC plays in it through time under a certain forcing scenario. For example, in the CESM2-LE, the AMOC variations from 1850 to 2100 span all three regimes (Fig. 1a, Supplementary Fig. 4a). Before the 1980s, AMOC steadily strengthens, driven primarily by increasing anthropogenic aerosols, and peaks at 26.50 Sv ( ± 0.86) Sv; standard deviation across the 100-member ensemble. After this peak, it declines rapidly to 6.54 ( ± 0.52) Sv by 2100 in response to increasing greenhouse gas forcing. In other words, the AMOC transitions through the three regimes beginning in the 1980s: the strong regime from 1985–2014 (Regime 1), the intermediate regime from 2014–2070 (Regime 2), and the weak regime from 2071–2100 (Regime 3). Similarly, we can identify time periods corresponding to the three regimes in the AAER-only and GHG-only simulations. Results are insensitive to a shift of a few years in the boundary time (Supplementary Fig. 16).

These two methods have a major difference. The direct ordinary least square regression between AMOC and SST captures their relationship on all time scales, whereas the trend ratio approach emphasizes longer time scales, which is our focus in this study. Therefore, when using the direct regression method, all data are smoothed by a 15-year moving window in prior. Despite the difference, the two approaches yield consistent estimates of the fingerprint patterns (cf. Fig. 1d and Total in Fig. 3a, Supplementary Fig. 7).

### Temperature anomaly decomposition

For a water column extending from the sea surface to the ocean floor, the time rate of ocean heat content (OHC) within the whole column per unit aera is determined by the net surface downward heat flux, *Q*, and ocean heat transport (OHT) divergence, OHT, as shown by

2 $$\frac{\partial {OHC}}{\partial t}\,=\,Q\,-\,{{{\boldsymbol{\nabla }}}}\cdot {{{\bf{OHT}}}}.$$

The net surface downward heat flux is determined by surface downward shortwave radiation ($S{W}^{\downarrow }$), surface downward longwave radiation ($L{W}^{\downarrow }$), surface upward longwave radiation ($L{W}^{\uparrow }$), surface turbulent heat fluxes–latent heat (LH) and sensible heat (SH) fluxes, and heat fluxes due to ice and snow processes (${F}_{{ice}+{snow}}$). Therefore, *Q* has a full expansion as:

3 $$Q=\left(1-\alpha \right)S{W}^{\downarrow }+L{W}^{\downarrow }-L{W}^{\uparrow }-\left({LH}+{SH}\right)+{F}_{{ice}+{snow}},$$

where α is the surface albedo and all fluxes with a positive sign denote downward fluxes. Here, we focus on changes in annual means and assume the effect of snow and ice on the heat balance is negligible, that is, ${F}_{{ice}+{snow}}\approx 0$. This assumption is not applicable to polar and ice-covered regions, and so is the decomposition method (Supplementary Fig. 17). Therefore, these regions are masked off throughout our decomposition analysis.

According to the Stefan-Boltzmann law, the upward longwave radiation emitted by the sea surface, which presumably is a black body, is solely dependent on SST (denoted by *T*) as $L{W}^{\uparrow }={{{\rm{\sigma }}}}{T}^{4}$. Thus, rearranging the terms in Eq. (3) leads to

4 $${{{\rm{\sigma }}}}{T}^{4}=\left(1-\alpha \right)S{W}^{\downarrow }+L{W}^{\downarrow }-Q-\left({LH}+{SH}\right).$$

Consider each variable as a climatology (i.e., the 1850–1880 mean) plus an anomaly from the climatology, represented by $\bar{(\cdot )}$ and ${(\cdot )}^{{\prime} }$, respectively, and the left-hand side of Eq. (4) becomes ${{{\rm{\sigma }}}}{\bar{T}}^{4}+4{\bar{T}}^{3}{T}^{{\prime} }$, where the terms with the second and higher orders of ${T}^{{\prime} }$ are neglected; and the first right-hand side term of Eq. (4) becomes $\left(1-\bar{\alpha }\right)\bar{S{W}^{\downarrow }}+\left(1-\bar{\alpha }\right){S{W}^{\downarrow }}^{{\prime} }-{\alpha }^{{\prime} }S{W}^{\downarrow }$. As such, Eq. (4) becomes:

5 $${{{\rm{\sigma }}}}{\bar{T}}^{4}+4{\bar{T}}^{3}{T}^{{\prime} }=\left[\left(1-\bar{\alpha }\right)\bar{S{W}^{\downarrow }}+\left(1-\bar{\alpha }\right){S{W}^{\downarrow }}^{{\prime} }-{\alpha }^{{\prime} }S{W}^{\downarrow }\right]+\\ \left(\bar{L{W}^{\downarrow }}+{{{LW}}^{\downarrow }}^{{\prime} }\right)-({\bar{Q}+Q}^{{\prime} })-\left[\bar{({LH}+{SH})}{+\left({LH}+{SH}\right)}^{{\prime} }\right]$$

By removing the mean-state equation from Eq. (5), we are left with:

6 $$4{\bar{T}}^{3}{T}^{{\prime} }=\left[\left(1-\bar{\alpha }\right){S{W}^{\downarrow }}^{{\prime} }-{\alpha }^{{\prime} }S{W}^{\downarrow }\right]+{{{LW}}^{\downarrow }}^{{\prime} }-{Q}^{{\prime} }-{\left({LH}+{SH}\right)}^{{\prime} }.$$

Further consider that the surface downward radiation anomaly has one component due to cloud radiative effects and another expected from a clear-sky condition, written as ${(\cdot )}^{\downarrow }={(\cdot )}^{{\downarrow},{cloud}}+{(\cdot )}^{{\downarrow},{clear}-{sky}}$. Hence, $\left(1-\bar{\alpha }\right){S{W}^{\downarrow }}^{\prime}$ = $\left(1-\bar{{{{\rm{\alpha }}}}}\right){{{{{\rm{SW}}}}}^{{\downarrow},{{{\rm{cloud}}}}}}^{\prime}+\left(1-\bar{{{{\rm{\alpha }}}}}\right){{{{{\rm{SW}}}}}^{{\downarrow},{{{\rm{clear}}}}-{{{\rm{sky}}}}}}^{\prime}$ and ${{{LW}}^{\downarrow }}^{\prime}={{{{{\rm{LW}}}}}^{{\downarrow},{{{\rm{cloud}}}}}}^{\prime}+{{{{{\rm{LW}}}}}^{{\downarrow},{{{\rm{clear}}}}-{{{\rm{sky}}}}}}^{\prime}$. Plug these two expressions and Eq. (2) into Eq. (6), rearranging terms, and dividing the equation by $4{\bar{T}}^{3}$, and ${T}^{\prime}$ can be decomposed as:

7 $${T}^{\prime}\,=	\frac{1}{4\sigma {\bar{T}}^{3}}\left[-{\alpha }^{\prime}{{SW}}^{\downarrow }+\underbrace{\left(1-\bar{{{{\rm{\alpha }}}}}\right){{{{{\rm{SW}}}}}^{{\downarrow},{{{\rm{cloud}}}}}}^{\prime}+{{{{{\rm{LW}}}}}^{{\downarrow},{{{\rm{cloud}}}}}}^{\prime}}_{{Cloud}\,{Radiative}\,{Effect}} \right. \\ 	\left.+\underbrace{\left(1-\bar{{{{\rm{\alpha }}}}}\right){{{{{\rm{SW}}}}}^{\downarrow,{{{\rm{clear}}}}-{{{\rm{sky}}}}}}^{\prime}+{{{{{\rm{LW}}}}}^{\downarrow,{{{\rm{clear}}}}-{{{\rm{sky}}}}}}^{\prime}}_{{clear}-{sky}\,{Radiation}}+\underbrace{{{\left(-\frac{\partial {OHC}}{\partial t}-{{{\boldsymbol{\nabla }}}}\cdot {{{\bf{OHT}}}}\right)}^{\prime}-\left({LH}+{SH}\right)}^{\prime}}_{{Oceanic}}\right]$$

Here, the SST anomaly (${T}^{\prime}$) is decomposed into seven components due to different physical processes corresponding to each term in the right hand side of Eq. (7): (1) surface albedo feedback (SAF; negligible in ice-free regions); (2) shortwave and (3) longwave cloud radiative effects; (4) surface clear-sky downward shortwave irradiance; (5) surface clear-sky downward longwave irradiance; (6) ocean heat storage and OHT divergence (estimated as residual); and (7) surface turbulent heat fluxes. Specifically, for global means, ${\left({{{\boldsymbol{\nabla }}}}\cdot {{{\bf{OHT}}}}\right)}^{\prime}=0$, and Term (6) in Eq. (7) reduces to the heat storage anomaly; while for a local domain, such as the defined subpolar North Atlantic region, $\left|{\left(\partial {OHC}/\partial t\right)}^{\prime}\right|\ll \left|{\left({{{\boldsymbol{\nabla }}}}\cdot {{{\bf{OHT}}}}\right)}^{\prime}\right|$, and Term (6) mainly represents the OHT convergence anomaly. The difference between the subpolar North Atlantic mean ${T}^{\prime}$ and global mean ${T}^{\prime}$ hence corresponds to the decomposed WHSST anomalies.

## Supplementary information

## Data Availability

The CESM2 Large Ensemble datasets are available at https://www.cesm.ucar.edu/community-projects/lens2, and the single forcing ensemble datasets are available at https://www.cesm.ucar.edu/working-groups/climate/simulations/cesm2-single-forcing-le. Data output from the models participating in CMIP6 are available via the Earth System Grid Federation (ESGF) servers, with information on obtaining data available at https://pcmdi.llnl.gov/CMIP6/Guide/dataUsers.html. Data from the North Atlantic hosing experiments are available at https://doi.org/10.5281/zenodo.7643437 (Jackson et al. 2023). Convenient source data for reproducing the main figures are available at https://doi.org/10.5281/zenodo.20615811.

## Code availability

The Python Jupyter Notebooks used for detecting change points, decomposing SST anomalies, and plotting main figures are archived in https://github.com/fay1322/Fan26-AMOC-SST-Regime.git.
