# Supplementary material for: Regime shifts of AMOC-sea surface temperature relationship
Source: Nat Commun. 2026 Aug 5;17:9416. doi: 10.1038/s41467-026-76149-4 (PMC13538684; doi:10.1038/s41467-026-76149-4)
Supplement: Supplementary file 1 [file 41467_2026_76149_MOESM1_ESM.pdf]

Supplementary Information

Regime shifts of AMOC-sea surface temperature relationship

Yifei Fan[1], Duo Chan[2*], Gokhan Danabasoglu[3], Who M. Kim[3], Pengfei Zhang[4*], Laifang Li[1, 5, 6*]

[1]Department of Meteorology and Atmospheric Science, Pennsylvania State University, University Park, PA, USA

[2]School of Ocean and Earth Science, University of Southampton, Southampton, Hampshire, UK

[3]National Science Foundation National Center for Atmospheric Research, Boulder, CO, USA

[4]College of Oceanic and Atmospheric Science, Ocean University of China, Qingdao, China

[5]Institute of Computational and Data Sciences, Pennsylvania State University, University Park, PA, USA

[6]Earth and Environmental Systems Institute, Pennsylvania State University, University Park, PA, USA

Table of Content

SUPPLEMENTARY FIGURES 3

Fig. S1 3
Fig. S2 4
Fig. S3 5
Fig. S4 6
Fig. S5 7
Fig. S6 9
Fig. S7 10
Fig. S9 12
Fig. S10 13
Fig. S11 14
Fig. S12 15
Fig. S13 16
Fig. S14 17
Fig. S15 18
Fig. S16 19
Fig. S17 20

SUPPLEMENTARY TABLES 21

Supplementary Table 1 21
Supplementary Table 2 22
Supplementary Table 3 23
Supplementary Table 4 24

SUPPLEMENTARY INFORMATION 25

Initial knowledge used in the genetic algorithm 25
Uncertainty in change point detection 25

Supplementary Figures

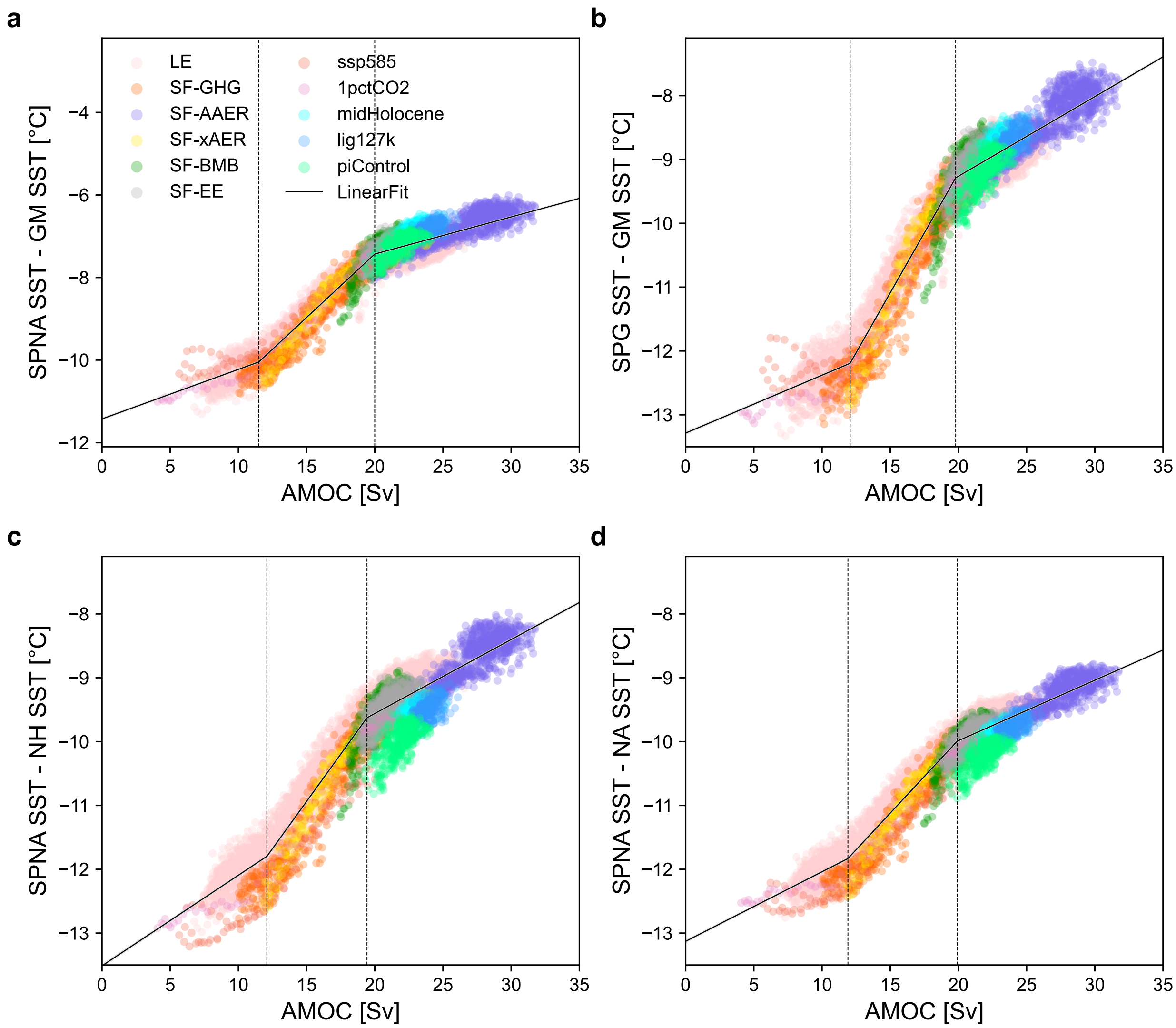

Fig. S1: Robustness against different sea surface temperature (SST) metrics. a–d, AMOC versus the difference between (**a**) subpolar North Atlantic (SPNA) SST and global mean (GM) SST, that is, the warming hole SST (WHSST; same as Fig. 1a), (**b**) subpolar gyre (SPG) SST and global mean (GM)–same as the one used in Caesar et al. (2018), (**c**) SPNA SST and Northern Hemisphere (NH) SST, and (**d**) SPNA SST and North Atlantic (NA; 0°–60°N, 90°W–0°E) SST. The vertical dotted lines represent two change points detected for each SST metric separately. They are 11.5 Sv and 12.0 Sv in (a; the ones adapted in the manuscript), 12.06 Sv and 19.80 Sv in (b), 12.09 Sv and 19.43 Sv in (c), and 11.90 Sv and 19.91 Sv in (d). Black solid lines indicate the least squared regression with the corresponding change points.

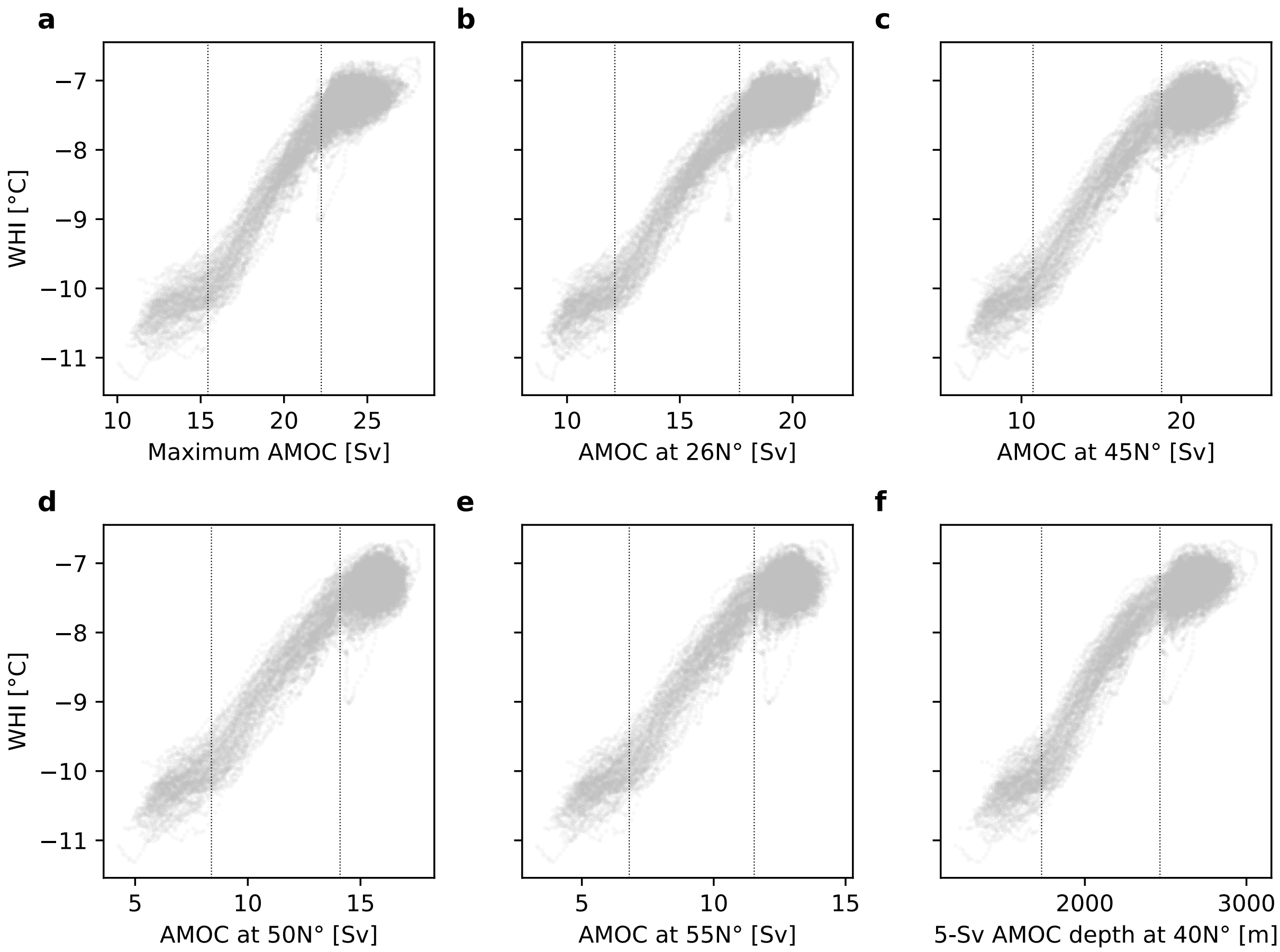

Fig. S2: Robustness against different AMOC metrics. a, Maximum AMOC across all Northern Hemisphere latitudes (below 500 meters). **b**–**e**, Maximum AMOC at 26°N, 45°N, 50°N, and 55°N, respectively. **f**, The depth of the 5-Sv isoline of volume transport in the upper cell at 40°N. All data are from CESM2-LE simulations and smoothed by a 15-year moving window. Vertical lines denote two corresponding change points, calculated from linear regression between the alternative AMOC metric and the AMOC index used in the manuscript (maximum AMOC at 40°N).

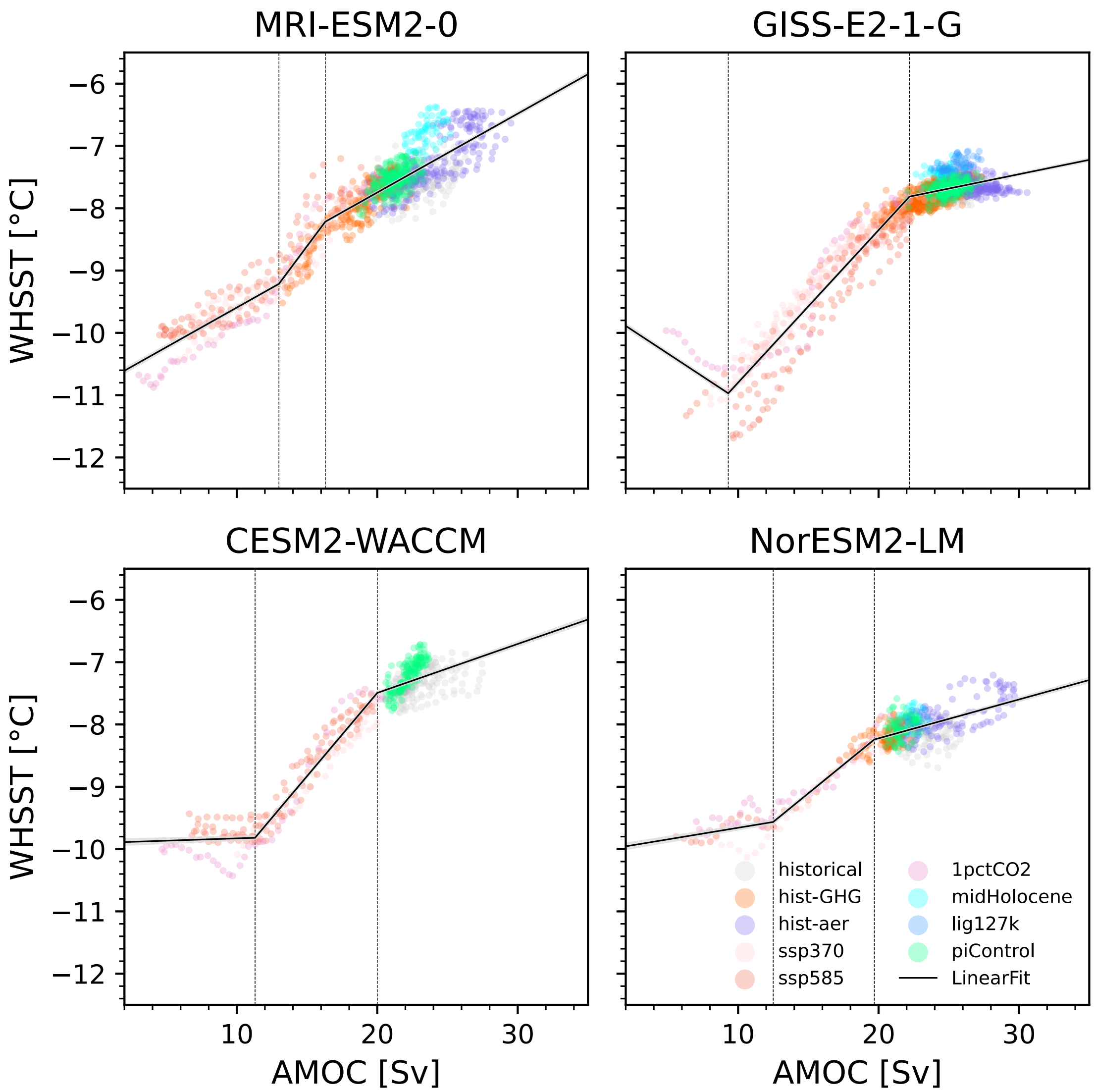

Fig. S3: The AMOC-WHSST relationship in four different compilations of CMIP6 simulations. The annual mean AMOC index (unit: Sv) versus the annual mean warming hole SST (unit: °C) in the compilation of simulations with MRI-ESM2-0, GISS-E2-1-G, CESM2-WACCM, and NorESM2-LM (see Methods and Supplementary Table 1). All data are smoothed by a 15-year moving window. One dot is plotted every three years for visualization. The two vertical lines denote the two identified change points for each model, as shown in Fig. 1b. Black solid lines indicate the least squared regression with the change points.

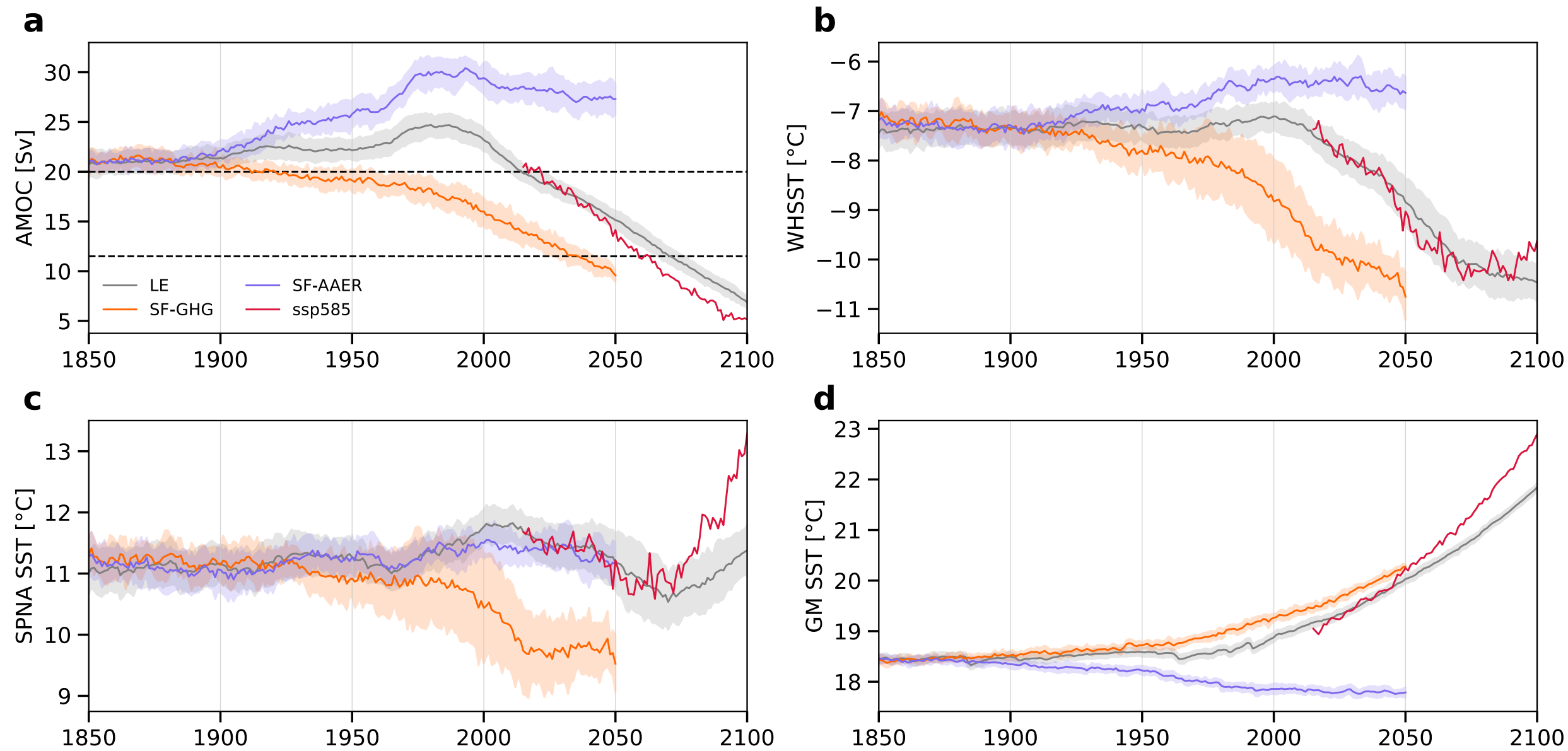

Fig. S4: The AMOC and SST time series in the CESM2 simulations. a, The AMOC index in the full-forcing CESM2-LE, the greenhouse gas forcing only ensemble (SF-GHG), the anthropogenic aerosol forcing only ensemble (SF-AAER), and the *SSP5-8.5* simulation. The thick lines are ensemble means, and the shaded area denotes one standard deviation across the ensemble. **b–d**, Same as (a) except that it is for the WHSST (b), the subpolar North Atlantic regional-mean SST (c), and the area-weighted global-mean SST (d).

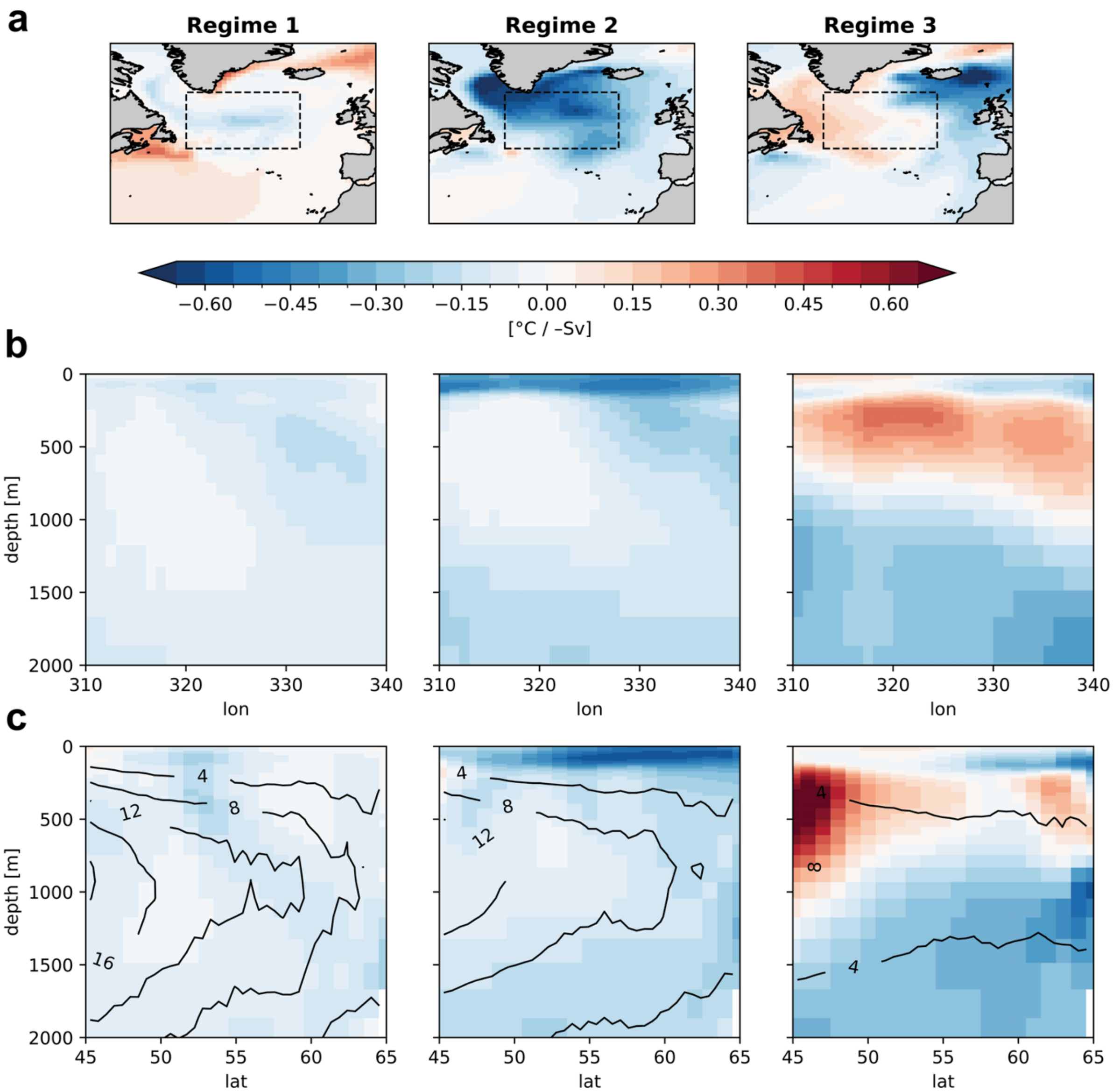

Fig. S5: Changes in ocean potential temperatures. a, The ensemble-mean difference in trends in ocean potential temperature at the 5-m depth and global mean SST over 1985–2014 (Regime 1), 2015–2070 (Regime 2), and 2071–2100 (Regime 3) in the CESM2-LE simulations. **b**, Same as (a) but for the zonal section of the meridional mean ocean temperatures in the subpolar North Atlantic domain (denoted by the box in (a)). **c**, Same as (b) but for the meridional section of the zonal mean ocean temperatures in the subpolar North Atlantic. Al trends are scaled by the rate of AMOC weakening over the corresponding period. The contours in (c) denotes the Atlantic overturning stream function climatology over the three periods and are shown in a 4-Sv interval.

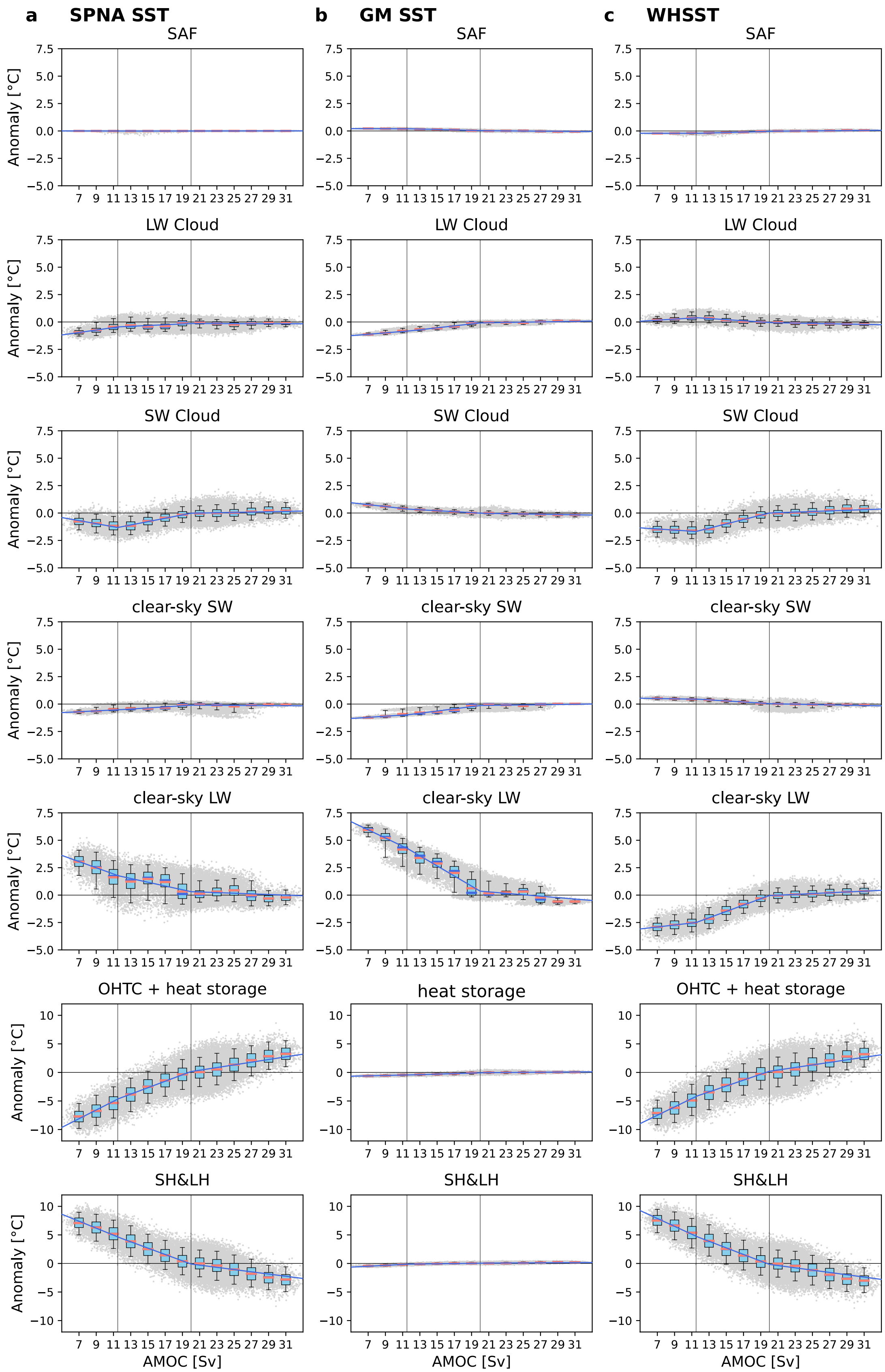

a SPNA SST
b GM SST
c WHSST
SAF
LW Cloud
SW Cloud
clear-sky SW
clear-sky LW
OHTC + heat storage
heat storage
SH&LH
Anomaly [°C]
AMOC [Sv]

Fig. S6: AMOC versus each term of decomposed SST anomalies in CESM2 simulations. a, The AMOC index versus decomposed subpolar North Atlantic SST anomalies. From left to right are SST anomalies due to (1) surface albedo feedback (SAF), (2) longwave cloud radiative effect (LW Cloud), (3) shortwave cloud radiative effect (SW Cloud), (4) surface downward clear-sky shortwave radiation (clear-sky SW) and (5) longwave radiation (clear-sky LW), (6) ocean heat transport convergence and heat storage (OHTC + heat storage), and (7) surface sensible heat and latent heat fluxes (SH&LH). Each year is plotted as a gray dot, and the boxplots denote the statistics of WHI anomalies corresponding to a 2-Sv bin of the AMOC index (box, interquartile range; whiskers, 90% range; pink line, mean; blue line, median). The two vertical lines separate the three AMOC regimes, and the blue line inside each regime indicates the linear fit between the AMOC and the SST anomalies. **b-c**, Similar to (a) but for (**b**) AMOC index versus the area-weighted global mean SST anomalies (excluding regions at latitudes higher than 70°) and (**c**) the WHSST anomalies. Data are from the 100-member full-forcing ensemble (LE) and four single-forcing ensembles (SF-AAER, SF-GHG, SF-BMB, and SF-EE).

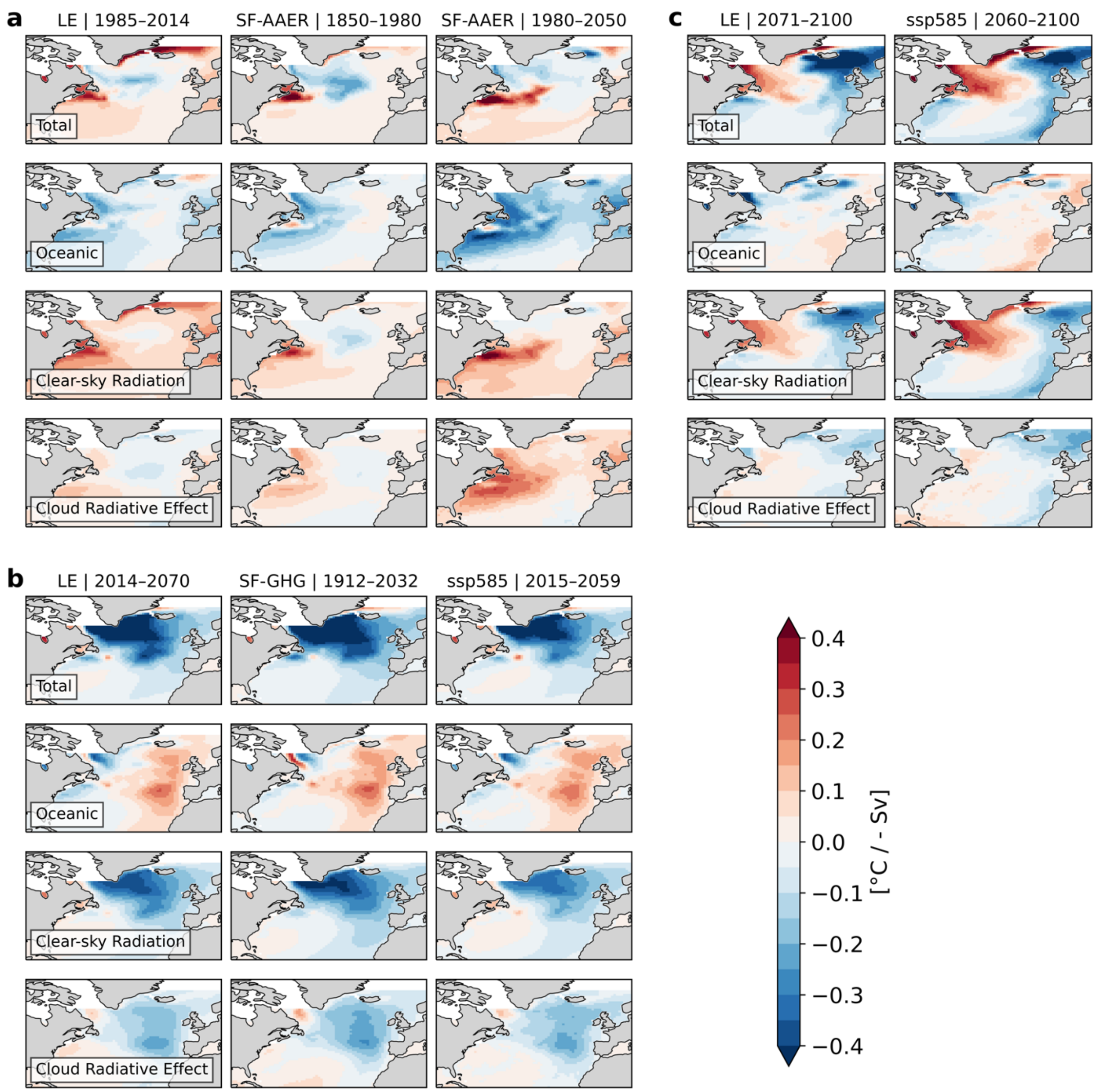

Fig. S7: Decomposed SST trends in these regimes in multiple forcing scenarios. a, From left to right are ensemble-mean SST trends minus area-weighted global-mean SST over the time periods that correspond to Regime 1 in the LE and SF-AAER simulations. From top to bottom are the total SST trend and the SST trend due to oceanic processes, clear-sky radiation, and cloud radiative forcing. **b**, Same as (a) except that it shows SST trends over the time periods that correspond to Regime 2 in the LE, SF-GHG, and SSP5-8.5 simulations. **c**, Same as (a) except that it shows SST trends over time periods that correspond to Regime 3 in the LE and SSP5-8.5 simulations. The SST trends are scaled by the AMOC trends over the same period multiplied by –1, thus having a unit of °C per –1 Sv.

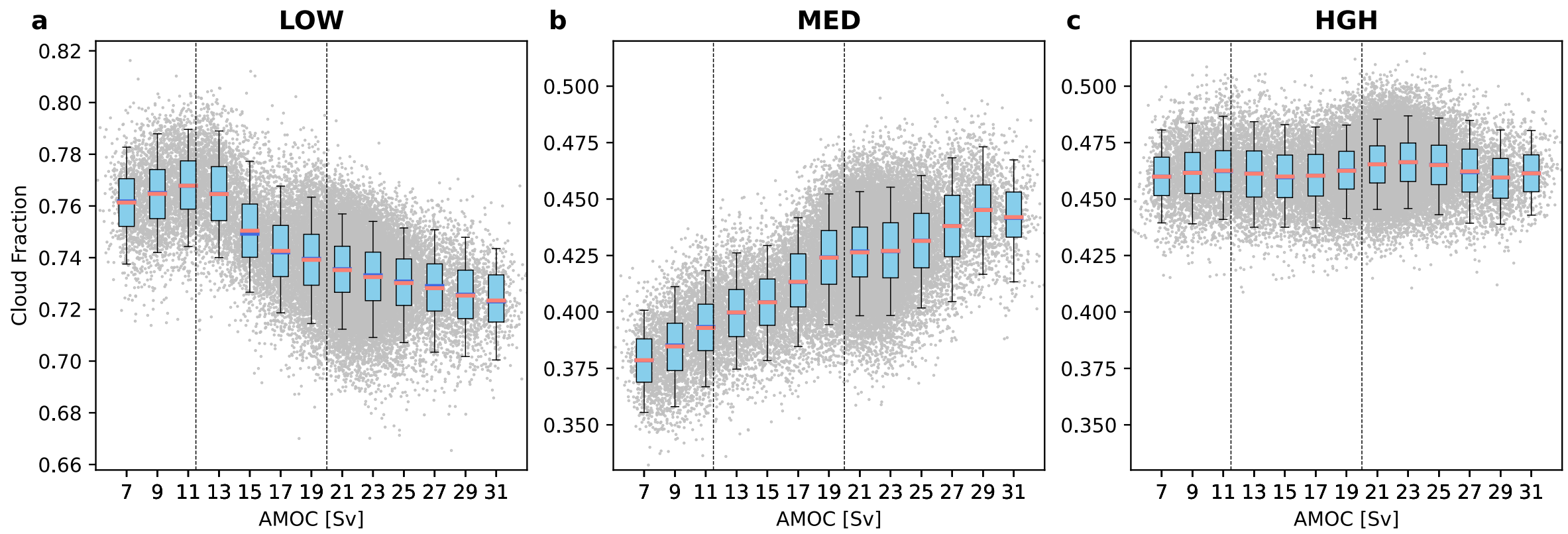

Fig. S8: AMOC versus subpolar North Atlantic cloud fractions in the CESM2 simulations. From left to right are the AMOC index versus subpolar North Atlantic regional-mean cloud fraction for low-level, intermediate-level, and high-level clouds, similar to Fig. 2f. Each year is plotted as a gray dot.

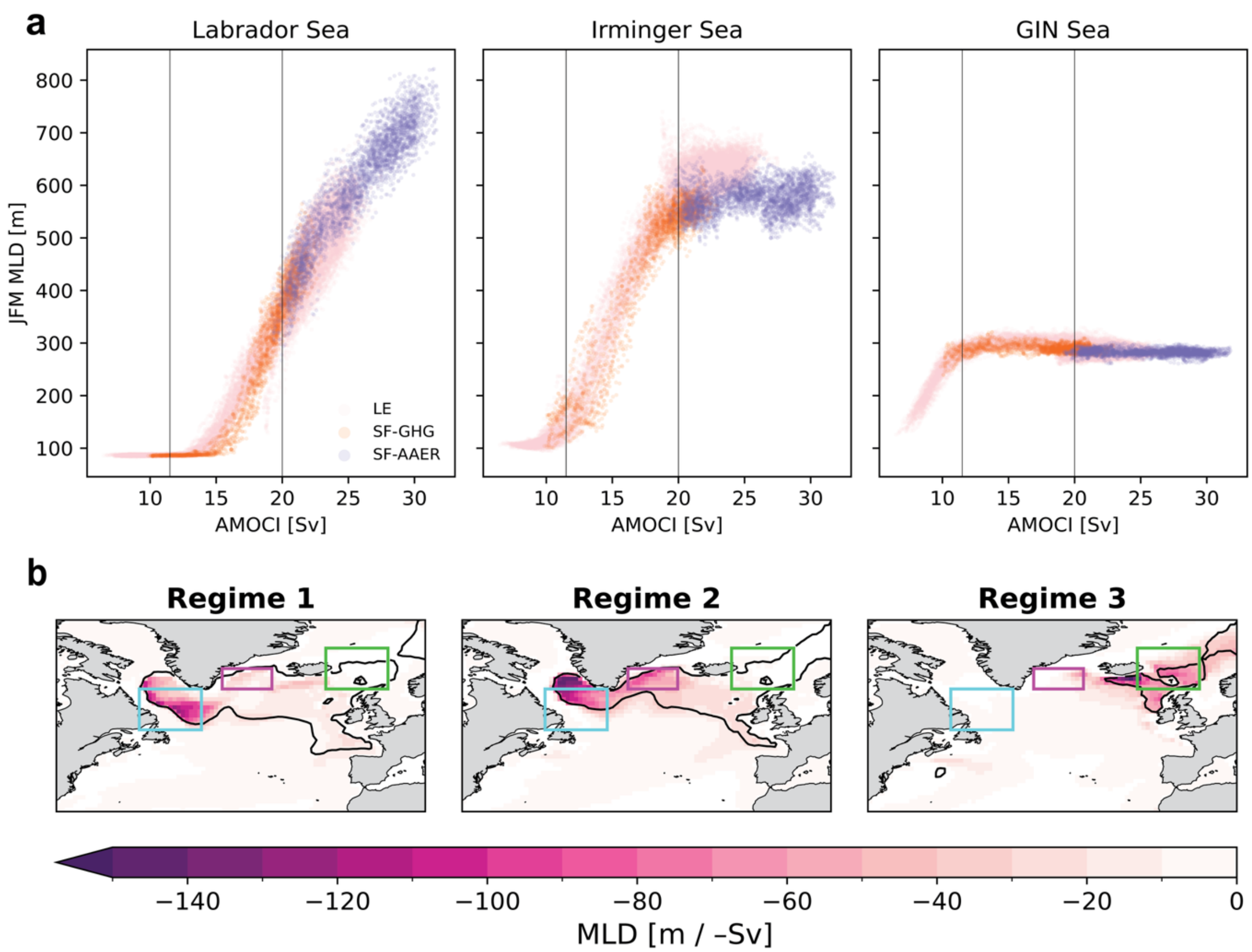

Fig. S9: **January-February-March (JFM) mixed layer depth (MLD) change with AMOC. a**, AMOC versus JFM MLD averaged over the Labrador Sea (50°–60°N, 40°–60°W), the Irminger Sea (60°–65°N, 28°–40°W), and the Greenland-Iceland-Norwegian Sea (60°–70°N, 0°–20°W), which are shown by the cyan, magenta, yellow boxes in (b), respectively. All data are smoothed with a 15-year window. **b**, CESM2-LE mean JFM MLD trends scaled by AMOC trends over the 1985–2014, 2015–2070, and 2071–2100 periods, corresponding to Regime 1, 2, and 3, respectively. Black contours in (b) represent the temporal mean JFM MLD isoline of 250 m.

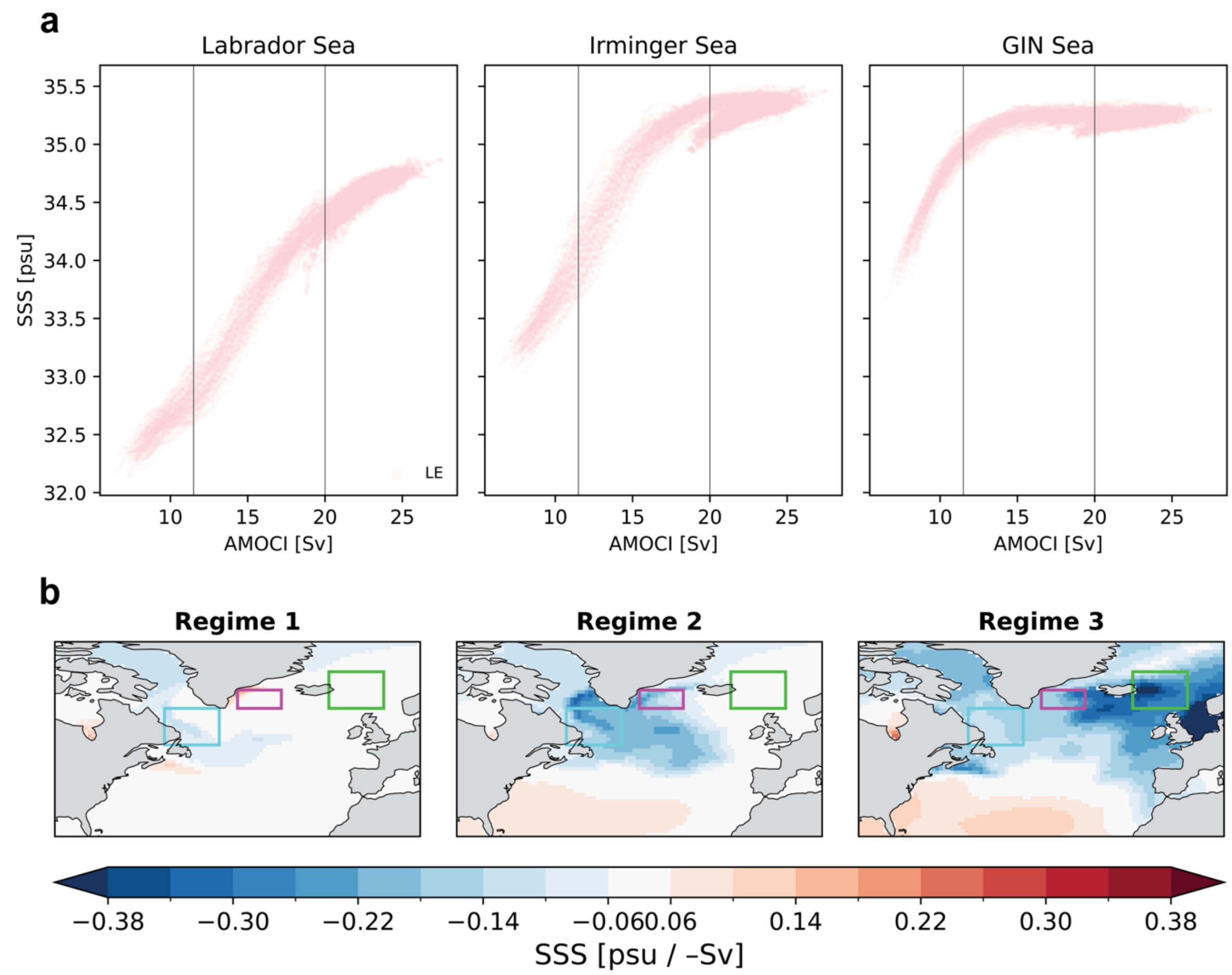

Fig. S10: Salinity change with AMOC. **a**, AMOC versus JFM sea surface salinity (SSS) averaged over the Labrador Sea, the Irminger Sea, and the Greenland-Iceland-Norwegian Sea. All data are smoothed with a 15-year window in CESM2-LE. **b**, CESM2-LE ensemble mean JFM SSS trends scaled by AMOC trends over the 1985–2014, 2015–2070, and 2071–2100 periods, corresponding to Regime 1, 2, and 3, respectively. Dark navy contours in (b) represent the temporal mean ice fraction area isoline of 0.50.

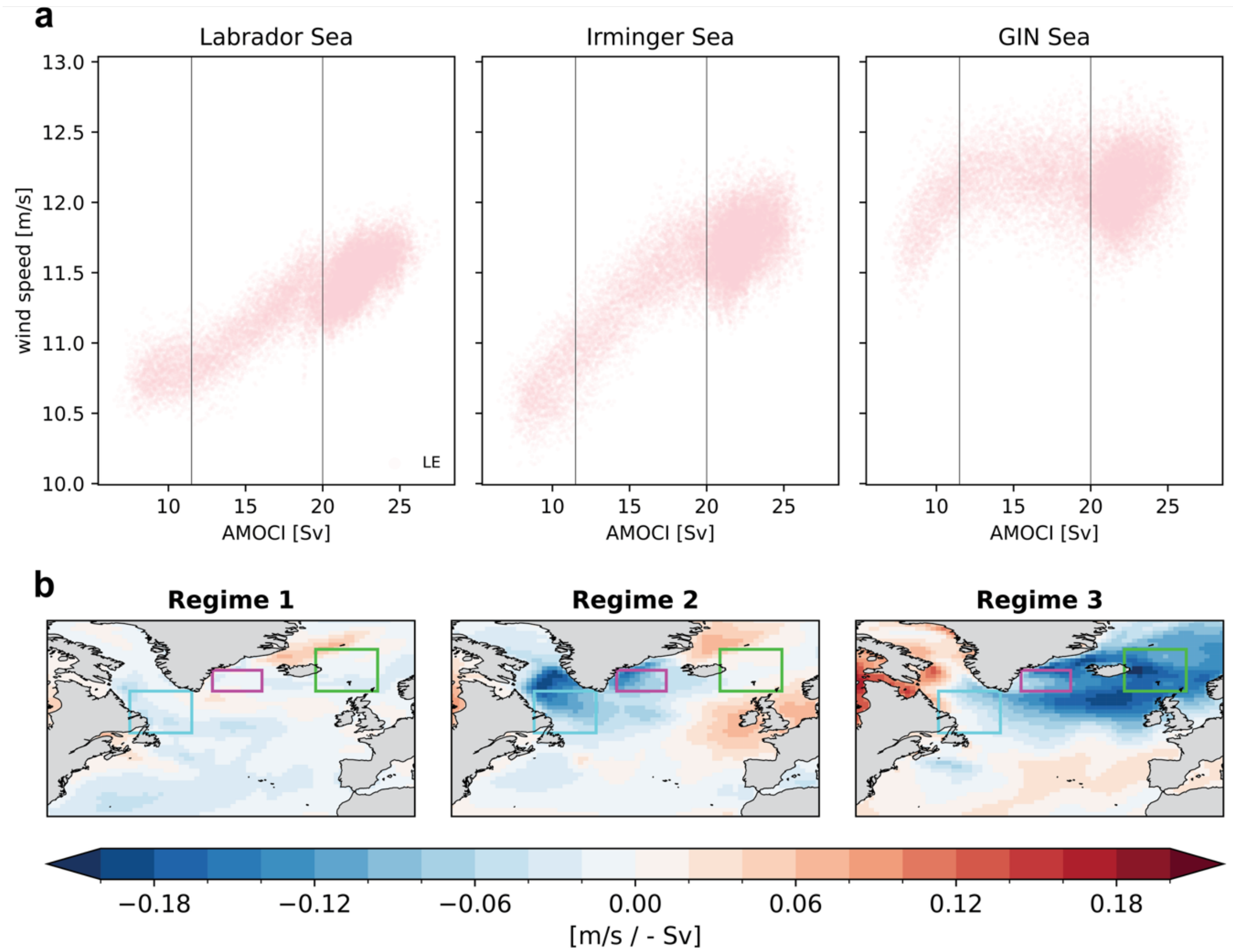

Fig. S11: Wind speed change with AMOC. Same as Fig. S10 but for surface wind speed.

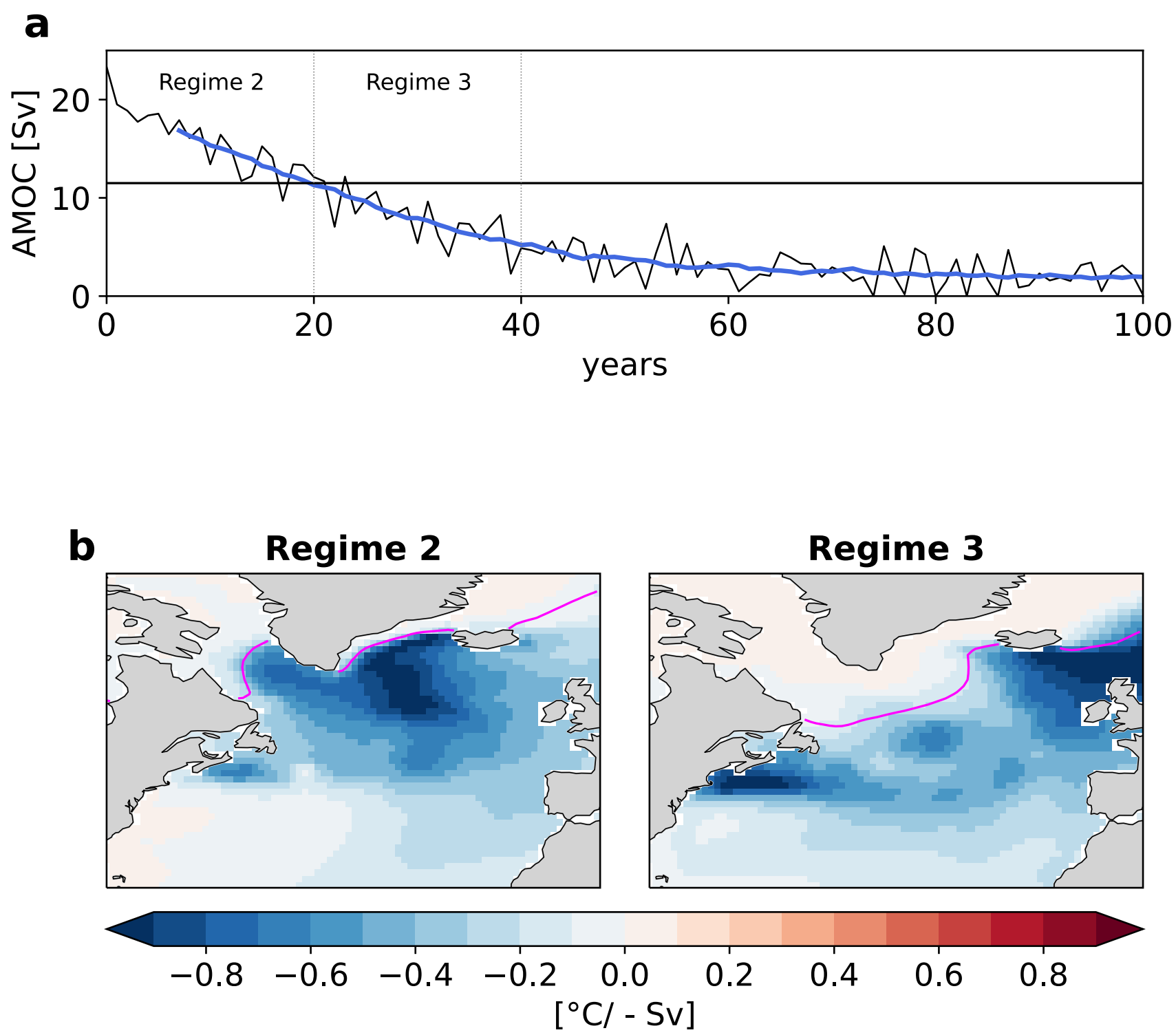

Fig. S12: AMOC and relevant North Atlantic changes in a hosing simulation. **a**, The annual-mean (black) and 15-year smoothed (blue) AMOC index in CESM2's u03-hos simulation. The horizontal line denoted the previously detected Change Point 2 in the AMOC-WHSST relationship (11.5 Sv), and the two vertical dotted lines separate the time periods corresponding to Regimes 2 and 3. **b**, The SST trend minus global mean SST trend, scaled by the AMOC trend, in the two regimes. The magenta contour denotes the zero values of temporal mean SST in the two periods.

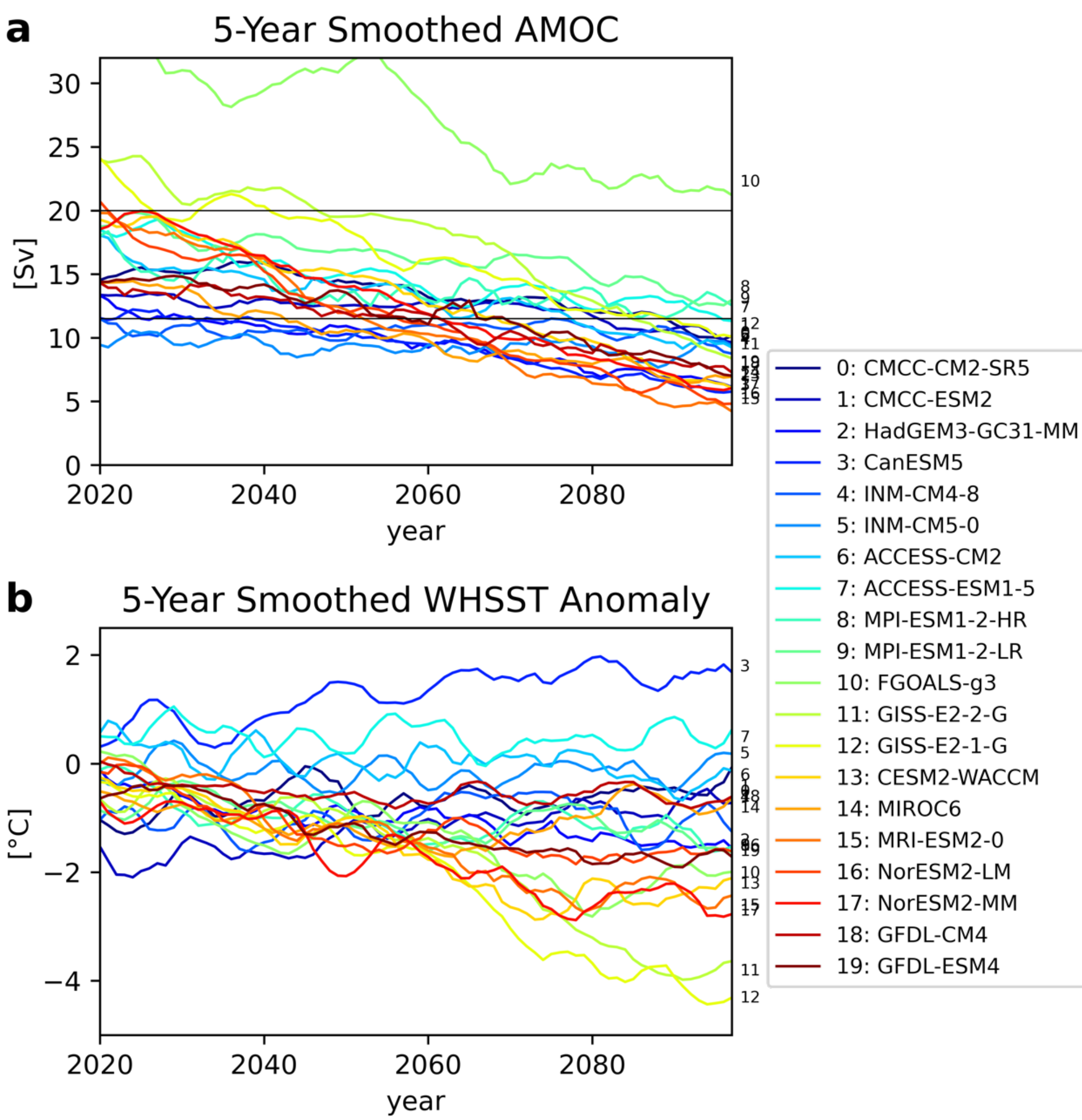

Fig. S13: **CMIP6 model selection.** The 5-year smoothed time series of (**a**) the AMOC index and (**b**) the WHSST anomalies (referenced to the 1850–1880 climatology) in the SSP5-8.5 simulations from 20 models participating in CMIP6. Note that AMOC in FGOALS-g3 is too strong and never reaches the two change points (20 Sv and 11.5 Sv). In addition, the WHSST does not show any decreasing trend despite AMOC weakening in ACCESS-ESM1-5, ACCESS-CM2, CanESM5, INM-CM4-8, and INM-CM5-0. We hence exclude these models when examining the 21-st century evolution of AMOC-WHSST relationship across regimes in the main text and Fig. 5. FGOALS-g3 is excluded because the simulated AMOC is anomalously strong compared to other models. Information on the realization used here is summarized in Supplementary Table 3.

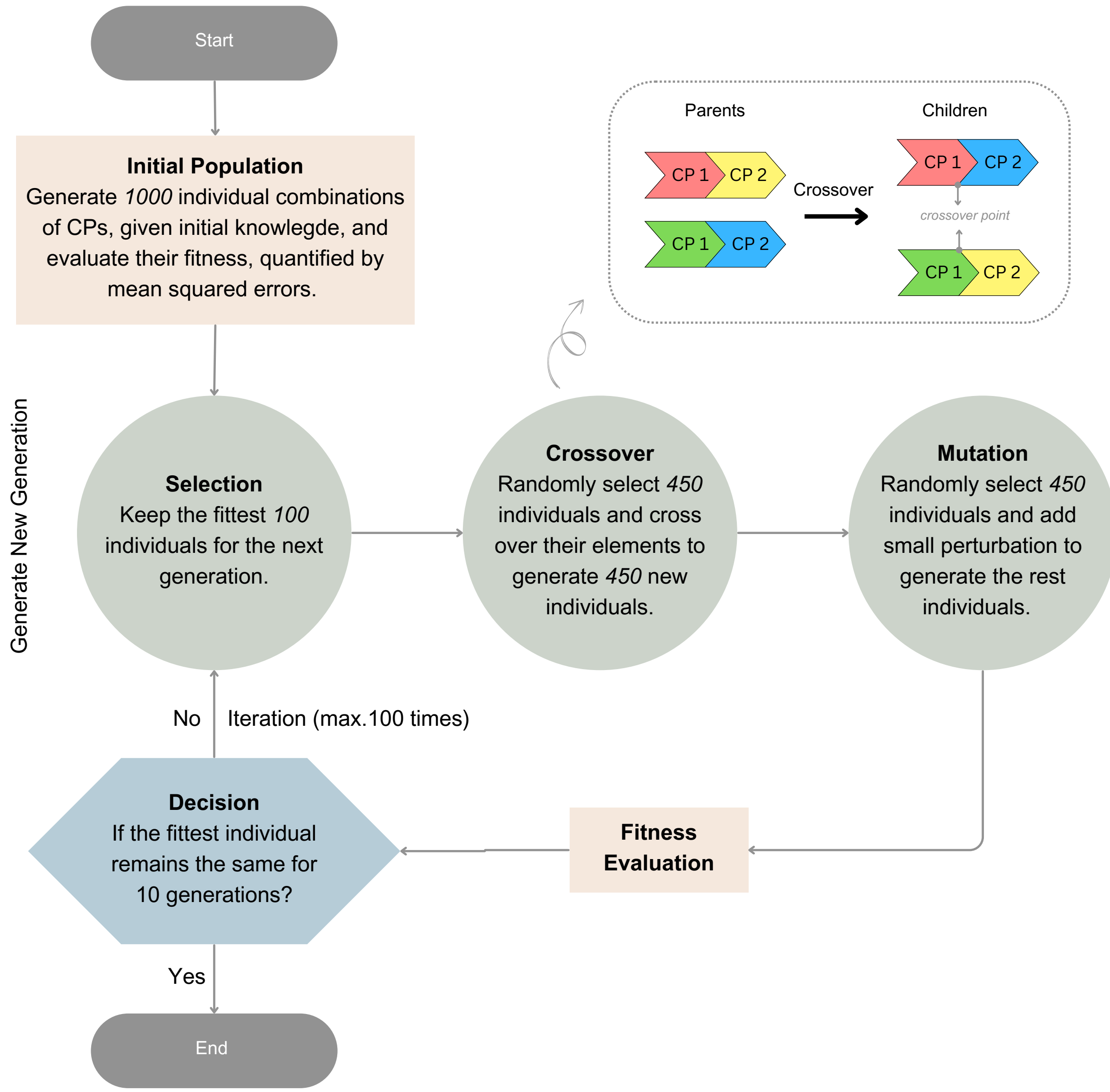

Fig. S14: **The workflow of detecting change points in the AMOC-WHSST relationship using a genetic algorithm.**

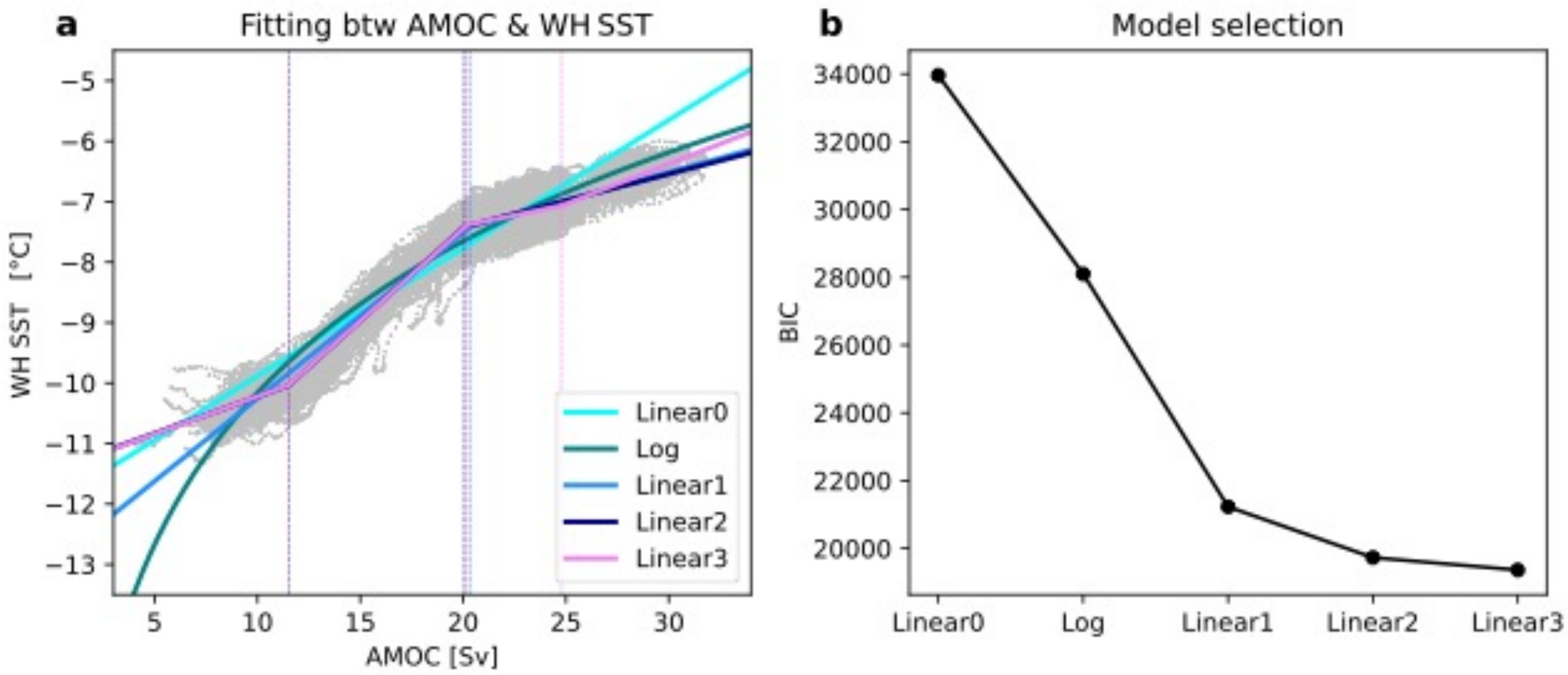

Fig. S15: **Evaluation of five statistical models**. **a**, Statistical fitting between the AMOC index and the WHSST in a compilation of CESM2 simulations, as shown in Fig. 1a. The dotted vertical lines denote the change points of the corresponding linear fitting, as summarized in Supplementary Table 4. **b**, Bayesian information criteria (BIC) of each statistical fitting models in (a).

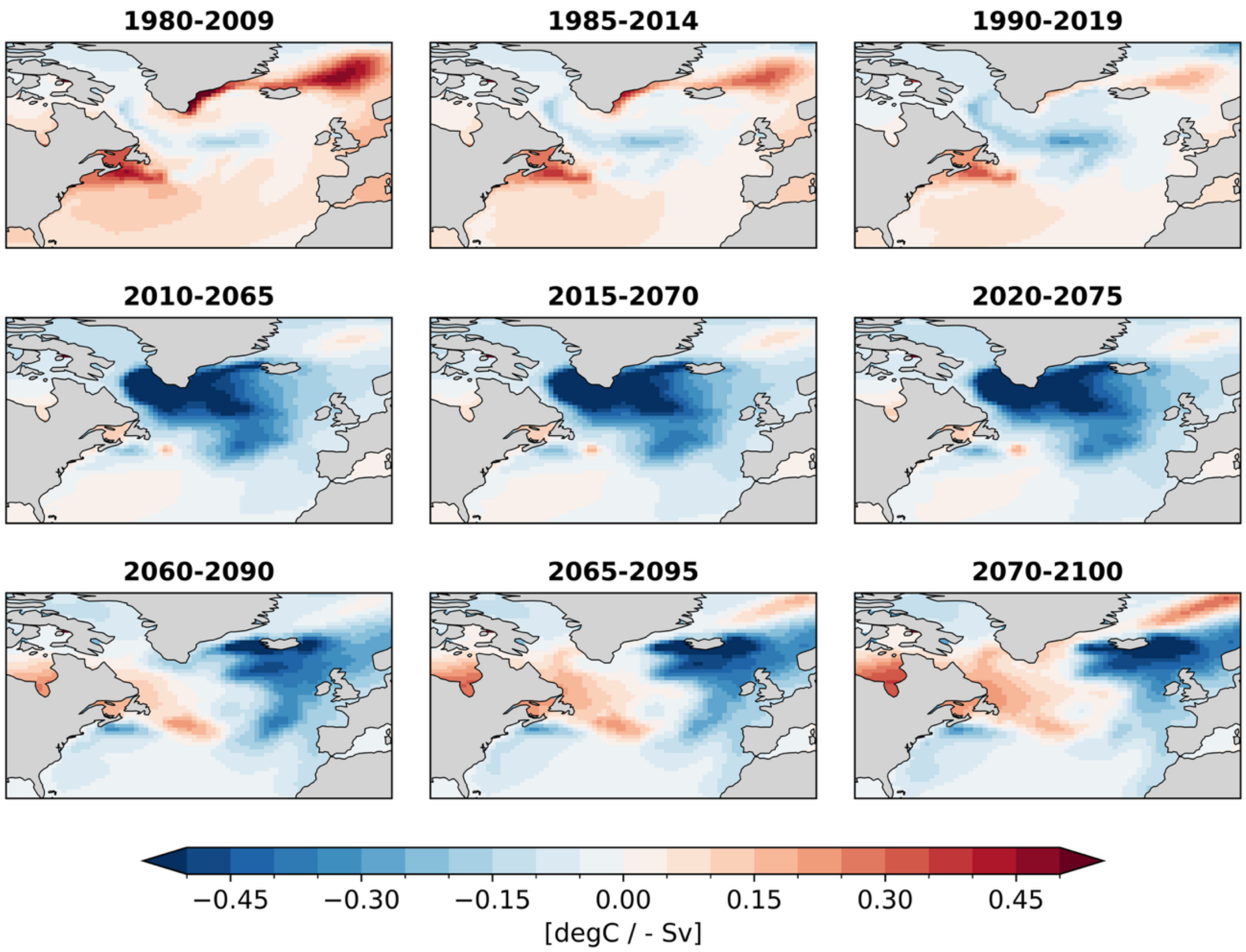

Fig. S16: **CESM2-LE mean SST trends (with global mean removed) scaled by AMOC trends over different time periods.** The top, middle, and bottom rows correspond to the patterns of SST sensitivity to AMOC weakening in Regime 1, 2, and 3, as shown in Fig. 1d.

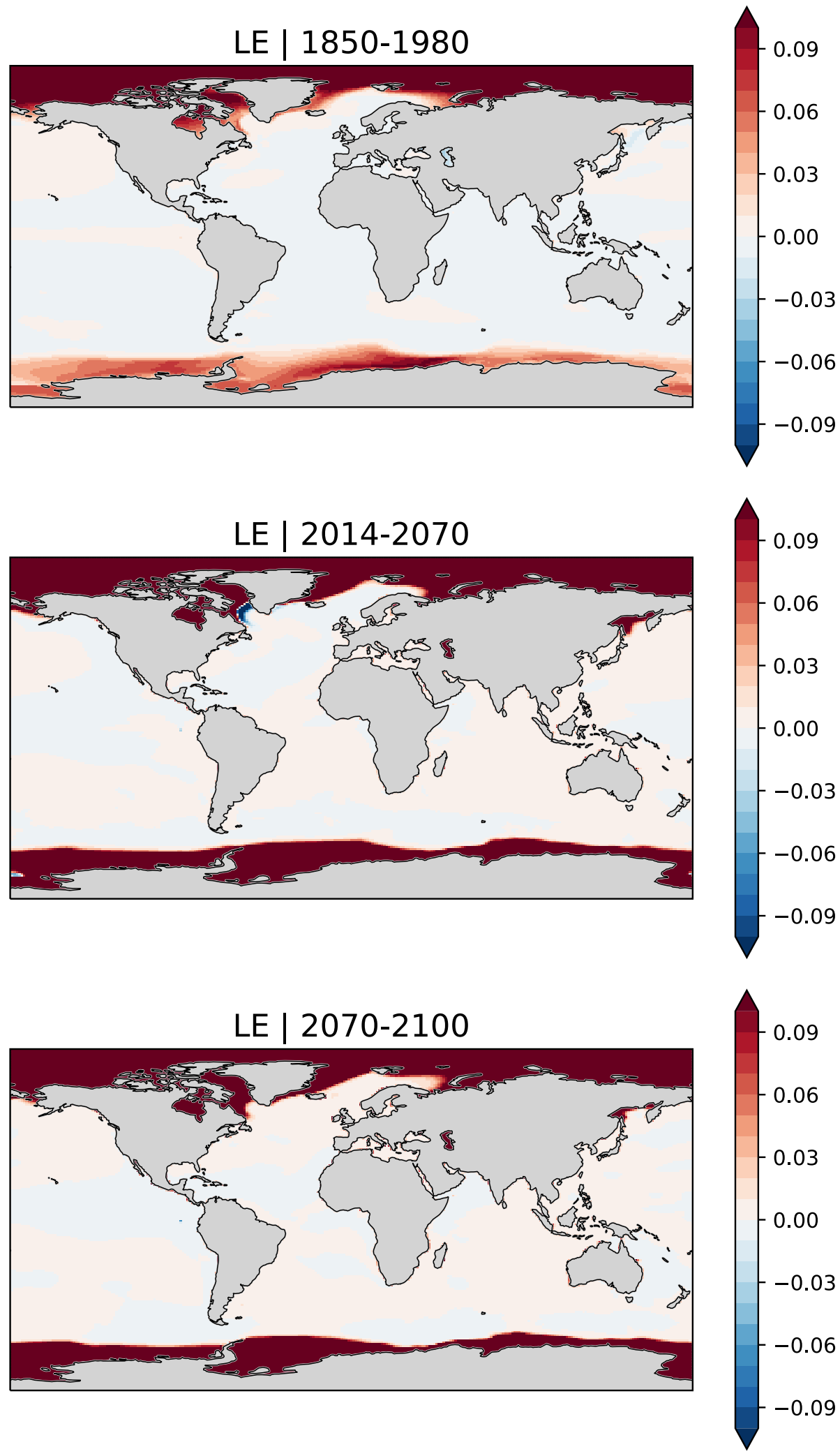

Fig. S17: **Applicability of the SST change decomposition framework.** The ensemble-mean differences between the sum of decomposed temperature trends and the simulated SST trends over the time periods that correspond to the three regimes in the CESM2-LE simulations (unit: °C per decade).

Supplementary Tables

Supplementary Table 1: **CESM2 simulations analyzed in the study.**

Experiment	Description
**LE**	A 100-member ensemble from 1850–2100 under CMIP6 historical (1850–2014) and SSP370 (2015–2100) forcing
**SF-GHG**	A 15-member ensemble from 1850–2050 under CMIP6 historical (1850–2014) and SSP370 (2015–2050) forcing but with only greenhouse gas (GHG) evolving with time
**SF-AAER**	A 20-member ensemble from 1850–2050 but with only anthropogenic aerosols (AAER, including industrial, agricultural, domestic and transport related emissions) evolving with time
**SF-xAER**	A 10-member ensemble from 1920–2050 with all-but-AAER evolving with time
**SF-BMB**	A 15-member ensemble from 1850–2050 with only biomass burning (BMB) aerosols evolving with time
**SF-EE**	A 15-member ensemble from 1850–2050 with all other forcings evolving with time except GHG, AAER and BMB
SSP5-8.5	A 3-member ensemble from 2015–2100 under CMIP6 SSP585 forcing scenario
1pctCO2	A 150-year simulation where the atmospheric CO_2 concentration is gradually increased at a rate of 1%/year from the global annual-mean 1850 value until quadrupling
midHolocene	A 700-year paleoclimate simulation of the mid-Holocene (6k years before present)
lig127k	A 700-year paleoclimate simulation of the Last Interglacial (127k years before present)
piControl	A 1200-year control simulation with non-evolving pre-industrial conditions
u03-hos	A 172-year simulation with a uniform freshwater input of 0.3 Sv north of 50°N and all external forcings fixed at 1850s conditions.

Supplementary Table 2: **Simulations shown in Fig. 1 and their corresponding number of ensembles and time periods.** (*For these GISS-E2-1-G simulations, runs with ‘f2’ are used.)

<table>
<tr><th></th><th>MRI-ESM2-0</th><th>CESM2-WACCM</th><th>GISS-E2-1-G</th><th>NorESM2-LM</th></tr>
<tr><td>historical</td><td rowspan="3">5 runs; 1850–2014</td><td>3 runs; 1850–2014</td><td>10 runs; 1850–2014</td><td rowspan="3">3 runs; 1850–2014</td></tr>
<tr><td>hist-GHG</td><td rowspan="2">x</td><td>15 runs; 1850–2014</td></tr>
<tr><td>hist-aer</td><td>5 runs; 1850–2014</td></tr>
<tr><td>SSP3-7.0</td><td rowspan="2">5 runs; 2015–2100</td><td>3 runs; 2015–2100</td><td>10 runs*; 2015–2100</td><td rowspan="2">1 run; 2015–2500</td></tr>
<tr><td>SSP5-8.5</td><td>5 runs; 2015–2100</td><td>5 runs*; 2015–2500</td></tr>
<tr><td>1pctCO2</td><td colspan="4">1 run; 150 yrs</td></tr>
<tr><td>midHolocene</td><td>1 run; 200 yrs</td><td rowspan="2">x</td><td rowspan="2">1 run; 200 yrs</td><td rowspan="2">1 run; 100 yrs</td></tr>
<tr><td>lig127k</td><td>x</td></tr>
<tr><td>piControl</td><td>1 run; 701 yrs</td><td>1 run; 499 yrs</td><td>1 run; 851 yrs</td><td>1 run; 301 yrs</td></tr>
</table>

Supplementary Table 3: **Models and the identifiers of the realizations used for estimating future climate projections under *SSP5-8.5* forcing scenarios.** The asterisk denotes the models chosen for estimating the 21st-century evolution of the AMOC-WHSST relationship in the main text.

Model	Identifier
CESM2*	r4i1p1f1
CESM2-WACCM*	r1i1p1f1
CMCC-CM2-SR5*	
CMCC-ESM2*	
GFDL-ESM4*	
GFDL-CM4*	
GISS-E2-1-G*	r1i1p1f2
GISS-E2-2-G*	r1i1p3f1
HadGEM3-GC31-MM*	r1i1p1f3
MIROC6*	r1i1p1f1
MRI-ESM2-0*	
NorESM2-LM*	
NorESM2-MM*	
MPI-ESM1-2-HR*	
MPI-ESM1-2-LR*	
ACCESS-ESM1-5	
ACCESS-CM2	
CanESM5	
FGOASL-g3	
INM-CM4-8	
INM-CM5-0	

Supplementary Table 4: **The solved parameters of the statistical models used for fitting the AMOC-WHSST relationship in CESM2 simulations.** For those with change points, also shown is the initial knowledge about the change points for the genetic algorithm.

Statistical Model	Initial Knowledge	Solved Parameters
Linear, no change point	none	$b_0 = -12.0;\ b_1 = -0.212$
Logarithmic, no change point	none	$b_0 = -18.5;\ b_1 = 3.62$
Linear, 1 change point	$x_1 \in [10.7, 25.8]$ (the 5% and 95% quantiles)	$b_0 = -13.0;\ b_1 = 0.274;$ $b_2 = 0.0933;\ x_1 = 20.4$
Linear, 2 change points	$x_1 \in [5, 17]$ $x_2 \in [17, 30]$	$b_0 = -11.5;\ b_1 = 0.124;$ $b_2 = 0.309;\ b_3 = 0.0946;$ $x_1 = 11.59;\ x_2 = 19.87$
Linear, 3 change points	$x_1 \in [10, 13]$ $x_2 \in [19, 21]$ $x_3 \in [21, 30]$	$b_0 = -11.4; b_1 = 0.121;$ $b_2 = 0.309; b_3 = 0.0940;$ $b_4 = 0.135; x_1 = 11.5;$ $x_2 = 20.2; x_3 = 24.9$
Linear, 4 change points	$x_1 \in [5, 10]$ $x_2 \in [10, 13]$ $x_3 \in [19, 21]$ $x_4 \in [21, 30]$	$b_0 = -11.1; b_1 = 0.0767;$ $b_2 = 0.131; b_3 = 0.304;$ $b_4 = 0.0680; b_5 = 0.133;$ $x_1 = 7.57; x_2 = 11.6;$ $x_3 = 20.2; x_4 = 24.8$

Supplementary Information

Initial knowledge used in the genetic algorithm

A genetic algorithm was used for identifying change points (x_c) in the AMOC-WHSST relationship. The workflow is illustrated in Fig. S9. When generating initial populations of change points, we used some initial knowledge about the range of the change point. For example, for the linear model with one change point, x_1, the 5th percentile and the 95th percentile of the AMOC index were used as the lower and upper bound to randomly generate the initial values of x_{c1}; and for the linear model with two change points, $x_1$and x_2, we assumed $x_1 \in [5, x_c]$ and $x_2 \in [x_c, 30]$, where x_c was the critical value and set to be 17 (Supplementary Table 4). We also tested the sensitivity of the detected change points to the initial knowledge (i.e., the value of x_c) and found the results insensitive.

Uncertainty in change point detection

The detection of change points is constrained by sample size (i.e., ensemble size for each forcing scenario). The four CMIP6 models analyzed here have relatively small ensembles (Supplementary Table 2), making it difficult to directly quantify uncertainty in the identified change points and thereby complicating inter-model comparisons. To address this, we use the CESM2 ensembles to indirectly estimate the effect of limited sample size. Specifically, we randomly subsample three members (N = 3) from CESM2 LE and SF simulations 1000 times to match the ensemble sizes of the CMIP6 models and treat the resulting distributions as a proxy for uncertainty under data-limited conditions.

This subsampling yields 95% quantile ranges of 17.5–20.6 for CP1 and 9.9–14.7 for CP2. Three (two) of the four CMIP6 models fall within these ranges for CP2 (CP1), indicating that much of the inter-model spread can be attributed to differences in ensemble size. The sensitivity estimates across the three regimes exhibit corresponding variability, with the smallest uncertainty in Regime 1 and the largest in Regime 3. This suggests that sensitivity estimates in Regime 3 are particularly sensitive to limited sample size.
