# Supplementary material for: Regime shifts of AMOC-sea surface temperature relationship
Source: Nat Commun. 2026 Aug 5;17:9416. doi: 10.1038/s41467-026-76149-4 (PMC13538684; doi:10.1038/s41467-026-76149-4)
Supplement: Supplementary file 2 [file 41467_2026_76149_MOESM2_ESM.pdf]

nature portfolio

Peer Review File

Regime shifts of AMOC-sea surface temperature relationship

Corresponding Author: Dr Pengfei Zhang

This file contains all reviewer reports in order by version, followed by all author rebuttals in order by version.

Version 0:

Reviewer comments:

Reviewer #1

(Remarks to the Author)
This manuscript presents a compelling investigation into the non-stationary relationship between Atlantic Meridional Overturning Circulation strength and subpolar North Atlantic sea surface temperature. This is solid work addressing an important question about AMOC-SST coupling across different climate states. The regime-shift framework is novel and the decomposition analysis is carefully executed. I recommend minor revision with the following comments:

Lines 64-71: The discussion of processes shaping North Atlantic SST variability would benefit from incorporating recent observational work on AMOC-SST coupling. Sun et al. (https://iopscience.iop.org/article/10.1088/1748-9326/aacf66) demonstrated that ocean dynamics are essential for reproducing observed interhemispheric SST patterns—slab ocean models lacking AMOC circulation fail to capture the dipole structure. This provides empirical support for your regime framework and helps contextualize why oceanic processes set a baseline even as radiative effects dominate sensitivity changes across regimes. A sentence acknowledging this observational constraint would strengthen the motivation for your decomposition analysis.

Lines 93-100: The identification of two change points at 20.0 Sv (CP1) and 11.5 Sv (CP2) is statistically well-executed, but the manuscript would benefit from reporting uncertainty bounds on these threshold values. While the genetic algorithm efficiently minimizes mean-squared error, bootstrap resampling across the CESM2 ensemble members would quantify how much these values shift under different realizations. This is particularly important for interpreting the CMIP6 spread in Figure 5a—do models genuinely differ in their CP2 location, or do they simply sample different points along a smooth sensitivity curve? A confidence interval of ±1-2 Sv on each change point would contextualize cross-model comparisons.

Lines 134-153: The attribution of the sensitivity tripling to radiative processes is convincing, but the narrative could better distinguish between proximate and ultimate causes. You state that clear-sky longwave flux "supplies roughly two-thirds of the increased sensitivity" and that "basin-wide tropospheric cooling and moisture loss reinforce the longwave anomaly" (lines 145-147). However, what drives this tropospheric response? Is it simply advective cooling from weakened poleward heat transport, or do local circulation changes (subsidence anomalies, altered storm tracks) play a role? A sentence clarifying whether the atmospheric response is largely passive (responding to SST) or includes active dynamical feedbacks would sharpen the mechanistic interpretation.

Lines 183-196: The discussion of CP1 and CP2 mechanisms is rich but somewhat compressed. For CP1, you note the coincidence with aerosol-to-GHG forcing transition but the causal chain remains implicit. Consider expanding: aerosol decline removes a cooling agent that had previously masked GHG warming; this unmasks tropospheric warming and moistening; increased downwelling longwave radiation then amplifies SST responses to AMOC changes. Without this stepwise logic, readers may struggle to connect forcing type to sensitivity regime.

For CP2, the finding that the subpolar ocean transitions from heat source to heat sink is profound but needs elaboration. Does this reflect the shoaling of the overturning cell reducing the depth-integrated heat convergence? Or is it about spatial reorganization—heat convergence shifting from the Labrador/Irminger Seas (captured by your 45-60°N box) to the Nordic Seas (outside the box)? Clarifying this geometry would strengthen the physical narrative

Lines 212-219: The paleoclimate application is insightful but could be strengthened by connecting to observational reconstruction challenges. Sun et al. (https://rmets.onlinelibrary.wiley.com/doi/full/10.1002/joc.6695) showed that AMOC reconstructions from different variables and spatial domains exhibit varying skill, with some approaches leading temperature

variations by several years. Your finding that the SST fingerprint migrates spatially across regimes compounds this challenge—not only does reconstruction method matter, but the optimal method likely changes depending on the background AMOC state.

Lines 310-330: Your choice of maximum overturning below 500 m at 40°N is well-justified and you demonstrate meridional coherence (Supplementary Figure 1f). However, given that the warming hole dynamics unfold at higher latitudes (45-60°N), a brief sensitivity test would be valuable. Does the three-regime structure persist if you instead use maximum overturning at 50°N or 60°N?

Lines 372-395: You describe two approaches for estimating SST sensitivity: direct regression across all simulations versus trend ratios within individual simulations. The methods section would benefit from explicitly stating the assumption underlying the trend-ratio approach—that during periods when AMOC remains within a single regime, the linearity of the AMOC-WHSST relationship ensures that trend ratios equal regression slopes. Also, when you identify time periods for each regime in CESM2-LE (1985-2014, 2014-2070, 2071-2100), how sensitive are the results to these boundary choices? A shift of even 5-10 years could potentially mix signals from adjacent regimes.

The manuscript focuses on forced simulations and ensemble means, but what role does internal variability play in regime transitions? Can stochastic atmospheric forcing push AMOC temporarily across a change point, causing transient regime shifts? Or are the thresholds sufficiently robust that only sustained forcing can drive transitions? This matters for decadal prediction—if internal variability can cause decade-scale regime fluctuations, predicting transition timing becomes much harder. The authors may consider include some discussions on this point.

Reviewer #2

(Remarks to the Author)
Review on 'Regime shifts of AMOC-sea surface temperature relationship' by Fan et al.
In this study, the authors present that the relation between the AMOC (Atlantic Meridional Overturning Circulation) and SSTs (sea surface temperatures) in the NASPG (North Atlantic Subpolar Gyre) is not stationary under different forcing conditions. The results are based on an impressive number of Earth System Model simulations, are very interesting and could be highly relevant. On a few points, the paper could be improved in my opinion, after which the paper should be suitable for publication.

Major comments
1. Description of methodology
I found it hard to follow sometimes what simulations were used for what analysis. I suggest being as explicit as possible which simulations are used when. It is not always clear to me whether only the CESM2-LE is used, or whether all CESM2 simulations are used.

I think it is particularly relevant since the CESM2 simulations use different boundary conditions and different forcing rates and magnitudes. I wonder whether there is a dependence on these aspects. If I look for example at Figure 1, piControl seems to, on visual inspection, follow the sensitivity of regime 2, but has most markers in regime 3. From what I can see this might also be the case for lig127k. See also minor comment 6.

2. Extended analysis
I think that if the analysis is a bit more thorough the paper will improve. One part that I am missing is a more thorough discussion on the effect of changes in deep convection. Especially, as noted already, deep convection locations change over the three regimes with, based on other CESM2 simulations, a shutdown of deep convection in the Labrador Sea, and potentially also in the Irminger Sea in 2100.

I think it would also be interesting to address more extensively the why of the things described in the paragraph starting at line 165.

3. Extend discussion
I'm currently missing how the conclusions and results of this paper fit in the existing literature. I believe the discussion can also be more explicit/extensive on the implactions of the results.

4. Discussion of Lee et al. (2026). I know this paper just came out, but since it is out now, I believe it should be addressed in the revision. The paper focuses on the same region, more or less the same phenomenon, and also uses the CESM2-LE. Their conclusions seem to be different from the conclusion in this paper and should therefore be addressed.

Minor comments
1. Line 56: I suggest to provide the mechanism that causes the warming north of the Gulf Stream to completely explain the SST 'fingerprint'.
2. Line 61: the warming hole is part of the fingerprint, right? I suggest making that (more) explicit in this paragraph.
3. Line 88: is there a reference for the chosen region? Or is this a region specific for this study. I suggest either including a reference or including an explicit statement that this region is used in this study because the paper looks at the warming hole (more explicit than it is now).

4. Line 102: I think it would be interesting to include the values of CP1 and CP2, and the sensitivities somewhere in the manuscript (can be the SI) of the other methods.
5. Line 110: Do we expect the typical dipole pattern in a 1 degree model? Isn't the model resolution too coarse to accurately capture the dynamics of the traditional dipole fingerprint?
6. Line 122: It appears that SSP5-8.5 goes into a new regime in the last decade where SSTs increase, whereas the AMOC still decreases. I think it would be good to comment on this. I think there are also extended simulations for some ESMs past 2100 (see e.g. Drijfhout et al., 2025). It might be insightful to add these here as well.
7. Line 122: I'm not really sure why Fig. S4 is referenced here.
8. Line 184: I suggest explicitly adding that CP1 is forcing dependent just as it was mentioned that CP2 is forcing independent.
9. Line 198: New results are introduced here in the discussion and conclusion. It feels a bit odd. I suggest that the authors consider the placement of these results. I also think that the paper would be stronger if these results were explained more extensively and maybe a bit more analyses were on the why these results in the models, though I can understand if that falls outside the scope of this paper.
10. Discussion general: I miss a part where the use of SST fingerprints on reconstructing the AMOC and projecting a future AMOC collapse is discussed. Discussing the implications of these results on the Ditlevsen & Ditlevsen (2023) paper would be very useful in my opinion.
11. Methods generally: the order of the method section does not follow the order of the main text. This can be a bit confusing, and I suggest reconsidering the order of this section.
12. Line 289: Why is the word 'generally' used here? What are the implications of using the word here?
13. Line 355: This might be tied to my major comment 1. In Fig. S10 it shows that using 3 linear fits has a lower BIC score and should therefore be a better fit based on this score. I understand why the linear 2 option is used and I believe that to be completely valid (you don't need to change this). I do wonder, can the linear 3 option maybe say something about the rate of forcing or the background climate? Also, in Fig. 2abce there seems to be some evidence for an extra regime.
14. Line 439: I think it should be '…; (2) shortwave and (3) longwave …'

Comments on figures and tables:
1. Figure 1: for the inset in (a), would it make more sense to, instead of SST trends, use the trends of SST – GMST as this would directly relate to the other figures? (the same applies to (d)).
2. Figure 5: I find this figure hard to read. Would it improve if the markers of the other models were connected by a line?
3. Figure S1: are the CP determined for each definition, or is the same one plotted in each subpanel? I think the CPs should be determined for each definition separately.
4. Figure S4a: Would it be possible to rotate the plots in (a) a bit such that the landmass of Greenland is positioned such that it resembles the positioning on a 2D map more?
5. Figure S4b: Would it be possible to draw the region in (a) on the map here?
6. Figure S5: doesn't this figure show that the ocean plays a very dominant role?

References
- Lee, S.-K., Kim, D., Lopez, H., & Gomez,F. A. (2026). Future shoaling of the AMOC and its impact on oceanic heat transport to the subpolar North Atlantic. Geophysical Research Letters, 53,e2025GL119487.
- Drijfhout, S., Angevaare, J.R., Mecking, J., van Westen, R.M., and Rahmstorf, S. (2025). Shutdown of norther Atlantic overturning after 2100 following deep mixing collapse in CMIP6 projections. Environmental Research Letters. Doi: 10.1088/1748-9326/adfa3b
- Ditlevsen, P., Ditlevsen, S. Warning of a forthcoming collapse of the Atlantic meridional overturning circulation. Nat Commun 14, 4254 (2023). https://doi.org/10.1038/s41467-023-39810-w

Version 1:

Reviewer comments:

Reviewer #1

(Remarks to the Author)
The authors have responded to all reviewer comments in a thorough and scientifically rigorous manner. The revisions substantially strengthen the manuscript and address the core concerns raised. The addition of subsampling uncertainty analyses, the mechanistic elaborations on CP1 and CP2, and the new discussion on internal variability versus external forcing are particularly valuable contributions. The manuscript is now suitable for acceptance.

Reviewer #2

(Remarks to the Author)
Thank you for the revision. You have covered all my questions and concerns. Congrats on a really nice paper.

Open Access This Peer Review File is licensed under a Creative Commons Attribution 4.0 International License, which permits use, sharing, adaptation, distribution and reproduction in any medium or format, as long as you give appropriate credit to the original author(s) and the source, provide a link to the Creative Commons license, and indicate if changes were made.
In cases where reviewers are anonymous, credit should be given to 'Anonymous Referee' and the source.
The images or other third party material in this Peer Review File are included in the article's Creative Commons license, unless indicated otherwise in a credit line to the material. If material is not included in the article's Creative Commons license and your intended use is not permitted by statutory regulation or exceeds the permitted use, you will need to obtain permission directly from the copyright holder.
To view a copy of this license, visit https://creativecommons.org/licenses/by/4.0/

Response Letter

We thank the reviewers for their insightful and constructive suggestions and have revised the manuscript accordingly. Please find our detailed point-by-point responses along with our revision below. Blue denotes responses to reviewers. Red denotes changes made in the manuscript, and the line number refers to the one shown in the clean manuscript.

Responses to Reviewer #1

This manuscript presents a compelling investigation into the non-stationary relationship between Atlantic Meridional Overturning Circulation strength and subpolar North Atlantic sea surface temperature. This is solid work addressing an important question about AMOC-SST coupling across different climate states. The regime-shift framework is novel and the decomposition analysis is carefully executed. I recommend minor revision with the following comments:

We thank the reviewer for their insightful comments and constructive suggestions.

1. Lines 64-71: The discussion of processes shaping North Atlantic SST variability would benefit from incorporating recent observational work on AMOC-SST coupling. Sun et al. (https://iopscience.iop.org/article/10.1088/1748-9326/aacf66) demonstrated that ocean dynamics are essential for reproducing observed interhemispheric SST patterns—slab ocean models lacking AMOC circulation fail to capture the dipole structure. This provides empirical support for your regime framework and helps contextualize why oceanic processes set a baseline even as radiative effects dominate sensitivity changes across regimes. A sentence acknowledging this observational constraint would strengthen the motivation for your decomposition analysis.

We thank the reviewer for pointing us to this paper. We have revised the text accordingly and added the reference as suggested.

"On decadal and longer timescales, AMOC-related oceanic heat transport (OHT) may shape extratropic North Atlantic SST variability[1,4,15,16]; however, other processes, such as gyre circulation, air–sea interaction, and atmospheric radiation, can also play important roles[17–21]." (L67–70)

Reference:

Sun, C., Li, J., Li, X., Xue, J., Ding, R., Xie, F., & Li, Y. (2018). Oceanic forcing of the interhemispheric SST dipole associated with the Atlantic Multidecadal Oscillation. Environmental Research Letters, 13(7), 074026.

2. Lines 93-100: The identification of two change points at 20.0 Sv (CP1) and 11.5 Sv (CP2) is statistically well-executed, but the manuscript would benefit from reporting uncertainty bounds on

these threshold values. While the genetic algorithm efficiently minimizes mean-squared error, bootstrap resampling across the CESM2 ensemble members would quantify how much these values shift under different realizations. This is particularly important for interpreting the CMIP6 spread in Figure 5a—do models genuinely differ in their CP2 location, or do they simply sample different points along a smooth sensitivity curve? A confidence interval of ±1-2 Sv on each change point would contextualize cross-model comparisons.

We follow the reviewer's suggestion to quantify the uncertainty of detected CPs by subsampling members across the CESM2 ensemble. Specifically, we randomly select a subset of CESM2-LE and SF simulations with sample size n=3 (i.e., the typical number of simulations under most forcing scenarios for other CMIP6 models) and repeat the change point detection procedure 1000 times. The resulting distributions of the two change points are summarized using violin plots and compared with the detected change points in other CMIP6 models (Fig. R1).

Overall, the detected change points are robust against models, as the model spread can be largely explained by the sample size difference and internal variability. For CP2, the uncertainty range of random subsampling is comparable to the model spread and three out of four CMIP6 models lie within the 95% quantile range (9.9–14.7); for CP1, the model spread is larger than the estimated uncertainty range (17.5–20.6), and two CMIP6 models fall outside the range at each tail, suggesting partial influences of structural model differences. Additionally, the sensitivity values exhibit the smallest uncertainty in Regime 1 and the largest uncertainty in Regime 3.

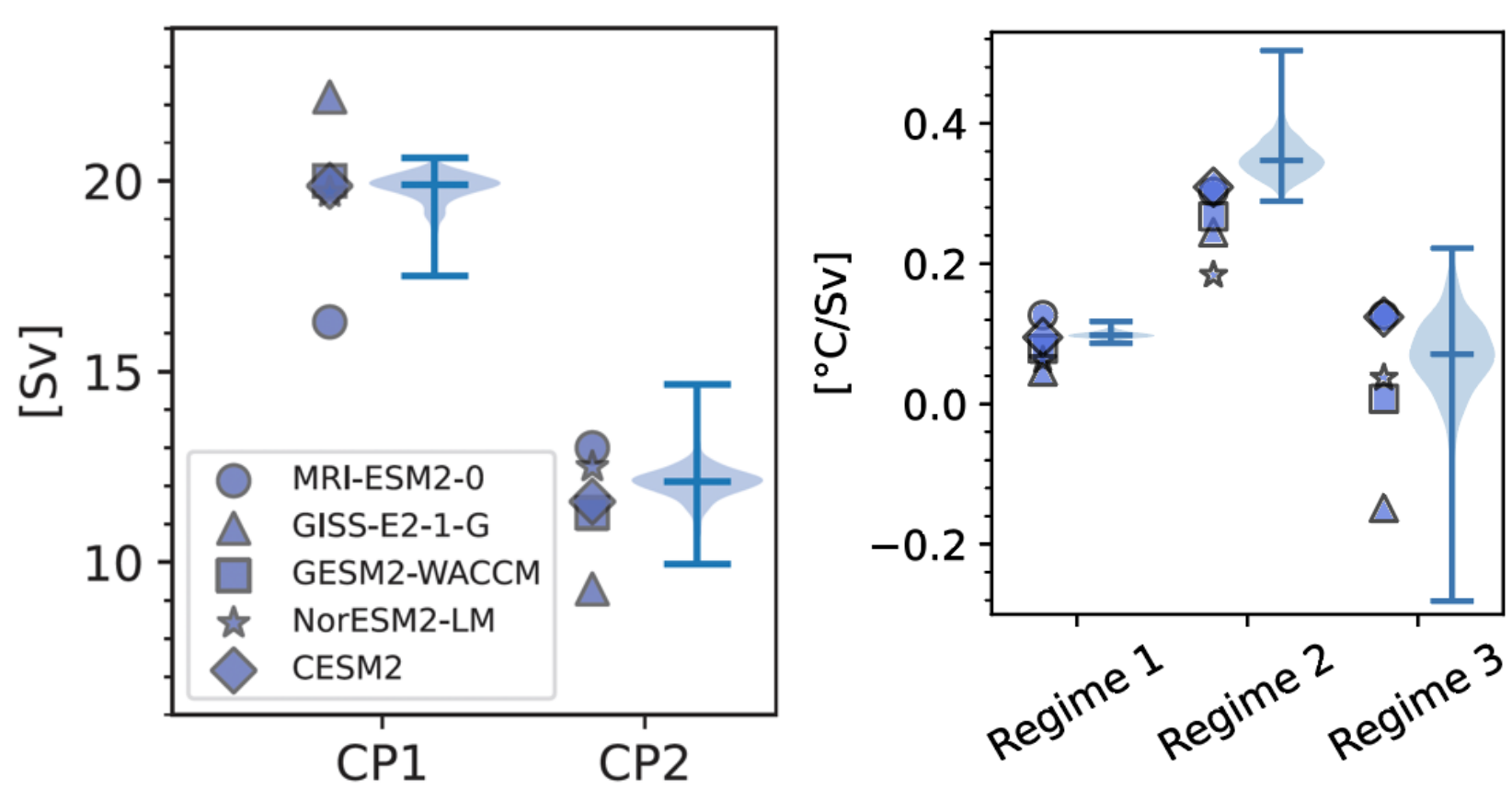

Fig. R1: Values of the two AMOC change points (CP1, CP2; *left*) and sensitivity in the three regimes (*right*) in CESM2 and four other models (scatters). The violin plot (horizontal line: median; shade: density distribution) represents the proxy distribution when change points are detected from a limited size of data (as in four other models) rather than from large ensembles.

We now have replaced Fig. 1b&c with Fig. R1 to provide a context for inter-model comparison and added relevant discussion in Method and Supplementary Information as follows:

"The uncertainty in change-point detection decreases with increasing sample size. However, large ensembles such as those from CESM2 are not available for all models, which limits direct inter-model comparison. To quantify how restricted sample size affects change-point detection, we perform a subsampling analysis using the CESM2 LE and SF simulations. Specifically, we randomly select three members, which is comparable to the ensemble sizes of CMIP6 models, and repeat this procedure 1000 times. The resulting distributions of the two change points and the corresponding sensitivities across the three regimes are summarized by the violin plots in Fig. 1b and 1c. Additional details are provided in the Supplementary Information." (*L442-449*)

"The detection of change points is constrained by sample size (i.e., ensemble size for each forcing scenario). The four CMIP6 models analyzed here have relatively small ensembles (Supplementary Table 2), making it difficult to directly quantify uncertainty in the identified change points and thereby complicating inter-model comparisons. To address this, we use the CESM2 ensembles to indirectly estimate the effect of limited sample size. Specifically, we randomly subsample three members (N = 3) from CESM2 LE and SF simulations 1000 times to match the ensemble sizes of the CMIP6 models, and treat the resulting distributions as a proxy for uncertainty under data-limited conditions.

This subsampling yields 95% quantile ranges of 17.5–20.6 for CP1 and 9.9–14.7 for CP2. Three (two) of the four CMIP6 models fall within these ranges for CP2 (CP1), indicating that much of the inter-model spread can be attributed to differences in ensemble size. The sensitivity estimates across the three regimes exhibit corresponding variability, with the smallest uncertainty in Regime 1 and the largest in Regime 3. This suggests that sensitivity estimates in Regime 3 are particularly sensitive to limited sample size." (*Supplementary Information*)

3. Lines 134-153: The attribution of the sensitivity tripling to radiative processes is convincing, but the narrative could better distinguish between proximate and ultimate causes. You state that clear-sky longwave flux "supplies roughly two-thirds of the increased sensitivity" and that "basin-wide tropospheric cooling and moisture loss reinforce the longwave anomaly" (lines 145-147). However, what drives this tropospheric response? Is it simply advective cooling from weakened poleward heat transport, or do local circulation changes (subsidence anomalies, altered storm tracks) play a role? A sentence clarifying whether the atmospheric response is largely passive (responding to SST) or includes active dynamical feedbacks would sharpen the mechanistic interpretation.

We agree with the reviewer that radiative processes are the proximate cause to the three-regime structure, while the ultimate cause remains uncertain. We have added the following sentences to clarify:

"The non-stationary atmospheric changes with weakening AMOC could be a response to pre-existing SST anomalies or/and involves dynamic coupling between AMOC and atmospheric circulations." (L169–171)

"The three-regime structure originates from non-stationary atmospheric changes with weakening AMOC, while the ultimate causes driving such changes need further studies." (L209–211)

4. Lines 183-196: The discussion of CP1 and CP2 mechanisms is rich but somewhat compressed. For CP1, you note the coincidence with aerosol-to-GHG forcing transition, but the causal chain remains implicit. Consider expanding: aerosol decline removes a cooling agent that had previously masked GHG warming; this unmasks tropospheric warming and moistening; increased downwelling longwave radiation then amplifies SST responses to AMOC changes. Without this stepwise logic, readers may struggle to connect forcing type to sensitivity regime.

We thank the reviewer for pointing this out. We now add more explanations in the revision as follows.

"CP1 (strong to intermediate AMOC) coincides with the shift from aerosol-dominated to greenhouse-gas–dominated forcing (Supplementary Fig. 3a). As aerosol forcing weakens relative to rising greenhouse-gas concentrations, the reduction in cooling agents amplifies greenhouse-gas–induced tropospheric warming and moistening on the global scale. The resulting enhancement in clear-sky longwave radiation causes the AMOC–global mean SST slope (and, to a lesser extent, the AMOC–subpolar North Atlantic SST slope) to shift from near zero to negative values (*Clear-sky LW* in Supplementary Fig. 6a, b). Because the AMOC–subpolar North Atlantic SST slope becomes less negative than the AMOC–global mean SST slope, the AMOC–WHSST relationship steepens markedly after AMOC passes CP1. This tug-of-war between greenhouse gases and aerosols is present across all CESM2 simulations forced with historical (1850–2014) radiative forcings." (L230–241)

5. For CP2, the finding that the subpolar ocean transitions from heat source to heat sink is profound but needs elaboration. Does this reflect the shoaling of the overturning cell reducing the depth-integrated heat convergence? Or is it about spatial reorganization—heat convergence shifting from the Labrador/Irminger Seas (captured by your 45-60°N box) to the Nordic Seas (outside the box)? Clarifying this geometry would strengthen the physical narrative

Here, the spatial region of our interest is the whole subpolar North Atlantic Ocean bounded by 45°N and 60°N. The transition of this region from a net heat source to a net heat sink, diagnosed from the difference in meridional heat transport (MHT) between 45°N and 60°N, is equivalent to the zonally and depth-integrated ocean heat transport (OHT) convergence approaching zero. Such change in depth-integrated OHT convergence may not necessarily reflect the AMOC shoaling effect, because changes in gyre circulation transport and upper-ocean temperature can both play

roles. A quantitative attribution of their respective contributions—for example, using the decomposition framework of Lee et al. (2026)—would be a valuable direction for future research.

We have added clarification on this as follows:

"Additionally, CP2 may reflect a shift of the subpolar ocean from a net heat source to a net heat sink. Near CP2, the OHT convergence between 45°N and 60°N approaches zero (Fig. 2d), as more heat is transported out across the northern boundary than is transported in across the southern boundary. Such changes are associated with the vertical structures of temperature and meridional volume transport by ocean circulations. For example, the decaying phase of the North Atlantic warming hole has been linked with the northward shift of the mid-latitude jet and associated changes in gyre circulations[36]." (L245–251)

As for the Nordic Seas, we have estimated the OHT convergence there as the MHT difference between 65°N and 75°N. The meridional heat transport convergence there generally weakens with AMOC but remains positive across regimes (Fig. R2), suggesting that the mean-state ocean still loses heat to the atmosphere in Regime 3. In other words, the Nordic Seas remains as a heat source while the subpolar North Atlantic (45°–60°N) gradually becomes a heat sink. In this sense, it is a local change rather than a spatial reorganization.

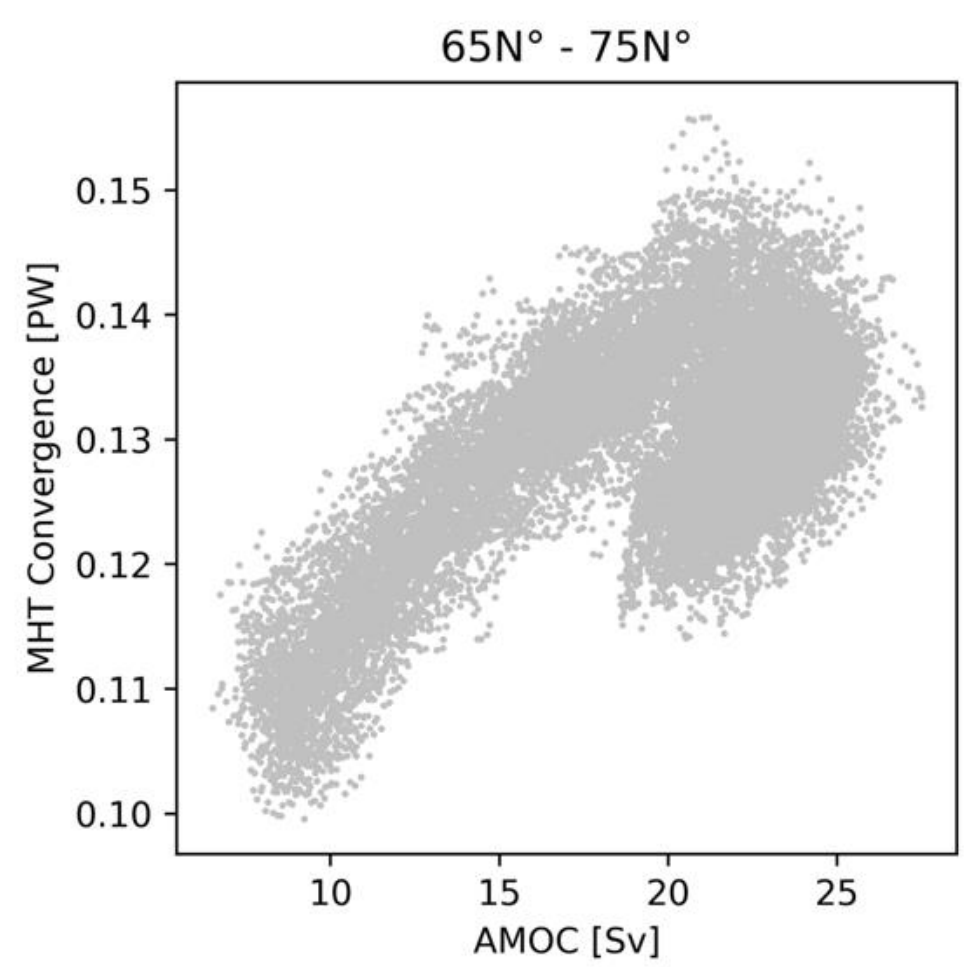

Fig. R2: AMOCI versus North Atlantic meridional heat transport (MHT) difference between 65°N and 75°N in the 100-member CESM2-LE simulations. All data are smoothed by a 15-year moving window.

Reference:

Lee, S.-K., et al. (2026). Future shoaling of the AMOC and its impact on oceanic heat transport to the subpolar North Atlantic. Geophysical Research Letters, 53, e2025GL119487.

6. Lines 212-219: The paleoclimate application is insightful but could be strengthened by connecting to observational reconstruction challenges. Sun et al. (https://rmets.onlinelibrary.wiley.com/doi/full/10.1002/joc.6695) showed that AMOC reconstructions from different variables and spatial domains exhibit varying skill, with some approaches leading temperature variations by several years. Your finding that the SST fingerprint migrates spatially across regimes compounds this challenge—not only does reconstruction method matter, but the optimal method likely changes depending on the background AMOC state.

We thank the reviewer for drawing our attention to this paper. We have revised our text and added the reference as suggested.

"It also suggests that the optimal AMOC indicator might vary with AMOC states, further complexing the reconstruction during the era of instrumental observations[39]." (L269–270)

Reference:

Sun, C., et al. (2021). Atlantic Meridional Overturning Circulation reconstructions and instrumentally observed multidecadal climate variability: A comparison of indicators. International Journal of Climatology, 41(1), 763–778.

7. Lines 310-330: Your choice of maximum overturning below 500 m at 40°N is well-justified and you demonstrate meridional coherence (Supplementary Figure 1f). However, given that the warming hole dynamics unfold at higher latitudes (45-60°N), a brief sensitivity test would be valuable. Does the three-regime structure persist if you instead use maximum overturning at 50°N or 60°N?

We tested the relationship between the warming hole index (WHI) and alternative AMOC indices, including AMOC at 50°N, as shown in Fig. R3. Three regimes consistently emerge, demonstrating the robustness of the three-regime feature.

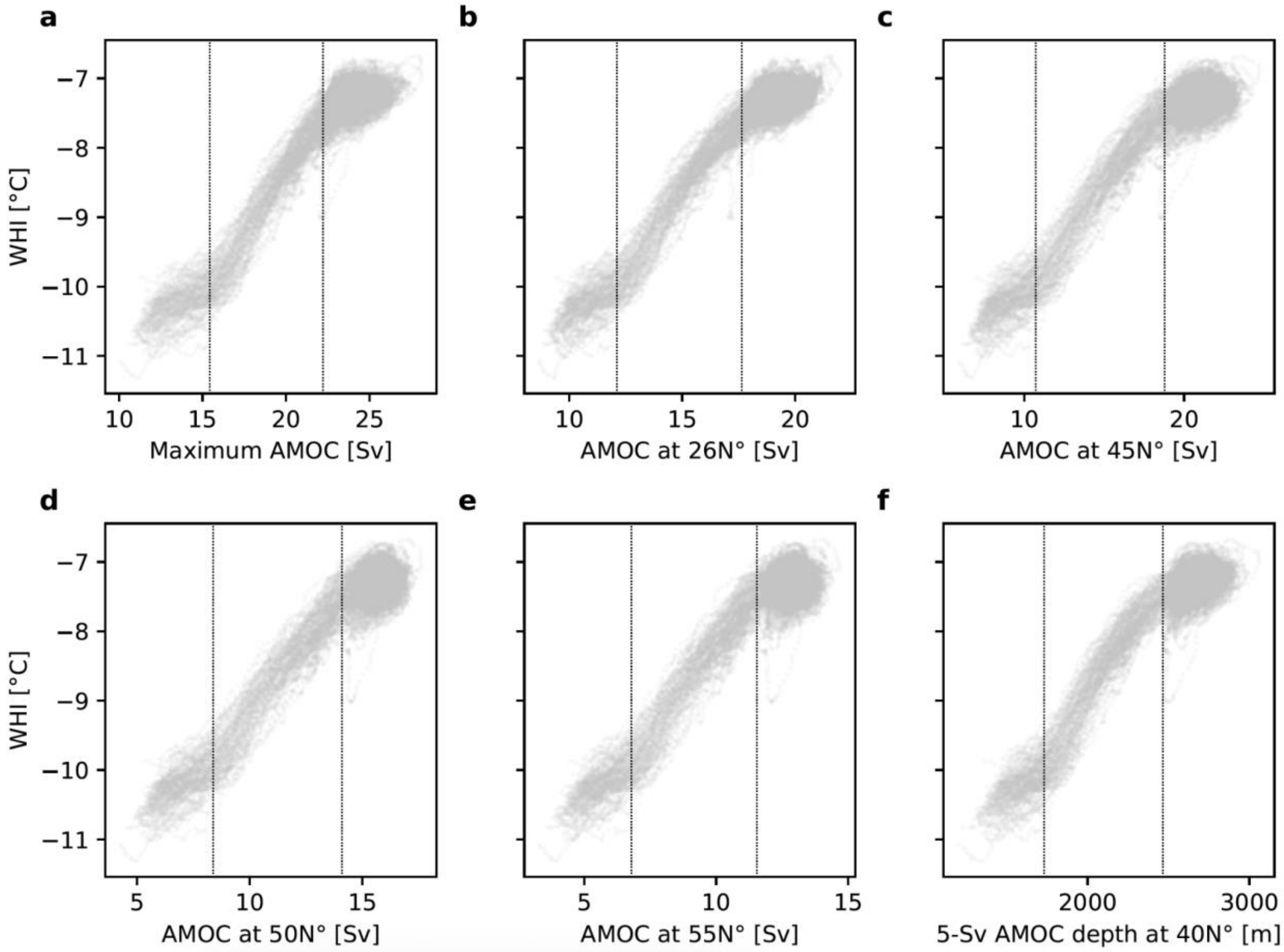

Fig. R3: **Robustness against different AMOC metrics**. **a,** Maximum AMOC across all Northern Hemisphere latitudes (below 500 meters). **b**–**e,** Maximum AMOC at 26°N, 45°N, 50°N, and 55°N, respectively. **f,** The depth of the 5-Sv isoline of volume transport in the upper cell at 40°N. All data are from CESM2-LE simulations and smoothed by a 15-year moving window. Vertical lines denote two corresponding change points, calculated from linear regression between the alternative AMOC metric and the AMOC index used in the manuscript (maximum AMOC at 40°N).

We have added Fig. R3 to our supplementary material as Fig. S2 and made revision in Method (AMOC strength and SST indices) as such:

"Because AMOC variations are meridionally coherent on decadal and longer timescales[47], the maximum overturning stream function at other latitudes (e.g., 50°N), or the maximum across all latitudes, all show two change points in AMOC-WHSST sensitivity, consistent with our AMOC index defined using the maximum overturning stream function at 40°N (Supplementary Fig. 2)." (L382–386)

8. Lines 372-395: You describe two approaches for estimating SST sensitivity: direct regression across all simulations versus trend ratios within individual simulations. The methods section would benefit from explicitly stating the assumption underlying the trend-ratio approach—that during

periods when AMOC remains within a single regime, the linearity of the AMOC-WHSST relationship ensures that trend ratios equal regression slopes. Also, when you identify time periods for each regime in CESM2-LE (1985-2014, 2014-2070, 2071-2100), how sensitive are the results to these boundary choices? A **shift of even 5-10 years c**ould potentially mix signals from adjacent regimes.

To address the reviewer's concern on the regression analysis, we clarified the assumptions made for the trend ratio method and examined the ratios of SST trends to AMOC trends over two alternative time periods for each regime after shifting boundary values by five years. As shown in Fig. R4 (on the next page), the spatial patterns are highly consistent in terms of the key features: the "subpolar cooling and Gulf Stream extension warming" dipole in Regime 1, the intensified whole-basin cooling in Regime 2, and the shift of the cooling center northeastward to the Nordic seas (Fig. 1d; also shown in the below to facilitate comparison).

We now add Fig. R4 to our supplementary material as Fig. S15 and revised the Method section (SST sensitivity to AMOC and its spatial patterns) as follows:

"We also indirectly estimate the sensitivity in individual simulations by calculating the ratio of the SST trend to the AMOC trend over a period. This approach assumes a linear AMOC-SST relationship, which is the case if the whole period falls into a single sensitivity regime. … Similarly, we can identify time periods corresponding to the three regimes in the AAER-only and GHG-only simulations.

These two methods have a major difference. The direct ordinary least square regression between AMOC and SST captures their relationship on all time scales, whereas the trend ratio approach emphasizes on longer time scales, which is our focus in this study. Therefore, when using the direct regression method, all data are smoothed by a 15-year moving window in prior. Despite the difference, the two approaches yield consistent estimates of the "fingerprint" patterns (c.f. Fig. 1d and "*Total*" in Fig. 3a and Supplementary Fig. 7)." (L459–479)

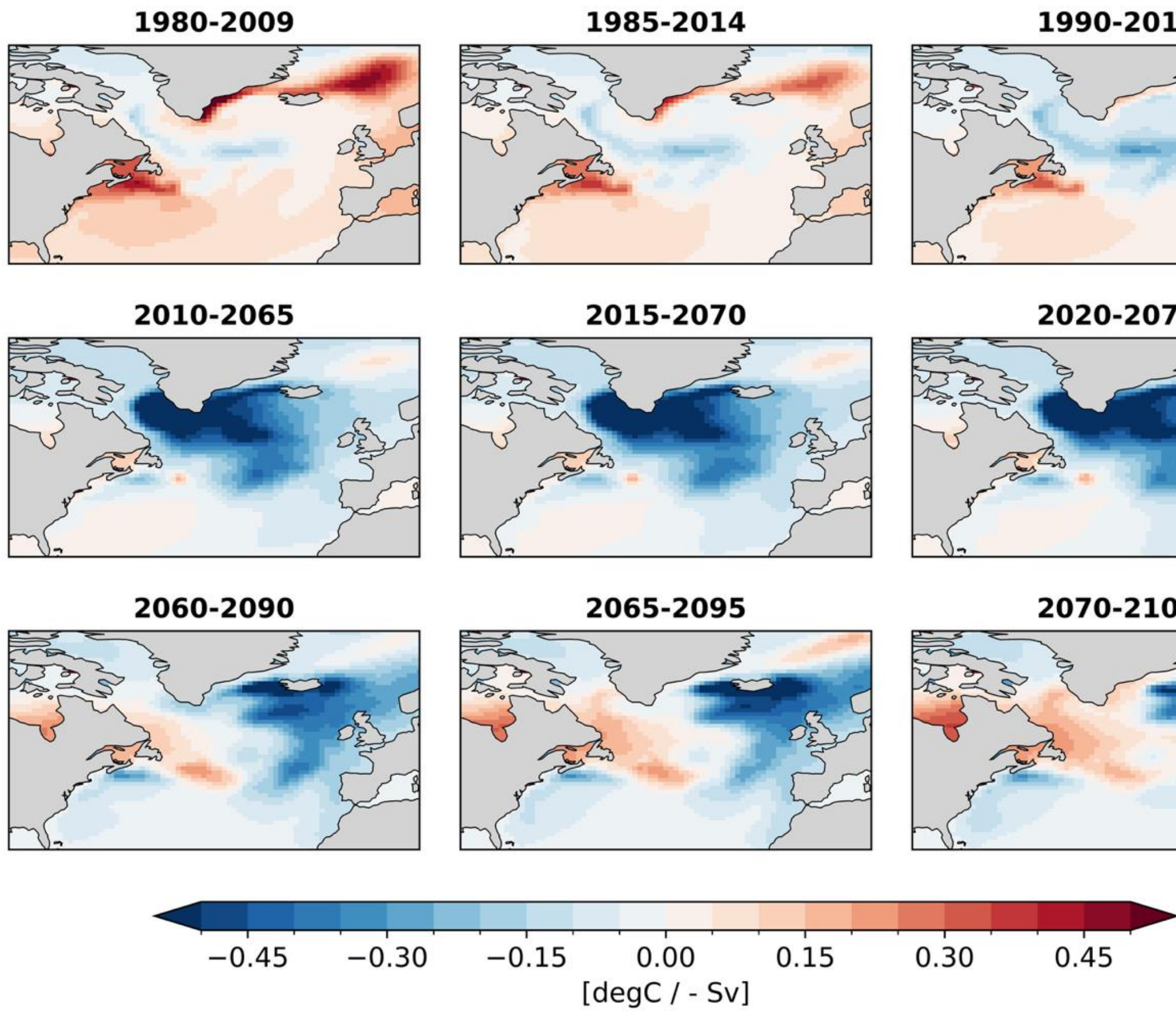

Fig. R4: CESM2-LE mean SST trends (with global mean removed) scaled by AMOC trends over different time periods. The top, middle, and bottom rows correspond to the patterns of SST sensitivity to AMOC weakening in Regime 1, 2, and 3, as shown in Fig. 1d.

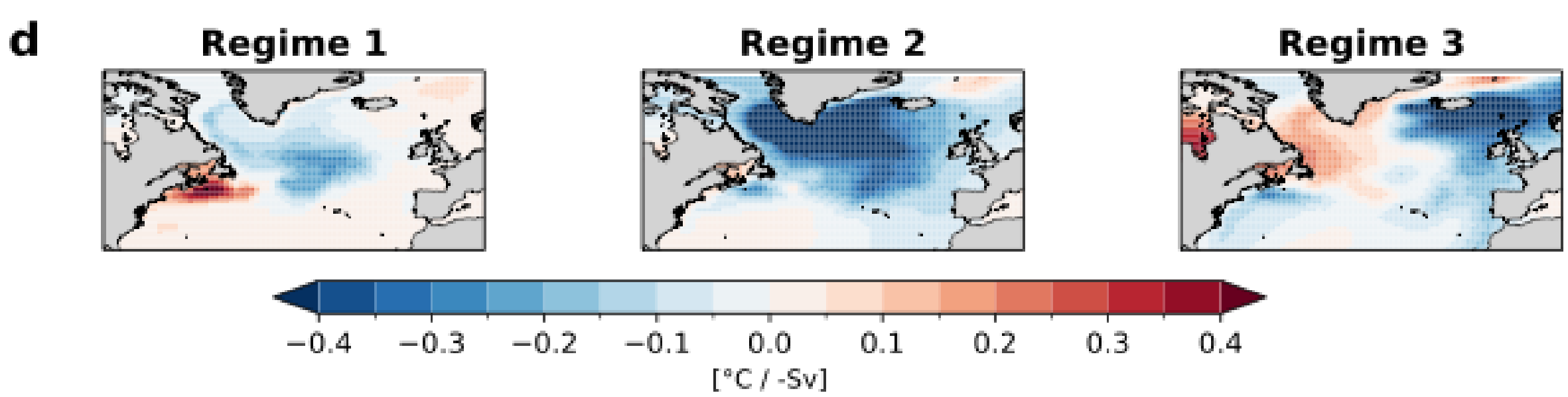

Fig. 1d: Spatial patterns of SST sensitivity to AMOC weakening (-1 °C Sv^{-1}) in the three regimes, estimated by regressing SST anomalies relative to the global mean against AMOC.

9. The manuscript focuses on forced simulations and ensemble means, but what role does internal variability play in regime transitions? Can stochastic atmospheric forcing push AMOC temporarily across a change point, causing transient regime shifts? Or are the thresholds sufficiently robust that only sustained forcing can drive transitions? This matters for decadal prediction—if internal

variability can cause decade-scale regime fluctuations, predicting transition timing becomes much harder. The authors may consider include some discussions on this point.

We agree with the reviewer on the potential effect from internal variability on regime transition. To address this and exclude the effect from external forcings, we have examined the AMOC-WHSST relationship across the 100-member CESM2 LE simulations at years that are close to the two change points (Fig. R5). Here, the regression largely represents the sensitivity on time scales shorter than a decade, which is subject to an influence from internal variability. As internal variability pushes AMOC to weaken and pass CP1, the short-term sensitivity significantly decreases by 0.11 [0.03, 0.20] °C per Sv (the 95% confidence interval estimated by bootstrapping; the distribution is shown by the orange histogram in Fig.R5). This is against the expected sensitivity increase across CP1 (from 0.124 to 0.309 °C per Sv) when external forcings are included. Near CP2, the short-term sensitivity shows no significant change. This suggests that internal variability alone is not likely the cause of the regime shift and sometimes may counteract the effect of the external responses (e.g., near CP1). Therefore, sustained external forcings are needed to drive the transition.

Although internal variability alone cannot cause regime transitions on longer time scales, it still can temporally mask the externally forced responses, making the prediction of regime transition challenging. This can be seen from the large spread across the CESM-LE in the sensitivity peak time (from 2022 to 2068). Additionally, internal variability influences the change point detection, as discussed in our response to Reviewer 1's Comment #2.

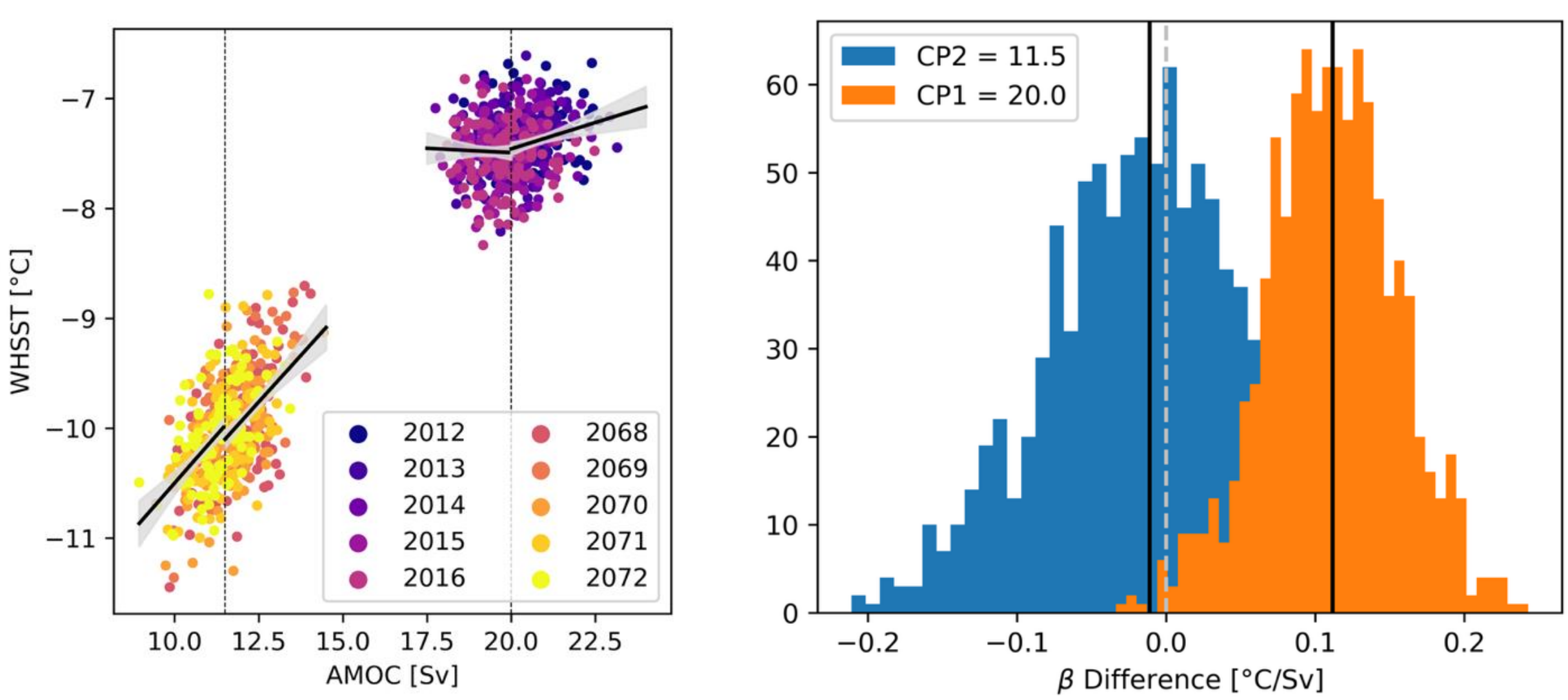

Fig. R5: *(left)* AMOC versus WHSST as in Fig. 1a, except that only the 100-member CESM2-LE data at ten specific years around the change points are plotted. Four linear lines denote the regressions for data points when AMOC is larger or lower than the two change points, and the grey shading represents the 95% confidence interval of the regression line. Vertical lines denote the AMOC change points. *(right)* Distributions of the regression coefficient difference between

before and after when AMOC passes the change points due to internal variability, estimated by bootstrapping the 5-year data near each change point for 1000 times.

We now acknowledge the uncertainty caused by internal variability in revised Discussion as follows:

"As the AMOC-SST sensitivity changes show an inverted-U-shaped trajectory with AMOC, the sensitivity peak time may provide a useful emergent indicator of the regime transition. Nevertheless, internal variability and model uncertainty make it challenging to predict the regime transition (Fig. 4). Internal variability might induce short-term sensitivity change that temporarily masks the externally forced regime shift. As a result, the peak sensitivity can occur as early as in 2021 and as late as in 2067 across the 100-member CESM2-LE (Fig. 4a)." (L254–260)

Responses to Reviewer #2

Review on 'Regime shifts of AMOC-sea surface temperature relationship' by Fan et al.
In this study, the authors present that the relation between the AMOC (Atlantic Meridional Overturning Circulation) and SSTs (sea surface temperatures) in the NASPG (North Atlantic Subpolar Gyre) is not stationary under different forcing conditions. The results are based on an impressive number of Earth System Model simulations, are very interesting and could be highly relevant. On a few points, the paper could be improved in my opinion, after which the paper should be suitable for publication.

We thank the reviewer for their insightful comments and constructive suggestions.

Major comments

1. Description of methodology
I found it hard to follow sometimes what simulations were used for what analysis. I suggest being as explicit as possible which simulations are used when. It is not always clear to me whether only the CESM2-LE is used, or whether all CESM2 simulations are used.

We thank the reviewer for pointing out this potential confusion. We now clarify the simulations used in the revised captions of Fig. 2 and related supplementary figures as:

"Data are from the 100-member full-forcing ensemble (LE) and four single-forcing ensembles (SF-AAER, SF-GHG, SF-BMB, and SF-EE)."

I think it is particularly relevant since the CESM2 simulations use different boundary conditions and different forcing rates and magnitudes. I wonder whether there is a dependence on these aspects. If I look for example at Figure 1, piControl seems to, on visual inspection, follow the

sensitivity of regime 2, but has most markers in regime 3. From what I can see this might also be the case for lig127k. See also minor comment 6.

We agree that although AMOC in *piControl* and *lig127k* falls into Regime 1, the sensitivity might seem more consistent with that in Regime 2 in visual inspection. To assure ourselves that the identified sensitivity regime is robust, we calculated the regression in the two simulations alone. The actual slope value of the AMOC-WHI relationship is 0.190 ([0.179, 0.202]; 95% c.i.) for the *piControl* simulation alone and 0.110 ([0.095, 0.124]) for the *lig127k* simulation alone (green and blue lines in Fig. R6, respectively). Both values are closer to the sensitivity in Regime 1 (0.124) than to the sensitivity in Regime 2 (0.309).

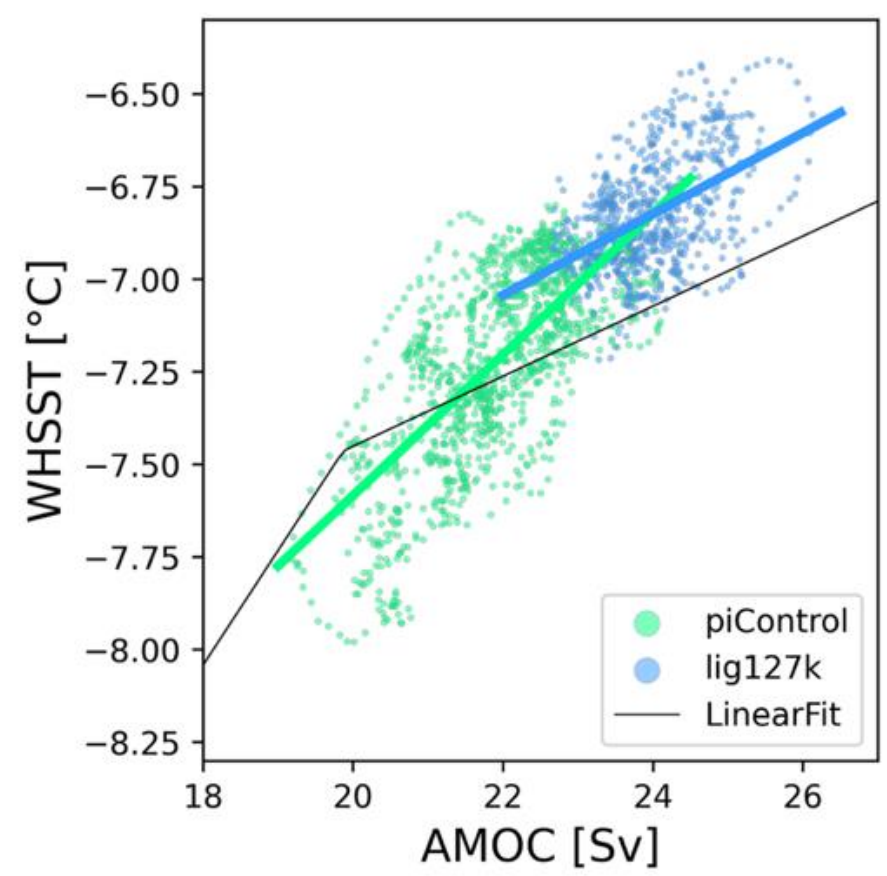

Fig. R6: Similar to Fig. 1a but only data in *piControl* and *lig127k* simulations are plotted.

2. Extended analysis

I think that if the analysis is a bit more thorough the paper will improve. One part that I am missing is a more thorough discussion on the effect of changes in deep convection. Especially, as noted already, deep convection locations change over the three regimes with, based on other CESM2 simulations, a shutdown of deep convection in the Labrador Sea, and potentially also in the Irminger Sea in 2100. I think it would also be interesting to address more extensively the why of the things described in the paragraph starting at line 165.

We thank the reviewer for raising this point. We performed additional analysis of the suggested variables and examined additional literature to explore the mechanisms underlying such changes in the deep convection sites. Overall, our results suggest that the shifts in the locations of weakening deep convection are more likely a consequence than a cause of the regime transition through SST, salinity, and surface wind responses.

In the strong-AMOC regime (1980–2014), AMOC slowdown is associated with weakened deep convection in the Labrador Sea, as indicated by a reduction in January–February–March (JFM) mixed layer depth (MLD; Fig. R7a, b). This decrease might be induced by decreased surface buoyancy forcing due to local warming (orange line in Fig. R9a) and freshening (Fig. R8). The freshening could be caused by freshwater advection from the west Greenland Current (Li et al.

2026) and the Beaufort Gyre (Zhang et al. 2021), which are proposed mechanisms to explain the freshening observed in the past decades.

In the intermediate-AMOC regime (2014–2070), surface freshening intensifies in the northern Labrador Sea and expands eastward into the central subpolar North Atlantic and the Irminger Sea (Fig. R8b). The enhanced freshening in the Labrador Sea and the Irminger Sea could be associated with reduced heat loss (evaporation) as the ocean cools and surface wind decelerates (orange and green lines in Fig. R9a, c; Fig. R10). Sea ice fraction increases as a response to local cooling (Fig. R9b), thus not likely the cause for the freshening. On the contrary, the Greenland–Iceland–Norwegian (GIN) Sea shows no apparent surface cooling, nor apparent changes in surface wind speed, heat loss reduction, freshening, or MLD shoaling.

Near CP2, JFM MLD declines to approximately 100 m, indicating a shutdown of deep convection. Meanwhile, the centers of surface freshening shift northeastward toward the GIN Sea and further intensify (Fig. R8). This could be associated with the shift of surface cooling and resultant heat loss reduction (*Regime 3* in Fig. 1d) and the shift of surface wind deceleration to the GIN Sea (*Regime 3* in Fig. R10). Local sea ice melting (Fig. R9b) can also make additional contributions to the GIN Sea MLD shoaling.

To summarize, changes in the MLD over the three regions across the three regimes are closely linked to SST, salinity, and wind changes. Salinity changes could be a combined effect of freshwater input and reduction in surface heat loss, which is at least partially a response to SST change patterns. In particular, surface wind changes could also be linked to SST change patterns, given the influence of the warming hole pattern on atmospheric circulations (Kramer et al. 2025).

We have added Figs. R7, R8, and R10 to our supplementary material as Figs. S9–S11 and made revisions in Discussion as follows:

"Beyond regime-by-regime contrasts in the energy balance, the locations of deep convection associated with an AMOC slowdown also shift across regimes (Supplementary Fig. 9). In the strong-AMOC regime, suppressed deep convection, as evidenced by reduced winter mixed layer depth, occurs mainly in the Labrador Sea. In the intermediate-AMOC regime, deep convection in the Irminger Sea also weakens. As the AMOC approaches CP2, deep convection effectively shuts down in both regions and subsequently begins to weaken in the Nordic Seas. Changes in North Atlantic deep convection are largely responses to surface buoyancy fluxes and winds associated with atmospheric circulations[33,34]. Although dedicated studies are required to determine the causes of these shifts in convection sites, we hypothesize that they are linked to changes in SST, salinity, and surface winds. As surface cooling and reduced evaporation shift northeastward from Regime 1 to Regime 3, the center of surface freshening follows (Supplementary Fig. 10). Meanwhile, as the AMOC weakens, surface winds decelerate over the Labrador and Irminger Seas in Regime 2, with the center of deceleration shifting to the Greenland–Iceland–Norwegian Sea in Regime 3 (Supplementary Fig. 11), possibly related to the warming hole's influence on atmospheric

circulation[35]. Overall, reduced surface buoyancy forcing from freshening, together with weakened wind forcing, suppresses local deep convection. These concurrent changes reflect coupled ocean–atmosphere–sea-ice interactions, highlighting the complexity of subpolar North Atlantic climate variability." (L212–229)

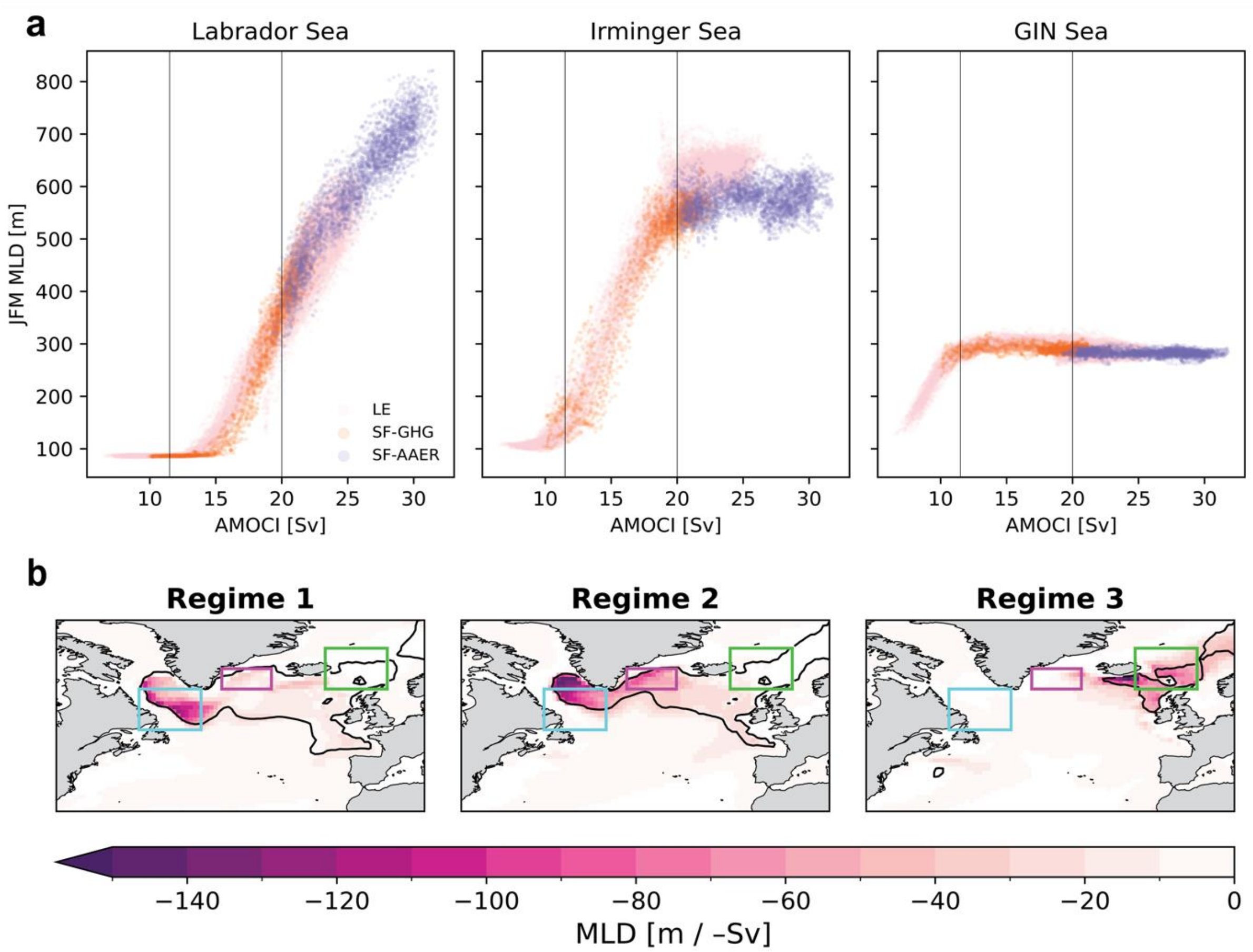

Fig. R7: **AMOC versus January-February-March (JFM) mixed layer depth (MLD)**. (a) AMOC versus JFM MLD averaged over the Labrador Sea (50°–60°N, 40°–60°W), the Irminger Sea (60°–65°N, 28°–40°W), and the Greenland-Iceland-Norwegian (GIN) Sea (60°–70°N, 0°–20°W), which are shown by the cyan, magenta, yellow boxes in (b), respectively. All data are smoothed with a 15-year window. (b) CESM2-LE mean JFM MLD trends scaled by AMOC trends over the 1985–2014, 2015–2070, and 2071–2100 periods, corresponding to Regime 1, 2, and 3, respectively. Black contours in (b) represent the temporal mean JFM MLD isoline of 250 m.

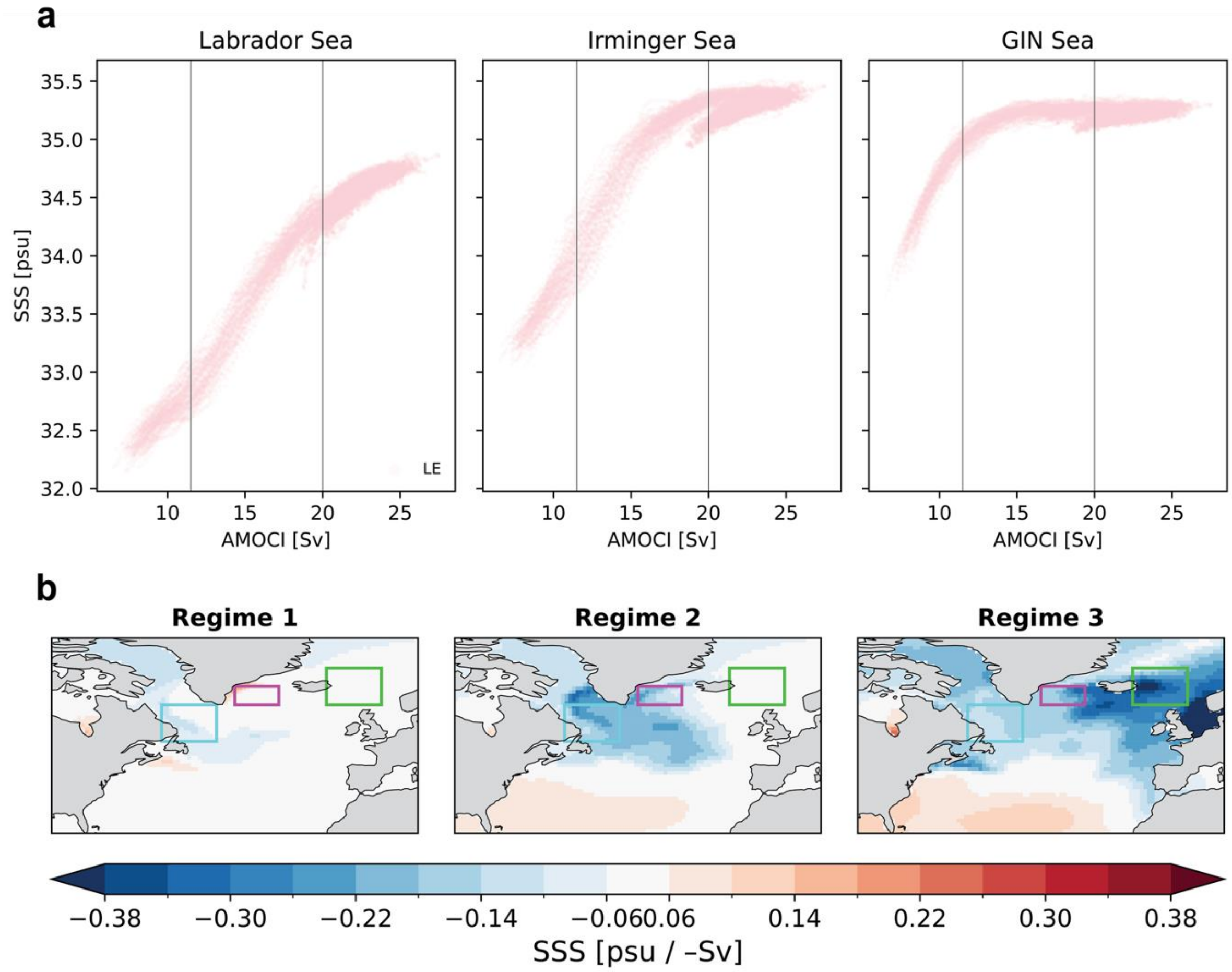

Fig. R8: **AMOC versus JFM sea surface salinity (SSS).** (a) AMOC versus JFM SSS averaged over the Labrador Sea, the Irminger Sea, and the Greenland-Iceland-Norwegian Sea. All data are smoothed with a 15-year window. (b) CESM2-LE mean JFM SSS trends scaled by AMOC trends over the 1985–2014, 2015–2070, and 2071–2100 periods, corresponding to Regime 1, 2, and 3, respectively. Dark navy contours in (b) represent the temporal mean ice fraction area isoline of 50%.

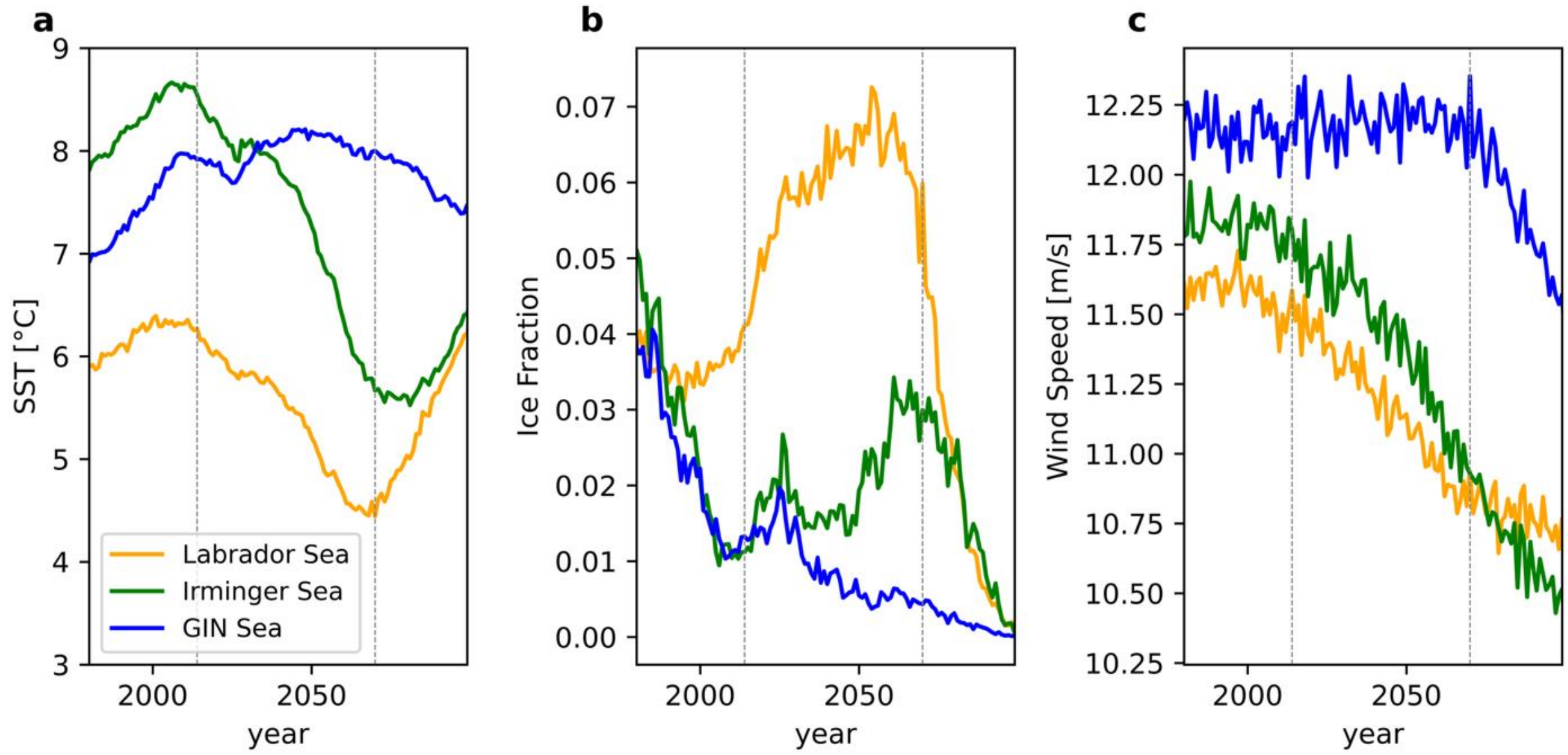

Fig. R9: CESM2-LE ensemble mean (a) SST, (b) ice fraction, and (c) surface wind speed in the Labrador Sea, the Irminger Sea, and the GIN Sea as shown by the boxes in Fig. R7b. Two vertical lines in each panel denote the years when the ensemble mean AMOC passes the two change points.

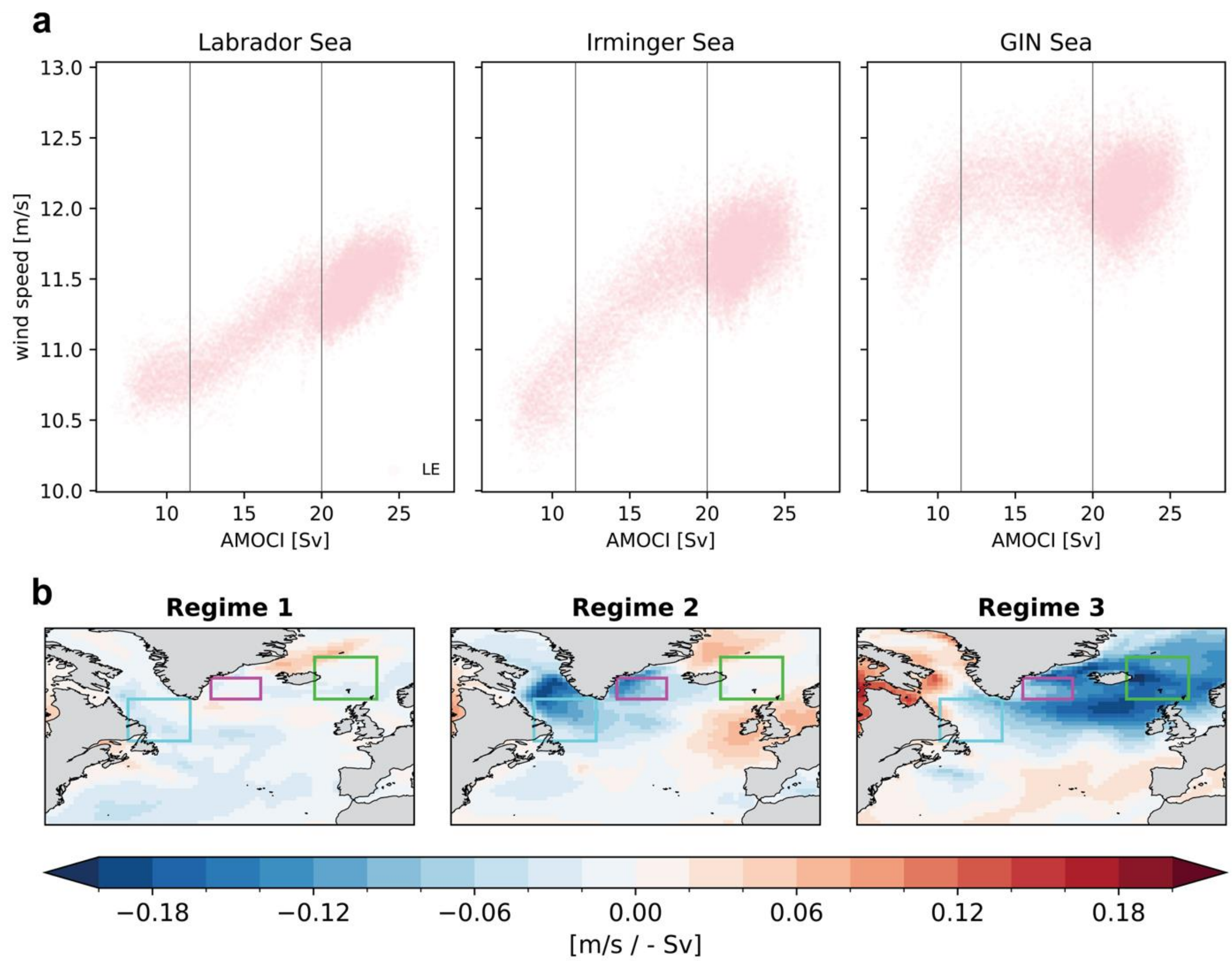

Fig. R10: Similar to Fig. R8, but for surface wind speed.

Reference:

Kramer, S. M., et al. (2025). ColdBlobMIP: A Multi-Model Assessment of the Atmospheric Response to the North Atlantic Warming Hole. Geophysical Research Letters, 52(19).

Li, F., Fu, Y., Petit, T., & Zou, S. (2026). Strengthening of Labrador Sea Overturning Linked to Subsurface Freshening Over Recent Decades. Geophysical Research Letters, 53(1).

Zhang, J., et al. (2021). Labrador Sea freshening linked to Beaufort Gyre freshwater release. Nature Communications, 12(1), 1229.

3. Extend discussion

I'm currently missing how the conclusions and results of this paper fit in the existing literature. I believe the discussion can also be more explicit/extensive on the implications of the results.

We now added a new section to emphasize the implications for the 21st-century North Atlantic climate projections (see our response to Reviewer 2's Minor Comment #9).

“**Projected 21st-century regime shift**

Building on the physical processes identified above, we next examine how the three-regime framework manifests in future climate projections. As AMOC weakens under anthropogenic forcings, the system is expected to evolve along the non-stationary sensitivity trajectory, providing a dynamical basis for interpreting projected evolution of the North Atlantic warming hole. Specifically, in CESM2-LE, as the AMOC on average weakens continuously from about 24 Sv during 1980–2020 to less than 10 Sv in the end of this century, the 40-year moving sensitivity change follows an inverted-U-shaped trajectory (black line in Fig. 4a). Along this trajectory, increasing sensitivity with a weakening AMOC indicates the transition from Regime 1 to Regime 2, whereas decreasing sensitivity with a weakening AMOC marks the transition from Regime 2 to Regime 3.

CMIP6 models generally follow the CESM2-based inverted-U-shaped trajectory, despite differences in AMOC states and sensitivity values. Under the high-emission pathway (SSP5-8.5), 15 CMIP6 models project an AMOC decline together with a North Atlantic warming hole. They all show a tendency of transitioning towards Regime 3 in the end of the century, as denoted by a decrease in the sensitivity as AMOC becomes substantially weak (compare dots and diamonds in Fig. 4a). The projected timing of this transition, estimated by the year of the peak sensitivity, varies widely across the models (2021 to 2067; Fig. 4b). This spread explains 80% of the variance in the projected 2090–2100 WHSST anomalies (Fig. 4b), as an earlier transition to Regime 3 leads to an earlier decay of the warming hole (Fig. 4c). Although identifying precise change points in the real climate system is challenging, the timing of the maximum AMOC-SST sensitivity may provide a useful emergent indicator of the transition between regimes, particularly from the intermediate to weak-AMOC regime.” (L181–203)

We also made revisions in Abstract and Conclusion & Discussion to explicitly emphasize the implications of our study.

“With a projected 21st-century AMOC weakening, a transition to the weak-AMOC regime and a decaying of the North Atlantic warming hole are expected. **The timing of AMOC–SST sensitivity reaching the maximum may serve as an emergent indicator of this transition. Our findings also imply that proxy-based AMOC reconstructions must account for state-dependent and spatially evolving AMOC fingerprints.”** (Abstract)

“**Our findings have direct implications for the 21st-century North Atlantic climate projections.** As the AMOC-SST sensitivity changes show an inverted-U-shaped trajectory with AMOC, the sensitivity peak time may provide a useful emergent indicator of the regime transition. Nevertheless, internal variability and model uncertainty make it challenging to predict the regime transition (Fig. 4). Internal variability might induce short-term sensitivity change that temporarily masks the externally forced regime shift. As a result, the peak sensitivity can occur as early as in

2021 and as late as in 2067 across the 100-member CESM2-LE (Fig. 4a). To constrain models and reduce projection uncertainty, sustained AMOC observations are essential. Higher ocean model resolution is also needed, given its influence on the simulated AMOC mean state[37]. Moreover, scenario-based simulations extended beyond the 21st century[38] may provide additional insights into the AMOC-SST relationship.

Beyond future projections, our findings also have implications for reconstructing AMOC using SST-based proxy records, which often assumes a stationary link between AMOC and its SST fingerprint. The pole-eastward shift of the cooling center identified in this study implies that paleo-reconstructions based on sparse, fixed-site records could underestimate late-stage AMOC variability. It also suggests that the optimal AMOC indicator might vary with AMOC states, further complexing the reconstruction during the era of instrumental observations[39]. The existence of three distinct regimes demonstrates the complexity of inferring dynamical changes from property variability[40]. In addition, uncertainty remains in detecting AMOC's early warning signals in SST[41]. For example, an increased SST variance may reflect a diminishing role of oceanic processes and a strengthening role of atmospheric processes in driving SST variability, rather than an AMOC collapse. A more robust AMOC indicator would therefore require either wider proxy coverage that can follow the migrating SST anomalies, or advanced statistical methods that explicitly account for the uncertainty introduced by the regime shifts." (L254–277)

4. Discussion of Lee et al. (2026). I know this paper just came out, but since it is out now, I believe it should be addressed in the revision. The paper focuses on the same region, more or less the same phenomenon, and also uses the CESM2-LE. Their conclusions seem to be different from the conclusion in this paper and should therefore be addressed.

We thank the reviewer for drawing our attention to this newly published study. We believe that our work and Lee et al. (2026) are complementary rather than contradictory.

First, we would like to emphasize that the focus of Lee et al. (2026) differs from that of our study. Lee et al. (2026) examine the non-stationarity of ocean heat transport (OHT) sensitivity to AMOC, whereas we investigate the non-stationarity of SST sensitivity to both OHT and AMOC. These perspectives are related but distinct. As AMOC weakens, OHT sensitivity to AMOC may increase, as described by Lee et al. (2026). However, SST sensitivity to AMOC can still decrease if SST sensitivity to OHT declines, as suggested by our results.

To support our argument, we conducted an analysis similar to that of Lee et al. (2026) to examine the sensitivity of OHT to AMOC at different latitudes (Fig. R11). Consistent with their findings, we find that the sensitivity of OHT across 40°N to AMOC increases by approximately one third with the transition from a strong to a weak AMOC regime. In contrast, for OHT across 45°N, the southern boundary used in our study, the sensitivity exhibits little change. At higher latitudes, the responses are also heterogeneous: the sensitivity increases at 60°N but decreases at 65°N.

Consequently, from the historical period (1850–2014) to the late 21st century (2071–2100), the sensitivity of net OHT into the region to AMOC evolves differently depending on the latitudinal boundaries considered. It decreases by approximately 15% between 45°N and 60°N (the boundaries used in our study) but increases by approximately 44% between 40°N and 65°N (the boundaries used in Lee et al., 2026). This latitude dependence likely reflects spatial heterogeneity in ocean temperature responses to AMOC weakening, as demonstrated in our analysis. We now discuss Lee et al. (2026) in the revised manuscript as follows:

"Oceanic processes do not explicitly generate the three-regime structure but contribute implicitly. Sensitivity of subpolar North Atlantic OHT convergence to AMOC remains largely constant across regimes (Fig. 2d), despite potential non-stationarity in OHT sensitivity to AMOC across a single latitude[32]." (L172–175)

Reference:

Lee, S.-K., et al. (2026). Future shoaling of the AMOC and its impact on oceanic heat transport to the subpolar North Atlantic. Geophysical Research Letters, 53,e2025GL119487.

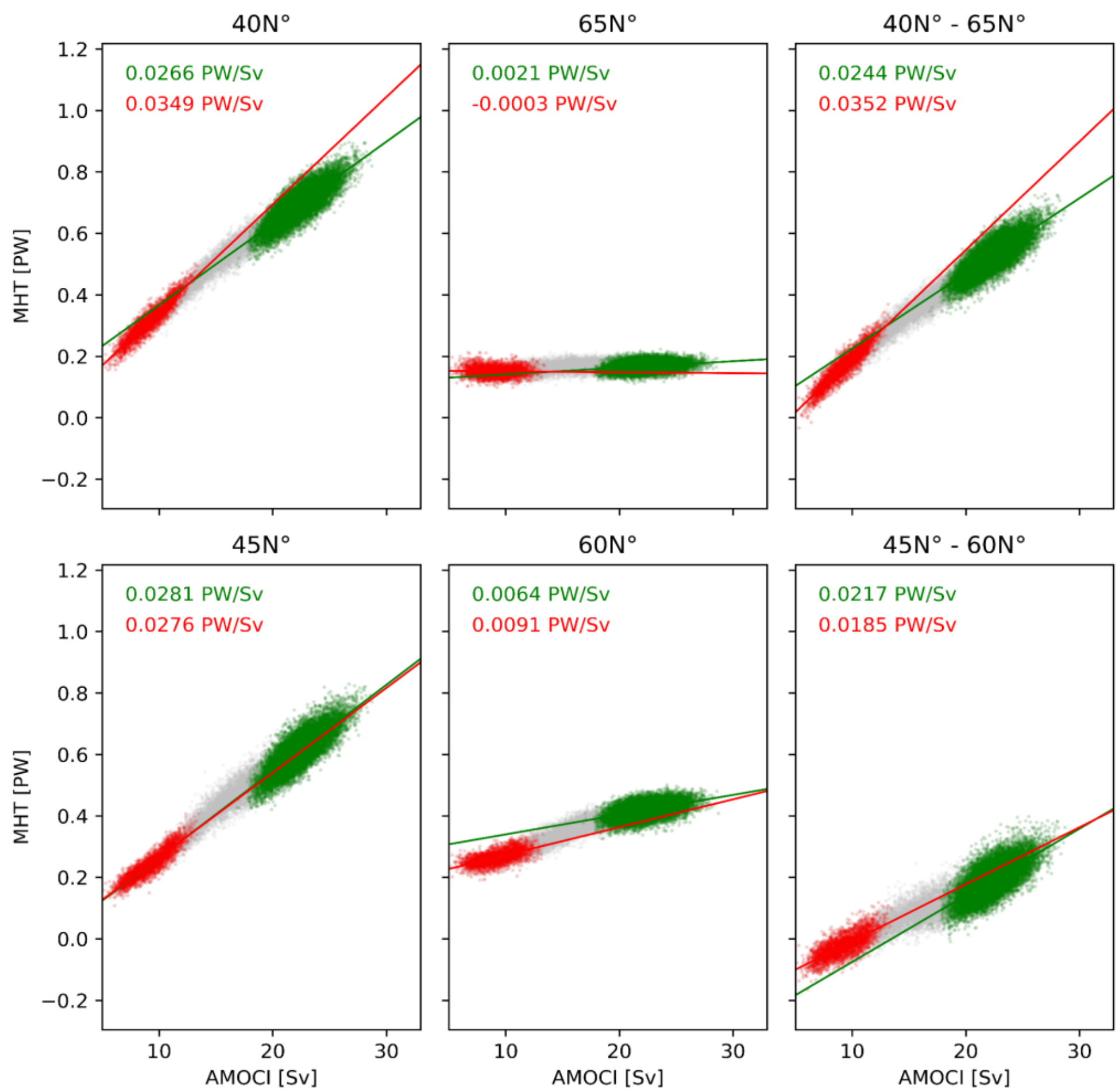

Fig. R11: The AMOC index versus meridional ocean heat transport at different latitudes and the difference between two latitudes in the 100-member CESM2-LE simulations. (Lee et al. 2026 have used 80 CESM2-LE members.) Green and blue dots denote 1850–2014 data and 2071–2100 data, respectively, similar to Fig. 1e of Lee et al. (2026). Linear lines represent corresponding linear regression, and values of the regression coefficients are shown at the top left corner at each panel.

Minor comments

1. Line 56: I suggest providing the mechanism that causes the warming north of the Gulf Stream to completely explain the SST 'fingerprint'.

We now provide an explicit explanation in the revised manuscript.

"Anomalously weak or strong AMOC is expected to result in a dipole SST anomaly "fingerprint" by modulating poleward heat transport on decadal and longer time scales. For example, the subpolar North Atlantic cools as weakening AMOC decreases poleward ocean heat transport in the upper ocean, whereas the western subtropics warms due to associated northward migration of the Gulf Stream[8,9]." (L53–57)

2. Line 61: the warming hole is part of the fingerprint, right? I suggest making that (more) explicit in this paragraph.

The fingerprint specifically refers to AMOC's imprint on SST anomalies; while the warming hole is the long-term SST change (i.e., a lack of warming) in the subpolar North Atlantic, which may also be induced by factors other than AMOC. We have revised the manuscript to clarify the difference between the two as suggested:

"Anomalously weak or strong AMOC is expected to result in a dipole SST anomaly "fingerprint" by modulating poleward heat transport on decadal and longer time scales. For example, the subpolar North Atlantic cools as weakening AMOC decreases poleward ocean heat transport in the upper ocean, whereas the western subtropics warms due to associated northward migration of the Gulf Stream[8,9]." (L53–57)

"For example, the observed North Atlantic "warming hole"–a lack of long-term warming trend in the subpolar North Atlantic SST compared to global means (see the inserted map in Fig. 1a)–is hypothesized as …" (L61–63)

3. Line 88: is there a reference for the chosen region? Or is this a region specific for this study. I suggest either including a reference or including an explicit statement that this region is used in this study because the paper looks at the warming hole (more explicit than it is now).

We have added an explicit statement about our choice of the warming hole region:

"... warming hole SST index (WHSST) defined as the SST difference between a regional mean in the subpolar North Atlantic **where the observed warming hole is largely located (45°–60°N, 20°–50°W; inserted map in Fig. 1a)** and the global mean..." (L91–92)

4. Line 102: I think it would be interesting to include the values of CP1 and CP2, and the sensitivities somewhere in the manuscript (can be the SI) of the other methods.

We have added the values of CP1 and CP2 in the caption of Supplementary Fig. 1.

"The vertical dotted lines represent two change points detected for each SST metric separately. They are 11.5 Sv and 12.0 Sv in (a; the ones adapted in the manuscript), 12.06 Sv and 19.80 Sv in (b), 12.09 Sv and 19.43 Sv in (c), and 11.90 Sv and 19.91 Sv in (d)."

5. Line 110: Do we expect the typical dipole pattern in a 1 degree model? Isn't the model resolution too coarse to accurately capture the dynamics of the traditional dipole fingerprint?

The SST fingerprint of AMOC variability is a large-scale feature spanning the entire subpolar and subtropical basin. Thus, 1-degree horizontal resolution should be sufficient to capture the fingerprint. Even though later studies pointed out the improvements in simulating AMOC with

increased model resolution (e.g., Jackson et al. 2020), the large-scale SST anomaly pattern can be sufficiently represented in the workhorse global climate models. For example, GFDL-CM2.1 used in Zhang (2008) has an oceanic horizontal resolution of 1°, and Zhu & Cheng (2024) use an ocean model with a nominally 3° horizontal resolution. Also, modeling centers face a trade-off between the model resolution and the ensemble size given limited computational resources. We have acknowledged the potential influence of model resolutions in the revised manuscript as follows.

"To constrain models and reduce projection uncertainty, sustained AMOC observations are essential. Higher ocean model resolution is also needed, given its influence on the simulated AMOC mean state[37]." (L260–262)

Reference:

Jackson, L. C., Roberts, M. J., Hewitt, H. T., Iovino, D., Koenigk, T., Meccia, V. L., Roberts, C. D., Ruprich-Robert, Y., & Wood, R. A. (2020). Impact of ocean resolution and mean state on the rate of AMOC weakening. Climate Dynamics, 55(7–8), 1711–1732.

Zhang, R. (2008). Coherent surface-subsurface fingerprint of the Atlantic meridional overturning circulation. *Geophysical Research Letters*, *35*(20), L20705.

Zhu, C., & Cheng, L. (2024). Sensitivity of AMOC Fingerprints Under Future Anthropogenic Warming. *Geophysical Research Letters*, *51*(3).

6. Line 122: It appears that SSP5-8.5 goes into a new regime in the last decade where SSTs increase, whereas the AMOC still decreases. I think it would be good to comment on this. I think there are also extended simulations for some ESMs past 2100 (see e.g. Drijfhout et al., 2025). It might be insightful to add these here as well.

We agree with the reviewer that examining extended simulations would provide insights on Regime 3 and beyond. However, there are no extended SSP585 simulations from CESM2. We have added discussion on the research opportunities provided by such extended simulations and referenced Drijfhout et al. (2025) there.

"Moreover, scenario-based simulations extended beyond the 21st century[38] may provide additional insights into the AMOC-SST relationship." (L262–264)

Reference:

Drijfhout, S., Angevaare, J. R., Mecking, J., van Westen, R. M., & Rahmstorf, S. (2025). Shutdown of northern Atlantic overturning after 2100 following deep mixing collapse in CMIP6 projections. *Environmental Research Letters*, *20*(9), 094062.

7. Line 122: I'm not really sure why Fig. S4 is referenced here.

We reference Fig. S4a for the evolution of the temperature change pattern from 1985 to 2100. We have revised Fig. S4 (Fig. S5 now) to avoid confusion.

8. Line 184: I suggest explicitly adding that CP1 is forcing dependent just as it was mentioned that CP2 is forcing independent.

We have revised the text as suggested.

9. Line 198: New results are introduced here in the discussion and conclusion. It feels a bit odd. I suggest that the authors consider the placement of these results. I also think that the paper would be stronger if these results were explained more extensively and maybe a bit more analyses were on the why these results in the models, though I can understand if that falls outside the scope of this paper.

To address the first point, we have moved these results to a newly added section titled "Projected 21st-century regime shift" as suggested and added more discussion (see our response to your Major Comment #3).

As for the second point, although we leave detailed analysis of sensitivity changes in other models for future studies, we have now included a new reference on the decaying of the North Atlantic warming hole in another model (MPI-ESM1.1 in Ghosh et al. 2023):

"… For example, the decaying phase of the North Atlantic warming hole has been linked with the northward shift of the mid-latitude jet and associated changes in gyre circulations[36]." (L250–251)

Reference:

Ghosh, R., et al. (2023). Two Distinct Phases of North Atlantic Eastern Subpolar Gyre and Warming Hole Evolution under Global Warming. *Journal of Climate*, 36(6), 1881–1894.

10. Discussion general: I miss a part where the use of SST fingerprints on reconstructing the AMOC and projecting a future AMOC collapse is discussed. Discussing the implications of these results on the Ditlevsen & Ditlevsen (2023) paper would be very useful in my opinion.

We now discuss SST fingerprints as an early warning indicator of AMOC collapse and referenced Ditlevsen & Ditlevsen (2023) in the revised manuscript.

"The existence of three distinct regimes demonstrates the complexity of inferring dynamical changes from property variability[40]. In addition, uncertainty remains in detecting AMOC's early warning signals in SST[41]. For example, an increased SST variance may reflect a diminishing role of oceanic processes and a strengthening role of atmospheric processes in driving SST variability, rather than an AMOC collapse." (L270–275)

Reference:

Ditlevsen, P., & Ditlevsen, S. (2023). Warning of a forthcoming collapse of the Atlantic meridional overturning circulation. *Nature Communications*, *14*(1), 4254.

11. Methods generally: the order of the method section does not follow the order of the main text. This can be a bit confusing, and I suggest reconsidering the order of this section.

We have doubled checked the method sections and the way they are referenced throughout the manuscript to make sure they are in order.

12. Line 289: Why is the word 'generally' used here? What are the implications of using the word here?

We have used the word "generally" as the second half of CESM2-LE have incorporated some modifications to biomass burning. Details are documented in https://www.cesm.ucar.edu/community-projects/lens2 and Rodgers et al. (2021) we have referenced.

Reference:

Rodgers, K. B., et al. (2021). Ubiquity of human-induced changes in climate variability. *Earth System Dynamics*, *12*(4), 1393–1411.

13. Line 355: This might be tied to my major comment 1. In Fig. S10 it shows that using 3 linear fits has a lower BIC score and should therefore be a better fit based on this score. I understand why the linear 2 option is used and I believe that to be completely valid (you don't need to change this). I do wonder, can the linear 3 option maybe say something about the rate of forcing or the background climate? Also, in Fig. 2abce there seems to be some evidence for an extra regime.

We agree with the reviewer that the additional change point (~25 Sv) in the linear 3 model seems to show an extra regime. However, this change point is not likely a physical one in SST-AMOC sensitivity. It is associated with the shift in aerosol-forcing from strengthening to weakening around the 1980s (Supplementary Fig. 4a) and likely reflects a time lag between the SST and AMOC response to the change in aerosol forcing. The patterns of the ratio of SST trends to AMOC trends before 1980 and after 1980 are consistent with each other and Regime 1 in terms of the "subpolar cooling with Gulf Stream warming" dipole and the dominance of oceanic processes (Supplementary Fig. 7a). Therefore, we think the additional change point around 25 Sv is not physically relevant here.

14. Line 439: I think it should be '…; (2) shortwave and (3) longwave …'

Fixed!

Comments on figures and tables:

1. Figure 1: for the inset in (a), would it make more sense to, instead of SST trends, use the trends of SST – GMST as this would directly relate to the other figures? (the same applies to (d)).

We have plotted trends of SST minus GMSST (Fig. R12) as suggested and revised Fig. 1a accordingly.

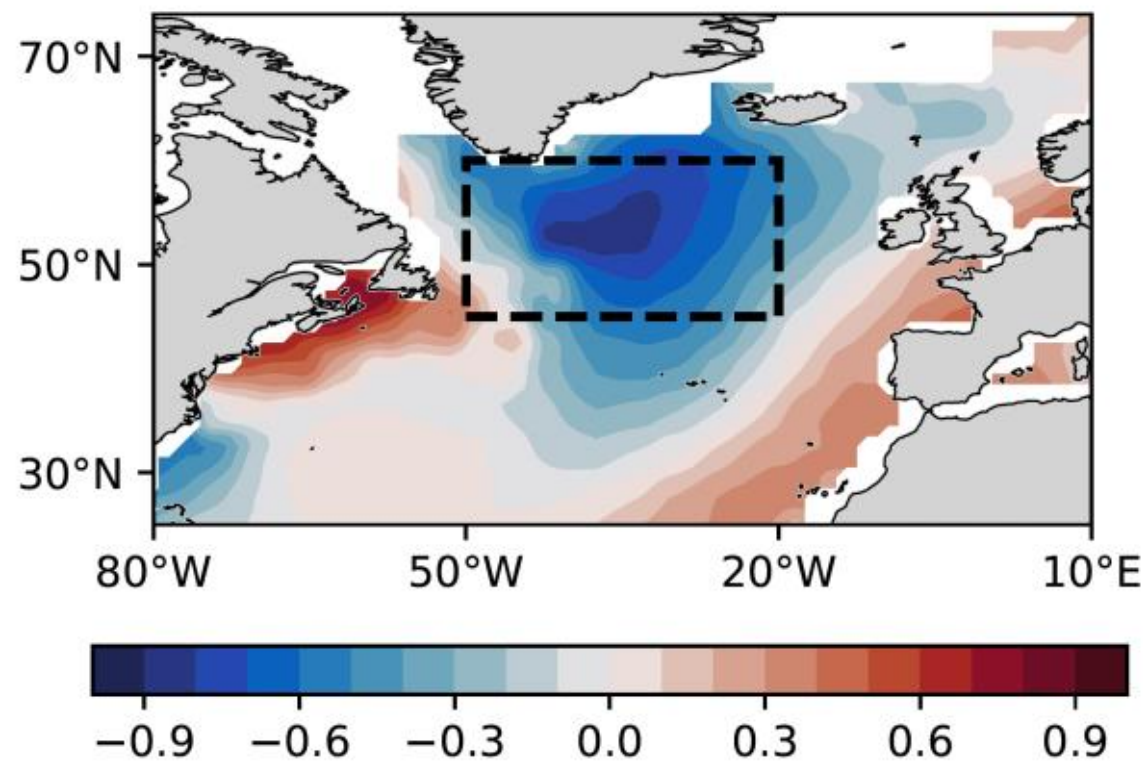

Fig. R12: The inset, modified from Fan et al. (2024), shows observed SST trends minus global mean SST trend (1900–2014, °C / century) based on the average of three SST datasets.

2. Figure 5: I find this figure hard to read. Would it improve if the markers of the other models were connected by a line?

We tried plotting lines for each model, but the effect turned out to be too noisy. The trajectory of AMOC-WHSST sensitivity in one model simulation can fluctuate irregularly because of effect from internal variability. On the contrary, the quadratic polynomial fit of the AMOC-WHSST sensitivity and the AMOC state across the CESM2-LE shows a smooth inverted-U-shaped trajectory, serving as a base line for inter-model comparison. Moreover, we want to emphasize the maximum sensitivity, rather than the sensitivity trajectory, for each model. Given these considerations, we decided to display CESM2-LE data in a fitted line and data from other models in scatters.

3. Figure S1: are the CP determined for each definition, or is the same one plotted in each subpanel? I think the CPs should be determined for each definition separately.

We have detected the two change points for each definition separately as suggested. The subpolar gyre SST minus global mean SST, following Caesar et al. (2018), yields 12.06 Sv and 19.80 Sv, respectively; the subpolar North Atlantic SST minus northern hemisphere mean SST yields 12.09 Sv and 19.43 Sv, respectively; and the subpolar North Atlantic SST minus North Atlantic mean SST yields 11.90 Sv and 19.91 Sv, respectively. We have revised Fig. S1 and its caption correspondingly.

Reference:

Caesar, L., Rahmstorf, S., Robinson, A., Feulner, G., & Saba, V. (2018). Observed fingerprint of a weakening Atlantic Ocean overturning circulation. *Nature*, *556*(7700), 191–196.

4. Figure S4a: Would it be possible to rotate the plots in (a) a bit such that the landmass of Greenland is positioned such that it resembles the positioning on a 2D map more?

We have changed the original 3-D plots to multiple 2-D plots for clearer visualization, as shown by Fig. R13.

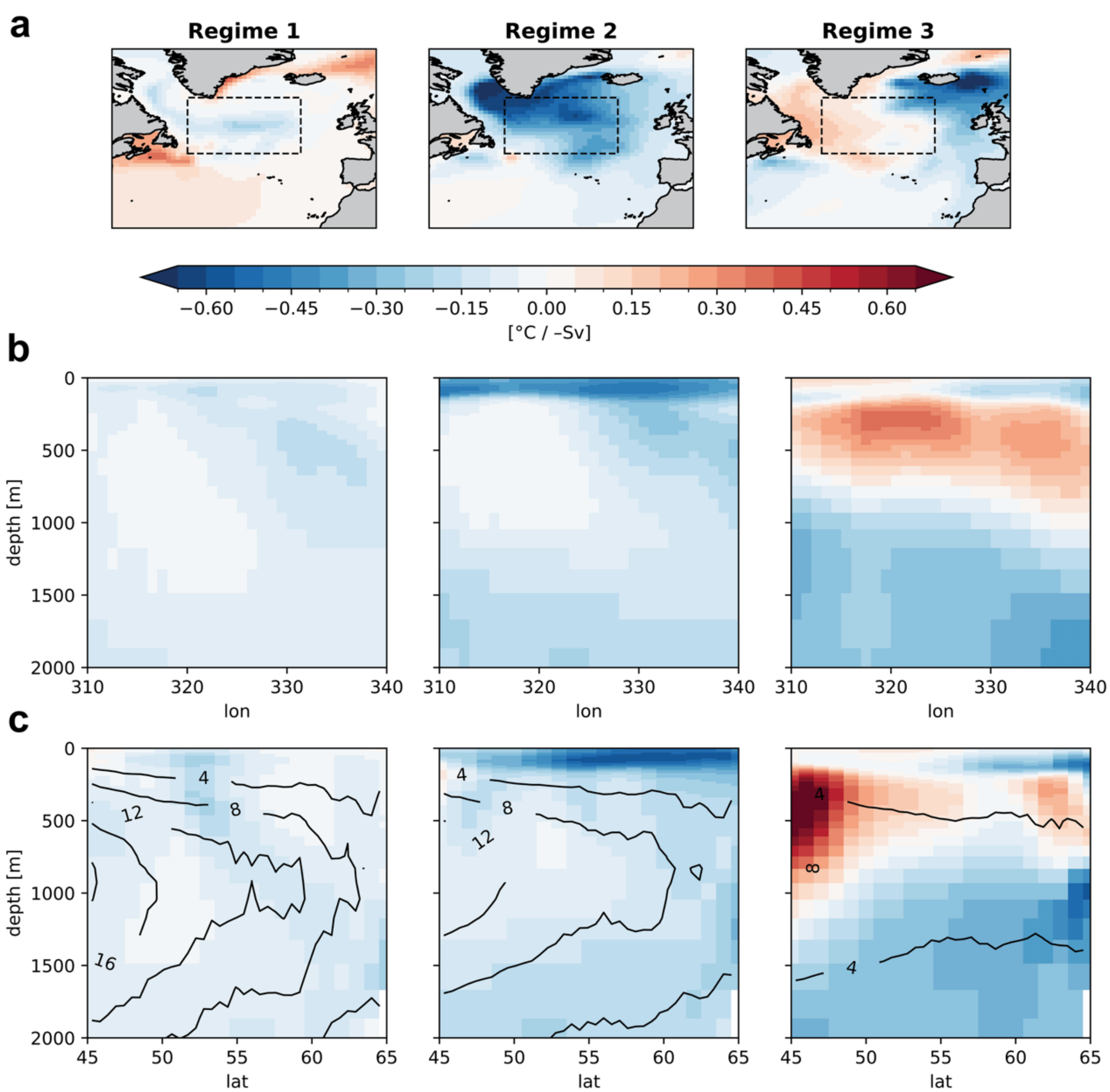

Fig. R13: Changes in ocean potential temperatures. **a**, The ensemble-mean SST trends (with the global mean removed) over 1985–2014 (Regime 1), 2015–2070 (Regime 2), and 2071–2100 (Regime 3) in the CESM2-LE simulations. **b**, Same as (a) but for the zonal section of the meridional mean ocean temperatures in the subpolar North Atlantic domain (denoted by the box in (a)). **c**, Same as (b) but for the meridional section of the zonal mean ocean temperatures in the

subpolar North Atlantic. All trends are scaled by the rate of AMOC weakening over the corresponding period. The contours in (c) denote the Atlantic overturning stream function climatology over the three periods and are shown in a 4-Sv interval.

5. Figure S4b: Would it be possible to draw the region in (a) on the map here?

Done.

6. Figure S5: doesn't this figure show that the ocean plays a very dominant role?

Yes, the ocean plays a very dominant role in the sense of the absolute contribution from OHT convergence to SST anomalies. However, this contribution is largely balanced out by surface turbulent heat loss and is not important in causing the three-regime features. In other words, its absolute contribution to SST anomalies remains largely constant, but its relative importance decreases and becomes out shadowed by radiative processes as AMOC weakens. We have revised the manuscript to emphasize this point:

"Oceanic processes do not explicitly generate the three-regime structure but contribute implicitly. Sensitivity of subpolar North Atlantic OHT convergence to AMOC remains largely constant across regimes (Fig. 2d), despite potential non-stationarity in OHT sensitivity to AMOC across a single latitude[32]. Moreover, OHT convergence and turbulent heat loss largely cancel across all regimes, setting a near-neutral baseline that barely affects the AMOC–WHSST slope (Fig. 2a). Nevertheless, oceanic import makes indirect contribution to the three-regime behavior through air-sea interaction[22–25]. By producing substantial SST anomalies, OHT convergence drives compensating adjustments in surface turbulent heat fluxes (Supplementary Fig. 6) that are associated with non-stationary atmospheric responses." (L172–180)

Following are our responses to the reviewers' comments.

Reviewer #1 (Remarks to the Author):

The authors have responded to all reviewer comments in a thorough and scientifically rigorous manner. The revisions substantially strengthen the manuscript and address the core concerns raised. The addition of subsampling uncertainty analyses, the mechanistic elaborations on CP1 and CP2, and the new discussion on internal variability versus external forcing are particularly valuable contributions. The manuscript is now suitable for acceptance.

Reply: We thank the reviewer for their recognition of our work! Their constructive suggestions have helped improve the robustness of our findings.

Reviewer #2 (Remarks to the Author):

Thank you for the revision. You have covered all my questions and concerns. Congrats on a really nice paper.

Reply: We thank the reviewer for their positive feedback! Their questions and concerns have helped improve clarity of our paper.
